# Homogeneous Gold Catalysis Using Complexes Recovered from Waste Electronic Equipment

**DOI:** 10.1021/acssuschemeng.2c04092

**Published:** 2022-11-17

**Authors:** Sean McCarthy, Oriane Desaunay, Alvin Lee Wei Jie, Maximilian Hassatzky, Andrew J. P. White, Paola Deplano, D. Christopher Braddock, Angela Serpe, James D. E. T. Wilton-Ely

**Affiliations:** † Department of Chemistry, Imperial College, Molecular Sciences Research Hub, White City Campus, London W12 0BZ, U.K.; ‡ Department of Chemical and Soil Sciences, University of Cagliari, Monserrato, 09042 Cagliari, Italy; § Department of Civil and Environmental Engineering and Architecture (DICAAR), INSTM Unit, University of Cagliari, Via Marengo 2, 09123 Cagliari, Italy; ∥ Environmental Geology and Geoengineering Institute of the National Research Council (IGAG-CNR), Via Marengo 2, 09123 Cagliari, Italy

**Keywords:** gold, catalysis, metal recovery, circular economy, WEEE, elemental sustainability

## Abstract

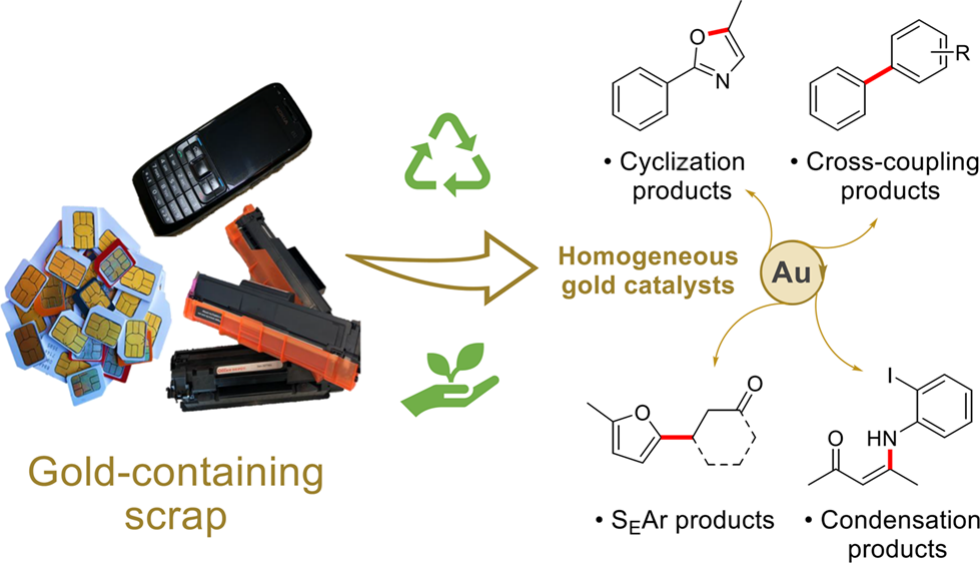

Despite the greater awareness of elemental sustainability and the benefits of the circular economy concept, much waste electrical and electronic equipment (WEEE) is still destined for landfill. Effective methods for valorizing this waste within our society are therefore imperative. In this contribution, two gold(III) complexes obtained as recovery products from WEEE and their anion metathesis products were investigated as homogenous catalysts. These four recovery products were successfully applied as catalysts for the cyclization of propargylic amides and the condensation of acetylacetone with *o*-iodoaniline. Impressive activity was also observed in the gold-catalyzed reaction between electron-rich arenes (2-methylfuran, 1,3-dimethoxybenzene, and azulene) and α,β-unsaturated carbonyl compounds (methyl vinyl ketone and cyclohexenone). These recovered compounds were also shown to be effective catalysts for the oxidative cross-coupling reaction of aryl silanes and arenes. When employed as Lewis acid catalysts for carbonyl-containing substrates, the WEEE-derived gold complexes could also be recovered at the end of the reaction and reused without loss in catalytic activity, enhancing still further the sustainability of the process. This is the first direct application in homogeneous catalysis of gold recovery products sourced from e-waste.

## Abstract 2

Gold complexes recovered from e-waste using a mild process are effective catalysts for a range of gold-catalyzed transformations.

## Introduction

Waste electrical and electronic equipment (WEEE) is one of the fastest growing waste streams in the world, and the majority of this material is currently still sent to landfill despite its precious metal content.^1^ The secondary raw material in this “urban mine” has the potential to yield far more gold (e.g., 50–700 g/ton from printed circuit boards)^2^ than that typically obtained from the same amount of primary gold mined ores or concentrates (1–10 g/ton), the production and processing of which rely on highly polluting and environmentally damaging processes.^1,3,4^ Along with these environmental factors, the unrelenting demand for gold and its limited availability in the earth’s crust lead to its high cost ($60 per gram, June 2022). This has led to the investigation of approaches that can valorize end-of-life WEEE using innovative, low-impact recovery processes,^5^ which are both economically and environmentally more sustainable than the established mining of gold from ore.

**Scheme 1:**
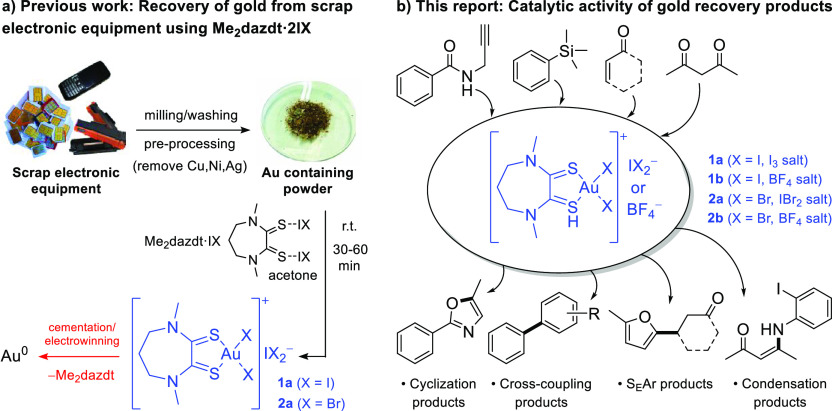
(a) Reaction Scheme Illustrating Previous Study on the Low-Impact Dissolution and Recovery of Gold from Waste Electronic Equipment and (b) Reaction Scheme Illustrating the Catalytic Activity of Gold Recovery Products **1a**, **1b**, **2a** and **2b** Derived from Waste Electronic Equipment Described in This Report

A leading example of such a process is the system patented by Deplano and co-workers in 2008,^6^ which is based on a selective, three-stage metal leaching and recovery approach from WEEE, in which gold is leached as the last, least reactive metal. This solvometallurgical method employs common organic solvents and environmentally friendly reagents under mild conditions. Using currently unrecycled waste as a feedstock, this method has been proposed as a “greener” alternative to the harmful and polluting processes traditionally used in gold mining and recycling. These traditional approaches typically require harsh conditions (i.e., pyrometallurgy) and reactants (cyanidation, aqua regia) and also produce harmful emissions.^5,7,8^ The final gold leaching step is achieved under gentle conditions typically using an acetone solution of the bis(diiodine) adduct of *N*,*N*-dimethylperhydrodiazepine-2,3-dithione (Me_2_dazdt·2I_2_) at room temperature. Under these patented conditions, gold dissolution occurs relatively rapidly (30–60 min), providing the complex [AuI_2_(Me_2_dazdt)]I_3_ (**1a**) as the leaching product (Scheme 1a). In addition to the recovery of **1a** from the process, the corresponding bis(iodine monobromide) adduct, Me_2_dazdt·2IBr, was also investigated and provided [AuBr_2_(Me_2_dazdt)]IBr_2_ (**2a**) in a high yield and under mild conditions (Scheme 1a).^9^ Almost quantitative recovery of the metal and the ligand from either **1a** or **2a** is subsequently achieved by cementation or electrowinning. However, further treatment to produce gold metal (process shown in red in Scheme 1a) increases the environmental and economic costs of the recovery process, requiring additional reagents, refining steps, and other energy-intensive processes. Despite the efficacy of this leaching method and the potential for the recovery of Me_2_dazdt, the cost of this dithiooxamide ligand is an additional impediment to its application in an industrial-scale recovery process to produce gold metal.

Direct application of compounds **1a** and **2a** would enable their valorization and thus create a new end-of-life application for gold-containing waste that is currently sent to landfill as well as provide additional economic incentive for the implementation of Deplano’s recovery process (Scheme 1a). The present study seeks to achieve exactly this by demonstrating that the recovery products [AuI_2_(Me_2_dazdt)]I_3_ (**1a**) and [AuBr_2_(Me_2_dazdt)]IBr_2_ (**2a**) can be used as effective homogeneous catalysts for a range of catalytic reactions, hence connecting an existing mild and effective recovery process with a major application of this metal (Scheme 1b).^9−11^ Inspired by the implementation of circular economy models,^12^ we have recently illustrated this approach in our own study through the use of palladium complexes recovered from spent three-way catalytic converters (TWCs) in catalysis.^13−16^ The complex [Pd(Me_2_dazdt)_2_]I_6_ is obtained as the palladium recovery product from spent TWCs through the action of solvometallurgical leaching using Me_2_dazdt·2I_2_.^13^ This molecular recovery product was subsequently used as a precursor for a nanostructured Pd-TiO_2_ photocatalyst used to produce hydrogen through the photoreforming of alcohols.^14^ This was followed by the direct use of [Pd(Me_2_dazdt)_2_]I_6_ as a catalyst for C–H oxidative functionalization reactions.^15^ In both cases, the performance of the recovered material was found to match or surpass that achieved by established palladium catalysts. These studies focusing on the area of sustainable palladium catalysis prompted us to investigate the catalytic activity of gold complexes obtained as leaching products from gold-containing waste.^17,18^

Herein, we report the application of [AuI_2_(Me_2_dazdt)]I_3_ (**1a**) and [AuBr_2_(Me_2_dazdt)]IBr_2_ (**2a**) in a range of catalytic reactions, as summarized in Scheme 1b. As part of these investigations, the tetrafluoroborate salts, [AuI_2_(Me_2_dazdt)]BF_4_ (**1b**) and [AuBr_2_(Me_2_dazdt)]BF_4_ (**2b**), were also synthesized via anion metathesis of **1a** and **2a**, respectively. This allowed an assessment of the influence of the metal-bound halides and counter anions on catalytic activity. The catalytic reactions explored in this study were chosen to reflect various facets of the reactivity of gold catalysts. They are representative examples of the growing field of homogeneous gold-catalyzed reactions, for which no other catalysts have yet been identified, or where gold enables catalysis under milder reaction conditions.^19−21^ Specifically, this study includes the cyclization of *N*-propargylbenzamides, condensation of acetylacetone and *o*-iodoaniline, addition reactions of electron-rich arenes to α,β-unsaturated carbonyl compounds, and oxidative C–C couplings of aryl silanes and arenes. The results obtained are discussed and compared with those reported in the literature for conventional gold(III) catalysts, which have been derived from mining. Finally, the charged nature of complexes **1a**, **1b**, **2a** and **2b** permitted their isolation from the organic reagents/products, which subsequently enabled their reuse as homogeneous catalysts. To the best of our knowledge, the study presented herein is the first direct catalytic application of molecular gold complexes from an established recovery process.

## Results and Discussion

### Preparation of the Gold Catalysts

**Scheme 2:**
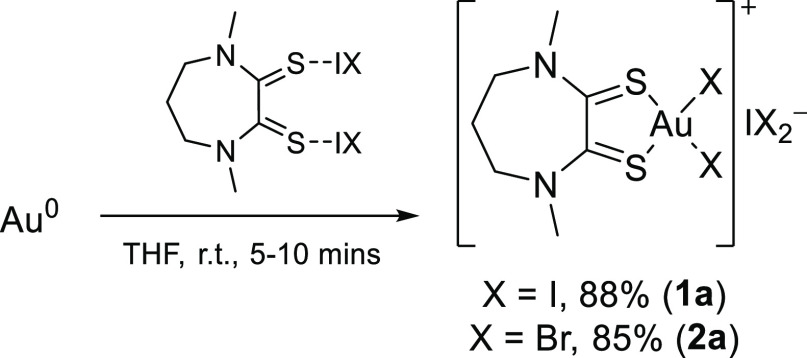
Gold Leaching in Solution Using Me_2_dazdt/I_2_ or Me_2_dazdt/IBr

Synthesis of the Me_2_dazdt ligand used in this study was carried out according to literature procedures.^10^ Dithiooxamide gold(III) complexes **1a** and **2a** employed in this study were synthesized both from actual scrap (SIM cards, Supporting Information Section 1.2) and gold metal powder, with the latter route providing a less time-consuming way to obtain gold complexes for the current investigation (Scheme 2).^6,11,22,23^ Characterization data for both gold complexes, obtained as black crystalline solids, were consistent with those reported in the literature. A preliminary indicative cost analysis (Supporting Information Section 1.6) shows that even unoptimized, small-scale production of catalyst **1a** leads to a significantly lower cost than commercial catalysts derived from environmentally damaging mining.

**Scheme 3:**
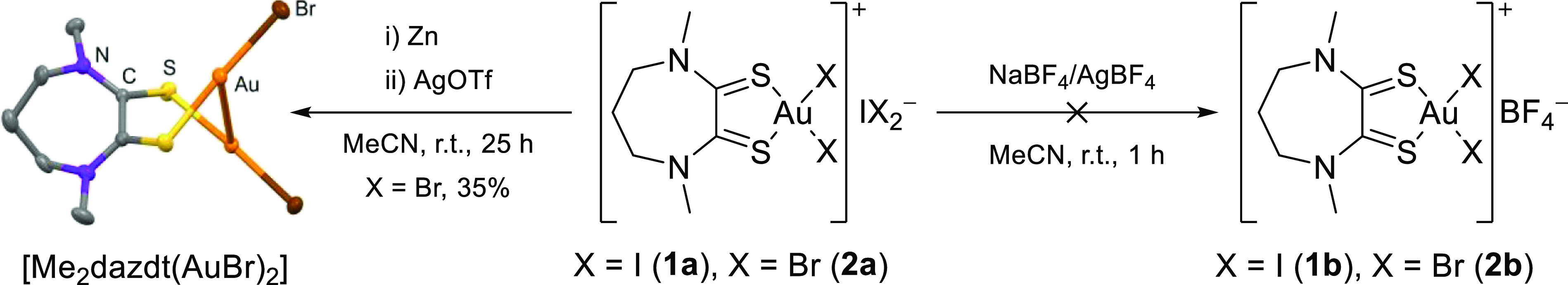
Unsuccessful Anion Metathesis of **1a** and **2a** with either NaBF_4_ or AgBF_4_ (on Right) and the Formation of a Neutral Bimetallic Au(I) Complex through Attempted Reduction and Bromide Metathesis of IBr_2_ (Left)

Initial attempts to synthesize the trihalide-free complexes, [AuI_2_(Me_2_dazdt)]BF_4_ (**1b**) and [AuBr_2_(Me_2_dazdt)]BF_4_ (**2b**), employing conventional halide anion metathesis techniques (NaBF_4_ and AgBF_4_) were unsuccessful (Scheme 3, right). In each case, the electrospray ionization (ESI) mass spectra of the resulting black powders revealed that the samples still contained significant amounts of trihalide anions.

Attempted reduction of IBr_2_ with zinc metal followed by bromide metathesis with AgOTf resulted in the formation of the free Me_2_dazdt ligand and a novel neutral bimetallic complex [Me_2_dazdt(AuBr)_2_] (Scheme 3, left), which was structurally characterized by single-crystal X-ray diffraction (Supporting Information Figures S9 and S10).

**Scheme 4:**
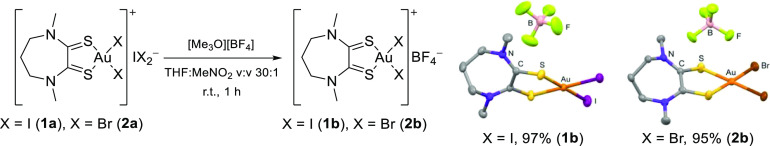
Successful Anion Metathesis with Trimethyloxonium Tetrafluoroborate To Yield **1b** and **2b** and Single-Crystal X-ray Molecular Structures Illustrated as Thermal Ellipsoids with 50% Probability

Successful metathesis of the trihalide anion was finally achieved using trimethyloxonium tetrafluoroborate (Meerwein’s salt) in a solvent mixture of THF and MeNO_2_ (v:v, 30:1), yielding [AuI_2_(Me_2_dazdt)]BF_4_ (**1b**) and [AuBr_2_(Me_2_dazdt)]BF_4_ (**2b**) cleanly after 1 h (Scheme 4). The ESI mass spectrometry analysis confirmed the absence of any trihalide anions in the black products obtained from the reaction. This was further confirmed by ^19^F nuclear magnetic resonance (NMR) analysis in which the BF_4_^–^ resonance was integrated against a known quantity of an internal standard (Supporting Information Figures S1 and S2). Finally, X-ray diffraction of single crystals isolated from the reaction of Meerwein’s salt with **1a** and **2a** confirmed the presence of BF_4_^–^ and provided structural data for **1b** and **2b** (Supporting Information Figures S7 and S8). Good agreement of elemental analysis data with the calculated values confirmed the formulation of the bulk material.

### Alkyne Activation Reactions

#### Cyclization of *N*-Propargylbenzamide

The activation of alkynes is one of the most common processes catalyzed by gold and so this was selected as the first reaction type to be investigated with the cationic gold(III) complexes derived from the recovery process. The gold-catalyzed synthesis of oxazoles is one of the most prominent transformations of propargylamides^24^ and one in which both gold(I) and gold(III) complexes have shown superior catalytic performance compared to the mercury(II) and palladium(II) complexes used previously.^25^ The use of tetrachloroauric acid as a reference catalyst led to almost full conversion of the starting material (**5**) to the desired product 4-methyl-2-phenyloxazole (**6**) (Table 1, entry 1). When the same reaction was repeated with the dithiooxamide gold complexes **1a**, **1b**, **2a** and **2b**, oxazole **6** was not detected (Table 1, entry 1). This lack of activity was initially ascribed to the strength of the metal–ligand bonds hindering the catalytic activity of the complexes. In order to aid ligand dissociation, two modifications to the procedure were explored in which the reaction mixture was heated (60 °C) and a silver salt additive (e.g., AgBF_4_) was introduced. However, this also proved unsuccessful (Table 1, entry 2).

**Table 1:**
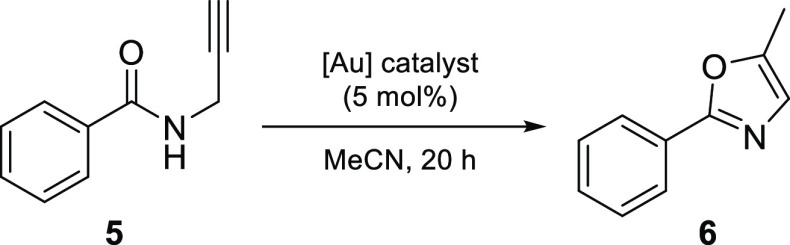
Results for the Gold(III)-Catalyzed Cyclization of *N*-Propargylbenzamide **5**

			yield of 6 (%)^a^				
entry	temperature (°C)	additive	HAuCl_4_	**1a**	**2a**	**1b**	**2b**
1	25	none	>95	0	0	0	0
2	60	AgBF_4_ (5 mol %)		trace	trace	0	0
3	60	pyridine-*N*-oxide (15 mol %)		82 ± 4	>95	91 ± 2	>95

a Yields are an average of three experiments and were determined using ^1^H NMR spectroscopy.

Strong hydrogen bond acceptors such as pyridine-*N*-oxide are known to improve the sluggish protodeauration stage,^26^ which has previously been identified as the rate-determining step in the cyclization of *N-*propargylbenzamide by cationic gold(I) catalysts.^27^ The use of the dithiooxamide gold complexes **1a**, **1b**, **2a** and **2b** in the presence of pyridine-*N*-oxide at 60 °C led to excellent yields of **6** (Table 1, entry 3). The bromide-containing complexes **2a** and **2b** displayed slightly improved activity compared to their iodide analogues (Table 1, entry 3).

Following the successful cyclization of terminal propargylic amide **5**, the study was extended to internal alkynes. To date, the cyclization of nonterminal propargylic amides such as **7** has only been explored with gold(I) precatalysts, yielding a mixture of 5-*exo* (**8**) and 6-*endo* (**9**) products.^28,29^ The conditions used for the successful transformation of the terminal propargylic amide **5** (Table 1, entry 3) were employed initially for the internal alkyne **7**, as shown in Table 2.

**Table 2:**
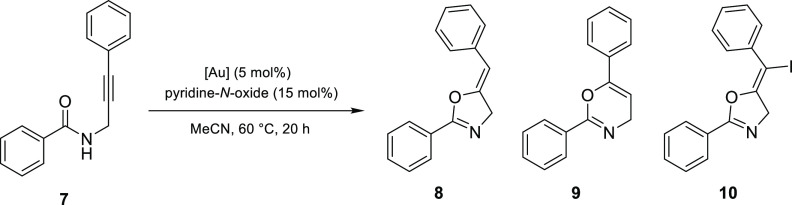
Gold(III)-Catalyzed Cyclization of the Internal Alkyne *N*-Propargylbenzamide (**7**)

entry	catalyst	product	yield (%)^a^
1	**1a**	**10**	8
2	**2a**	**10**	∼1
3	**1b**	**8**	72
4	**2b**	**8**	81
5	AuCl_3_	**8** and **9**	65:30

a Yields are reported as isolated yields following purification by flash column chromatography. Where only one product is shown, only trace quantities of the other products (**8**–**10**) could be identified in the crude mixture by ^1^H NMR analysis.

Surprisingly, catalysis by the recovered gold complex [AuI_2_(Me_2_dazdt)]I_3_ (**1a**) yielded neither of the expected cyclization products (**8** or **9**). Instead, vinyl iodide **10** was isolated in 8% yield along with the gold metal. The structure of **10** was confirmed by halogen-lithium exchange (^*n*^BuLi) with a subsequent proton quench (H_2_O) yielding alkene **8**, which displayed characteristic allylic alkene coupling (^4^*J*_HH_ = 2.4 Hz) between the alkene triplet (5.69 ppm) and the methylene doublet (4.86 ppm). Use of [AuBr_2_(Me_2_dazdt)]IBr_2_ (**2a**) resulted in the formation of traces (∼1% from ^1^H NMR analysis of the reaction mixture) of vinyl iodide **10**. Previous work by both Hashmi and Čikotiene illustrated that the cyclization of nonterminal propargylic amides can be promoted by electrophilic “I^+^” sources (e.g., *N*-iodosuccinimide) to yield vinyl iodide **10**,^29,30^ suggesting that the central “I^+^” in the trihalide counteranion was the iodine source for the formation of **10**. This was confirmed when identical reaction conditions employing stoichiometric NaI_3_ yielded an intractable mixture of 5-*exo* (**10**) and 6-*endo* vinyl iodide products (combined yield of 12%). The difference in 5-*exo*/6-*endo* regioselectivity observed between **1a** and NaI_3_ suggested that the formation of **10** proceeded via gold-catalyzed cyclization and subsequent deauration with “I^+^.” This result indicated that the tetrafluoroborate salts [AuI_2_(Me_2_dazdt)]BF_4_ (**1b**) and [AuBr_2_(Me_2_dazdt)]BF_4_ (**2b**) might be more suitable for catalyzing the desired cyclization to yield **8**. Indeed, complexes **1b** and **2b** promoted cyclization to provide the oxazoline product **8** in yields of 72 and 81%, respectively, with only trace (<1%) quantities of oxazine **9** observed in the crude reaction mixture. Despite resulting in slightly lower overall conversions, the recovered complexes **1b** and **2b** provided much greater control compared to AuCl_3_, allowing the 5-*exo* product **8** to be formed selectively.

#### Carbonyl Activation Reactions

The activation of carbonyl groups toward nucleophilic attack is a less common aspect of gold-mediated reactivity but is observed when gold(III) complexes possess a harder, oxophilic Lewis acidic character. In a key study by Arcadi et al.,^31^ gold(III)-catalyzed condensation of 1,3-dicarbonyls with amines led to the formation of β-enaminones. This important class of synthetic intermediates is used for the assembly of various heterocycles, previously accessible via the azeotropic removal of water in refluxing aromatic solvents,^31^ which is unsuitable for more sensitive substrates. Using gold catalysis, however, enaminones can now be synthesized under much milder conditions (e.g., in ethanol at room temperature).

Conditions identical to those described by Arcadi et al. (2.5 mol %, ethanol, room temperature) were utilized in this study using recovered gold complexes. The results for the condensation reaction between acetylacetone and *o*-iodoaniline to form the corresponding β-enaminone (**11**) are summarized in Table 3. Under these conditions, only trace amounts of the product were formed in the absence of any catalyst (Table 3). However, in the presence of 2.5 mol % of the reference catalyst (HAuCl_4_), the observed yield was higher than that reported by Arcadi et al. (60%, using NaAuCl_4_), an observation which can be attributed to the reaction also being favored by Brønsted acids.

**Table 3:**
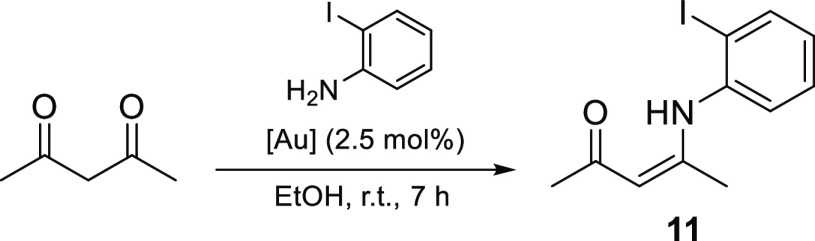
Gold(III)-Catalyzed Condensation of Acetylacetone and *o*-Iodoaniline

	yield (%)^a^				
no catalyst	HAuCl_4_	**1a**	**2a**	**1b**	**2b**
trace	74 ± 0	62 ± 6	64 ± 4	52 ± 4	65 ± 3

a Yields calculated as conversions using ^1^H NMR spectroscopy are based on an average of three experiments.

Analysis of the results shows that gold(III) catalysts **1a**, **1b**, **2a** and **2b** all display a similar activity in the formation of **11**, regardless of the nature of the halide ligand or counteranion (Table 3). Furthermore, the catalysts could be recovered and reused following concentration of the reaction mixture and subsequent extraction of the organic components by trituration with hexane. The resulting black solids, effectively free from any organic contaminants (as indicated by ^1^H NMR analysis), could be reused as homogeneous catalysts with no discernible loss in catalytic activity even after eight reuse cycles. This recycling strategy was also successful using diethyl ether, chloroform, and dichloromethane, while the use of polar and coordinating solvents (acetonitrile, tetrahydrofuran, and acetone) resulted in coextraction of both the organic and gold compounds. These results suggest that this simple catalyst recycling approach could be applied to a range of substrates with differing solubilities in organic solvents.

In 2003, Dyker and co-workers pioneered the use of AuCl_3_ to catalyze the electrophilic aromatic substitution of arenes and heteroarenes with methyl vinyl ketone (MVK), affording alkylated arenes in 50–90% yield.^32^ The same reaction conditions were employed in this study to explore the effectiveness of dithiooxamide gold(III) recovery complexes **1a**, **1b**, **2a** and **2b** (Table 4) in this transformation.

**Table 4:**
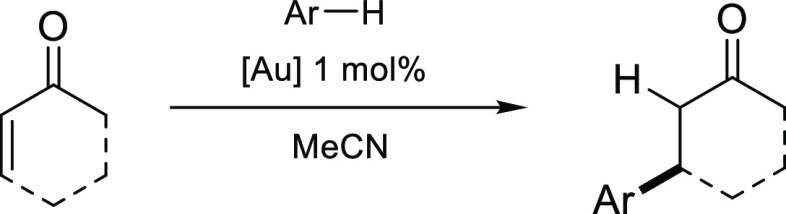

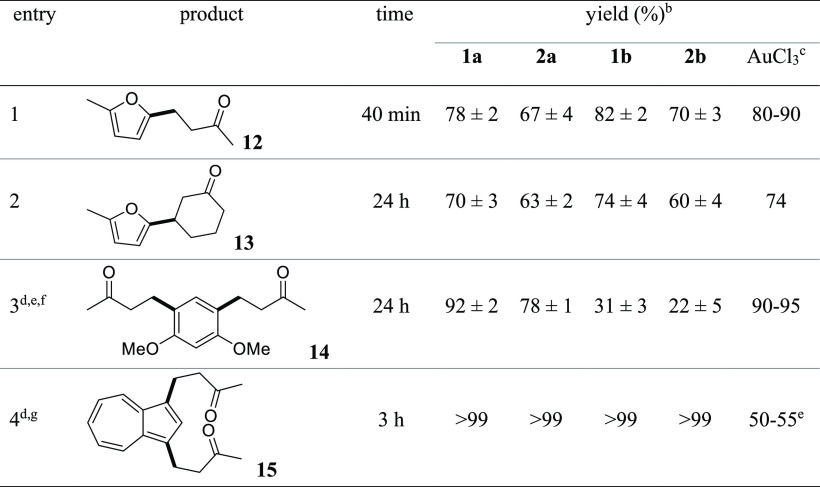
Gold(III)-Catalyzed Addition of Nucleophilic Arenes to Unsaturated Carbonyl Compounds^a^

a Reactions were carried out using 1 mol % catalyst loading, [arene] = 0.25 M in MeCN, and 1 equivalent of MVK at room temperature, unless otherwise stated.

b Yields are of the isolated pure product and are the average of three runs (this work only).

c AuCl_3_ yields taken from the study of Dyker et al.^32^

d Reactions were performed at 60 °C.

e 4 equivalents of MVK are used.

f Yields are determined based on the ^1^H NMR analysis and are the average of three experiments.

g 2 equivalents of MVK are used.^32^

The scope of the reaction was expanded to explore the versatility of gold complexes **1a**, **1b**, **2a** and **2b** in C–C bond formation with different substrates (Table 4, entries 2–4). For the reactions with 2-methylfuran and 1,3-dimethoxybenzene, gold complexes **1a**, **1b**, **2a** and **2b** produced similar results to those obtained previously using AuCl_3_ (Table 4, entries 1 and 3). Excellent reactivity was also observed when electrophilic cyclohexenone was used in the place of MVK (entry 2). In general, the iodide complexes, [AuI_2_(Me_2_dazdt)]I_3_ (**1a**) and [AuI_2_(Me_2_dazdt)]BF_4_ (**1b**), performed better than their bromide analogues, [AuBr_2_(Me_2_dazdt)]IBr_2_ (**2a**) and [AuBr_2_(Me_2_dazdt)]BF_4_ (**2b**). Little difference in catalytic activity was observed between the trihalide and tetrafluoroborate complexes in the reactions of 2-methylfuran (entry 1); however, a significant difference in activity was observed between the trihalide salts (**1a**/**2a**) and tetrafluoroborate salts (**1b**/**2b**) on the addition of MVK to the less nucleophilic 1,3-dimethoxybenzene (entry 3). Interestingly, quantitative yields were observed for the addition of azulene to MVK (entry 4), representing a significant improvement over AuCl_3_. As for the condensation between acetylacetone and *o*-iodoaniline (Table 3), it was found that gold complexes **1a**, **1b**, **2a** and **2b** could be isolated and reused following concentration and subsequent extraction of the organic components by trituration with hexane, with no discernible loss in catalytic activity for the reactions presented in entries 1, 2, and 4 even after five reuse cycles.

#### Oxidative Coupling of Arenes with Aryl Silanes

In recent years, gold-catalyzed cross-coupling reactions have emerged as a powerful technique for the synthesis of biaryl compounds. In a recent example, it was shown that a number of aryl silanes and electron-rich arenes could be coupled in the presence of catalytic amounts of the gold(I) complex [(Ph_3_P)AuOTs] (OTs = OSO_2_C_6_H_4_Me-4).^33^ In a subsequent report, the same group reported that AuX_3_ (X = halide, OMe, and camphorsulfonate), and not (Ph_3_P)AuX, was actually the active catalyst species, and that simple AuX_3_ precatalysts (e.g., [AuBr_3_(tht)], tht = tetrahydrothiophene) provided improved performance compared to [(Ph_3_P)AuOTs].^34^ It was therefore envisaged that the recovered gold(III) complexes reported here could deliver a similar performance to that reported for [AuBr_3_(tht)].

The cross-coupling reaction to yield **16** was chosen as a representative example of the oxidative coupling of arenes with aryl silanes (Table 5). An initial attempt using literature conditions^33^ led to a poor yield of the desired product **16** (Table 5, entry 1). An increase in the catalyst loading from 1 to 2 mol % resulted in a slight improvement of the cross-coupling yield; however, the amount of unwanted homocoupling product **17** also increased (Table 5, entry 2). Similarly, an increase in the temperature (Table 5, entry 3) or addition of AgSbF_6_ (Table 5, entry 4) improved the yield of the desired product **16** but also enhanced the homocoupling side reaction. It should also be noted that other unidentified methoxy-containing side products were also formed in the presence of the silver salt, as indicated by ^1^H NMR spectroscopy. Due to these competing factors, a design of experiments (DoE) approach was utilized to optimize the synthesis of **16**. The influence of catalyst loading (1–3 mol %), substrate concentration (0.1–0.25 M), arene equivalence (1–1.5), silver salt loading (0–6 mol %), and temperature (30–60 °C; a lower limit of 30 °C was chosen to eliminate any variations in ambient temperature) on both the desired cross-coupling and unwanted aryl silane homocoupling was explored. The final optimized conditions obtained from the DoE screening are summarized in Table 5, entry 5.

**Table 5:**
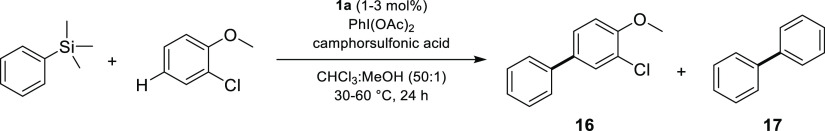
Optimization of the Gold(III)-Catalyzed Oxidative Coupling of Arenes and Aryl Silanes Using [AuI_2_(Me_2_dazdt)]I_3_ (**1a**) as the Catalyst

	conditions				yield (%)^a^	
entry	catalyst loading (mol %)	concentratio*n* (M)	arene equivalence	temperature (°C)	**16**	**17**
1	1	0.10	1	r.t.	15	18
2	2	0.10	1	r.t.	20	25
3	1	0.10	1	60 °C	40	25
4^b^	1	0.10	1	60 °C	45	28
**5**^c^	**1**	**0.23**	**1.5**	**30 °C**	**73**	**6**

a Yields are determined by HPLC.

b Reaction is performed in the presence of AgSbF_6_ (6 mol %).

c Final optimized conditions are extracted from a design of experiment optimization.

The DoE optimization revealed that arene equivalence was the most significant variable in determining both cross-coupling and homocoupling yields, with an excess of arene (1.5 equivalents) providing the highest cross-coupling and lowest homocoupling yields (Supporting Information, Figure S4). The lowest temperature screened was 30 °C and this was found to provide optimal cross-coupling yields, while a corresponding increase in aryl silane homocoupling was observed at elevated temperatures (Supporting Information, Figure S6) alongside unidentified methoxy-containing side products (as indicated by ^1^H NMR analysis). The addition of a silver salt led to only a small increase in the yield of the cross-coupling product and was thus omitted from the final optimized conditions. Interestingly, the catalyst loading was also found to have no significant influence on the yield of the desired cross-coupling product, which led to an optimized catalyst loading of 1 mol % (Table 5, entry 5).

After establishing the optimized conditions for the oxidative coupling of aryl silanes with arenes using the recovered gold complexes, the substrate scope was explored. Given that similar yields were obtained for both the trihalide (**1a** and **2a**) and tetrafluoroborate (**1b** and **2b**) salts, only directly recovered complexes [AuI_2_(Me_2_dazdt)]I_3_ (**1a**) and [AuBr_2_(Me_2_dazdt)]IBr_2_ (**2a**) were employed. In general, similar reactivity patterns were observed to those reported in the literature,^33^ with electron-rich arenes proving to be more reactive (Table 6, entries 1–2 and 5) than electron-deficient arenes (Table 6, entry 6). Overall, comparable yields were obtained using the gold recovery complexes compared to the values reported using [(Ph_3_P)AuOTs],^33^ while the regioselectivities for the less electron-rich arenes (Table 6, entries 3 and 4) were an improvement on those obtained using the literature system.

**Table 6:**
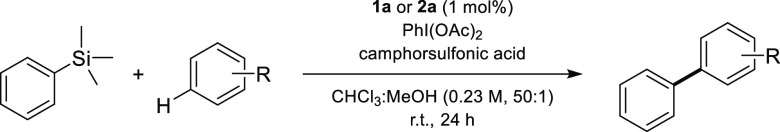

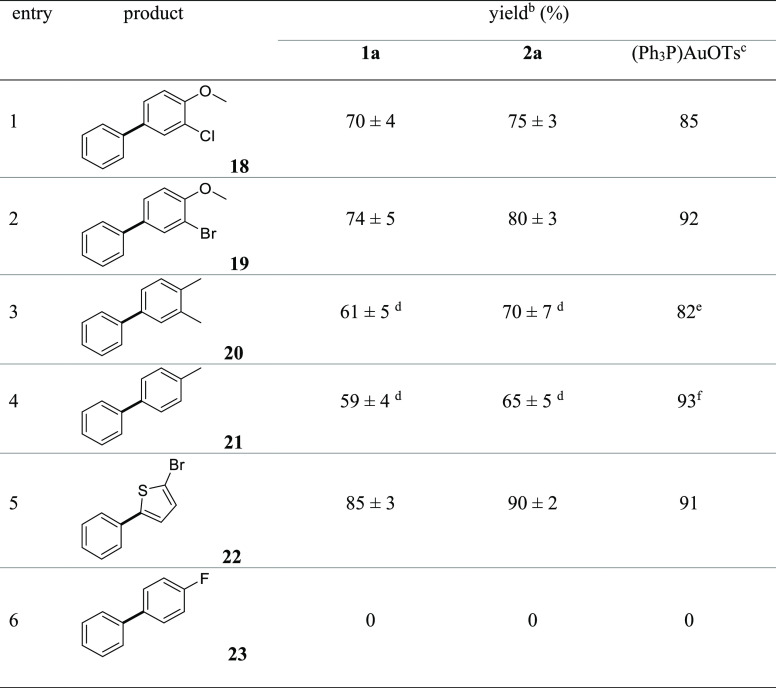
Oxidative Coupling of Arenes and Aryl Silanes Catalyzed by [AuI_2_(Me_2_dazdt)]I_3_ (**1a**) and [AuBr_2_(Me_2_dazdt)]IBr_2_ (**2a**)^a^

a Reactions were carried out using conditions optimized using DoE: [Au] = 1 mol %; [arene] = 0.345 M, and [aryl silane] = 0.23 M; r.t.

b Reported yields are of the purified isolated product after flash column chromatography and are the average of three experiments.

c Yields for reactions catalyzed by (Ph_3_P)AuOTs (longer reaction time of 30 h) are taken from the literature.^33^

d Product is isolated as a single regioisomer.

e 6.6:1.0 ratio of *meta*/*ortho* isomers.

f 4.1:1 ratio of *para*/*ortho* isomers.

## Conclusions

This study has revealed that the dithiooxamide gold(III) salts, [AuI_2_(Me_2_dazdt)]I_3_ (**1a**) and [AuBr_2_(Me_2_dazdt)]IBr_2_ (**2a**), obtained from WEEE as direct recovery products from the mild and high-yield leaching process developed by Deplano and co-workers are effective homogeneous gold catalysts. These findings pave the way for valorization of these recovery products as an alternative to return the gold content to its elemental form. This contribution also illustrates the potential for improving the sustainability of gold catalysis through the recovery of this metal from e-waste, as illustrated by the pathway from end-of-life SIM cards to active catalyst (Supporting Information, Section 1.2). Two additional trihalide-free recovery complexes, [AuI_2_(Me_2_dazdt)]BF_4_ (**1b**) and [AuBr_2_(Me_2_dazdt)]BF_4_ (**2b**), were also prepared via a novel anion metathesis strategy employing trimethyloxonium tetrafluoroborate. When compared to benchmark catalysts, the recovery products showed very good catalytic activity in addition reactions between electron-rich arenes and carbonyl compounds, suggesting that the reactivity of compounds **1a**, **1b**, **2a** and **2b** resembles that of oxophilic Lewis acids. It was also demonstrated that these gold(III) recovery complexes could be isolated and reused multiple times following the reaction between arenes and carbonyl compounds, further enhancing their utility as a sustainable alternative to traditional homogeneous gold catalysts. Finally, good activity was observed for the gold(III) recovery products **1a** and **2a** in the oxidative cross-coupling reactions of a number of arenes with aryl silanes. These are the first examples of the direct application in homogeneous catalysis of gold recovery products sourced from waste material such as WEEE. They demonstrate that the existing cationic gold(I) and neutral gold(III) catalysts derived from environmentally damaging gold mining can be replaced with more sustainable and cheaper alternatives recovered from millions of tonnes of e-waste currently sent to landfill each year.

## Supporting Information Available

Synthetic and catalytic procedures and characterization data with selected NMR spectra and analytical data including crystallographic details for the structures of compounds **1b**, **2b**, and [Me_2_dazdt(AuBr)_2_] (PDF)
