# Supplementary material for: Homogeneous Gold Catalysis Using Complexes Recovered from Waste Electronic Equipment
Source: ACS Sustain Chem Eng. 2022 Nov 17;10(48):15726–34. doi: 10.1021/acssuschemeng.2c04092 (PMC9727779; doi:10.1021/acssuschemeng.2c04092)
Supplement: Supplementary file 1 — sc2c04092_si_001.pdf [file sc2c04092_si_001.pdf]

Homogeneous Gold Catalysis Using Complexes Recovered from Waste Electronic Equipment

Sean McCarthy,[a] Oriane Desaunay, Alvin Lee Wei Jie, Maximilian Hassatzky, Andrew J. P. White, Paola Deplano, D. Christopher Braddock[*] Angela Serpe[*] and James D. E. T. Wilton-Ely[*]

Electronic Supplementary Information

1. Experimental

1.1. General experimental S2

1.2. Synthesis of gold complexes **1a-2b** S2

1.3. Synthesis of propargylic amides S8

1.4. Catalytic testing of gold recovery products **1a-2b** S9

1.4.1. Cyclization of *N*-propargylic amides S9

1.4.2. Condensation of *o*-iodoaniline and acetylacetone S11

1.4.3. Addition of nucleophilic arenes to α,β-unsaturated carbonyl compounds S12

1.4.4. Oxidative coupling of aryl silanes and arenes S15

1.5. X-Ray crystallographic information S20

1.6. Preliminary indicative cost analysis S29

1.7. References S32

2. NMR Spectra

2.1. ^{1}H NMR Spectra S33

2.2. $^{13}C\{^{1}H\}$ NMR Spectra S51

Number of figures: 36
Number of schemes: 18
Number of tables: 11

1.0. Experimental

1.1. General experimental

All reagents used in this project were purchased from commercial suppliers and used without further purification. THF is used as an abbreviation for tetrahydrofuran throughout. The reactions described were performed under air unless otherwise indicated. All stirrer bars and glassware used to perform catalysis were pre-washed with *aqua regia*. Crude products purified by column chromatography were passed through silica gel (pore size: 60 Å, 230-400 μm mesh size, 40-63 μm particle size). Thin-layer chromatography plates were visualized by exposure to ultraviolet light. Samples analyzed by mass spectrometry were ionized by electrospray ionization and recorded on a Waters Xevo TQ-MS/Acquity UPLC. ^{1}H, ^{19}F and ^{13}C NMR spectra were recorded on a Bruker Avance III 400 MHz spectrometer at room temperature and were referenced to the residual proton signal of the NMR solvent. Chemical shifts (δ) are reported in parts per million (ppm) and coupling constants (J) are given in Hz. IR spectra were recorded on an Agilent Cary 630 FTIR. Unless otherwise stated, provided analytical data are consistent with data found in the literature. HPLC analyses was performed on a PerkinElmer Series 200 HPLC fitted with a Supercosil LC18 (25 cm x 4.6 mm x 5 μm) and a Series 200 EP Diode Array UV-Vis detector measuring at 254 and 238 nm, and the data analyzed in TotalChrom. Design of experiment optimization was conducted in JMP pro 16.

1.2. Synthesis of gold complexes 1a-2b

***N,N′*-dimethylperhydrodiazepine-2,3-dione**

Dimethyloxalate (1.18 g, 10.0 mmol, 1.00 equiv.) was dissolved in toluene (20 mL) with stirring. *N,N′*- dimethylpropane-1,3-diamine (1.25 mL, 10.0 mL, 1.00 equiv.) was added to the stirring mixture and heated under reflux for 2 h. After cooling, the solvent was removed under reduced pressure to give a white powder crude product. Recrystallisation with hot iPrOH/ CH2Cl2 afforded *N,N′*- dimethylperhydrodiazepine-2,3-dione (1.28 g, 82%) as a white solid. IR (ATR): 2928, 2942, 1645 (C=O), 1608, 1498, 1459, 1442, 1426, 1404, 1263, 1193, 1094, 905, 864, 690 cm^{-1}; ^{1}H NMR (400 MHz, Chloroform-*d*) δ 3.44 (t, J = 6.4 Hz, 4H), 3.08 (s, 6H), 2.02 (p, J = 6.3 Hz, 2H) ppm.

Me_2dazdt (*1,4-dimethyl-1,4-diazepane-2,3-dithione*)

Me_2dazdt (*N,N'-dimethyl-perhydrodiazepine-2,3-dithione*) was prepared using literature conditions and the characterization of the obtained product was consistent with literature data.[S1]

N,N'-dimethylperhydrodiazepine-2,3-dione (0.780 g, 5.00 mmol, 1.00 equiv.) and Lawesson's reagent (3.04 g, 7.50 mmol, 1.50 equiv.) were dissolved in toluene (30 mL) and stirred under reflux for 2 h. After cooling, the solvent was removed *in vacuo* and the crude residue was purified by silica gel column chromatography (R_f = 0.26 [DCM]) yielding the title compound (706 mg, 75%). IR (ATR): 2922, 2855, 1491, 1435, 1379, 1260, 1185, 1111, 1081, 1025, 973 cm^{-1}. ^{1}H NMR (Chloroform-*d*): δ 3.77 (ddd, J = 14.8, 9.6, 7.9 Hz, 2H), 3.58–3.53 (m, 2H), 3.52 (s, 6H), 2.27–2.16 (m, 2H) ppm. HRMS (ESI) *m/z* $[M+H]^+$ calcd. for $C_7H_{13}N_2S_2$: 189.0521, found: 189.0520.

$[AuI_2(Me_2dazdt)]I_3$ (1a)

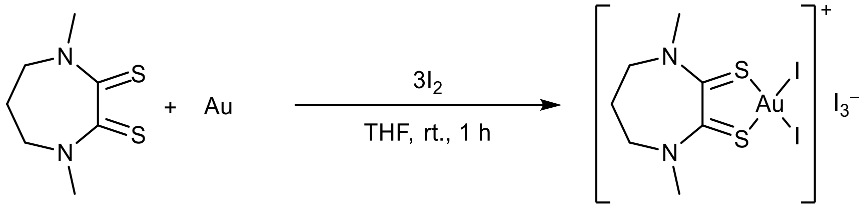

1a

Scheme S1. Gold leaching in solution using Me_2dazdt/I_2.

According to a modified literature procedure,[S2] Me_2dazdt (92 mg, 0.49 mmol), Au powder (96 mg, 0.49 mmol) and I_2 (372 mg, 1.46 mmol) were suspended in THF (70 mL) and stirred at room temperature for 1 h. The product was crystallized from the black solution by vapor phase diffusion with Et_2O. The desired product was isolated as reflective black crystals (437 mg, 88%). Characterization was consistent with literature data. IR (ATR): 3101, 2996, 2948, 2910, 2853, 2778, 2672, 1675, 1646, 1613, 1549, 1451, 1434, 1397, 1353, 1341, 1329, 1295, 1280, 1257, 1181, 1097, 1069, 1034, 1022, 953, 838, 822, 745, 690 cm^{-1}. ^{1}H NMR (400 MHz, Acetone-d_6): δ 3.79–3.69 (m, 2H), 3.68–3.57 (m, 2H), 3.47 (s, 6H), 2.38–2.29 (m, 2H) ppm.

$[AuBr_2(Me_2dazdt)]IBr_2$ (2a)

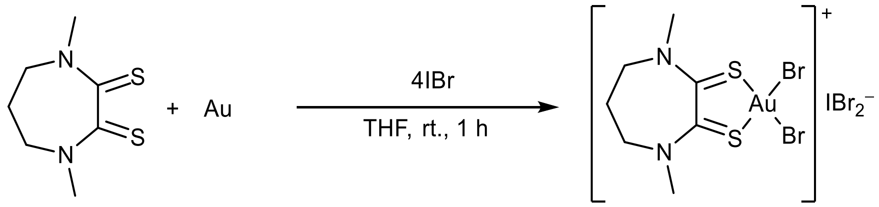

2a

Scheme S2. Gold leaching in solution using Me_2dazdt/IBr.

According to a modified literature procedure,[S3] Me_2dazdt (75 mg, 0.40 mmols), Au powder (80 mg, 0.40 mmols) and IBr (331 mg, 1.60 mmols) were suspended in THF (70 mL) and stirred at room temperature for 2 h. The product was crystallized from the black solution by vapor phase diffusion with Et_2O. The desired product was isolated as reflective black crystals (283 mg, 85%). IR (ATR): 2912, 1555, 1451, 1432, 1389, 1266, 1110, 1069, 954, 742, 605 cm^{-1}. ^1H NMR (400 MHz, Acetone-d_6): δ 3.79 (s, 4H), 3.57 (s, 6H), 2.43 (s, 2H) ppm.

$[AuI_2(Me_2dazdt)]I_3$ (1a) and $[AuBr_2(Me_2dazdt)]IBr_2$ (2a) from scrap SIM cards

The complete procedure for metals recovery reported by Deplano *et al.*, was followed in order to demonstrate that **1a** and **2a** can be obtained from real scrap subscriber identity module (SIM) cards:[S4]

Scrap SIM cards (~17 g) were initially ground to a coarse powder. The scrap material was then stirred in refluxing THF (50 mL). After 24 h, the white liquid (indicating the dispersion of the white plastic support) was decanted, and the resulting coarse powder isolated and washed with hot THF (2 x 10 mL). The coarse powder was suspended in a stirred solution of aqueous ammonia (32%, 10 mL) and ammonium sulphate (2.2 g). To the suspension was then added aqueous H_2O_2 (30 %, 3 mL) dropwise and the solution stirred at room temperature. After 4 h, the deep-blue solution (indicating the oxidation and dissolution of copper metal to form $[Cu(NH_3)_4]SO_4$) was decanted and the residue washed with distilled H_2O (2 x 10 mL). The resulting coarse powder was then suspended in a hot (80 °C) stirred solution of aqueous HCl (2.4 M, 25 mL). After 24 h, the green solution (indicating the oxidation and dissolution of Ni metal to form $NiCl_2$) was decanted and the coarse powder washed with distilled H_2O (2 x 10 mL). Finally, the course powder was suspended in a stirred solution of Me_2dazdt (92 mg, 0.53 mmol) and I_2 (372 mg, 1.46 mmol) or IBr (404 mg, 1.95 mmol) added in THF (80 mL). After 24, the black liquid was filtered and the filtrate concentrated to ~30 mL. The product was crystallized from the black liquid by vapor diffusion with Et_2O (using the vial within a vial method). Both **1a** (130 mg) and **2a** (105 mg) were isolated as reflective black crystals. Characterization data for these compounds were consistent with those reported in the literature and also with **1a** and **2a** synthesized from gold metal powder in this study. No difference in catalytic activity was observed between the complexes isolated from scrap SIM cards and the complexes isolated from gold powder.

[AuI$_2$(Me$_2$dazdt)]BF$_4$ (1b)

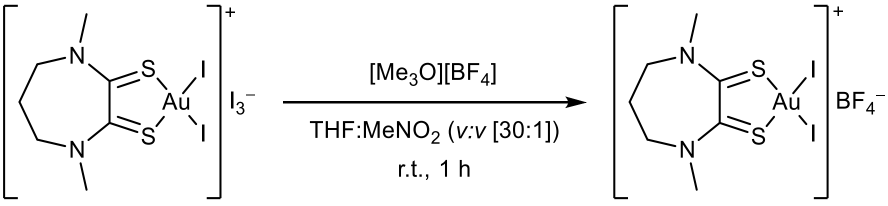

Scheme S3. Triiodide anion metathesis yielding [AuI$_2$(Me$_2$dazdt)]BF$_4$.

[AuI$_2$(Me$_2$dazdt)]I$_3$ (**1a**) (150 mg, 0.147 mmol) was dissolved in THF (120 mL). Trimethyloxonium tetrafluoroborate (22 mg, 0.15 mmol) was dissolved in MeNO$_2$ (4 mL) and the solution transferred to the THF solution in one portion. The reaction mixture was stirred for 1 h, concentrated *in vacuo* and the crude material recrystallized in MeCN by vapor phase diffusion with CHCl$_3$, yielding the title compound as reflective black crystals suitable for single crystal XRD (104 mg, 97%). IR (ATR): 1554, 1437, 1399, 1259, 1047, 1029, 948, 822, 746, 605, 519 cm^{-1}. ^{1}H NMR (400 MHz, Acetonitrile-*d*$_3$): δ 3.95 (t, J = 6.9 Hz, 4H), 3.72 (s, 6H), 2.65 (quin, J = 6.9 Hz, 2H) ppm. ^{19}F NMR: δ 151.7 (0.20F), 151.8 (0.80F) ppm. ^{13}C{^{1}H} NMR data are not reported due to the poor solubility of **1b** in common deuterated solvents including acetonitrile-*d*$_3$. Calculated for $C_7H_{12}AuBF_4I_2N_2S_2$ (Mw = 725.89): C 11.6, H 1.7, N 3.9 %. Found: C 11.7, H 1.6, N 3.5 %.

The purity of the BF_4^- salt was confirmed by ^{19}F NMR analysis of an MeCN-*d*$_3$ solution of **1b** (10 mg, 0.014 mmol) and fluorobenzene as an internal standard (5.2 µL, 0.055 mmol, 4 equivalents) (Figure S1).

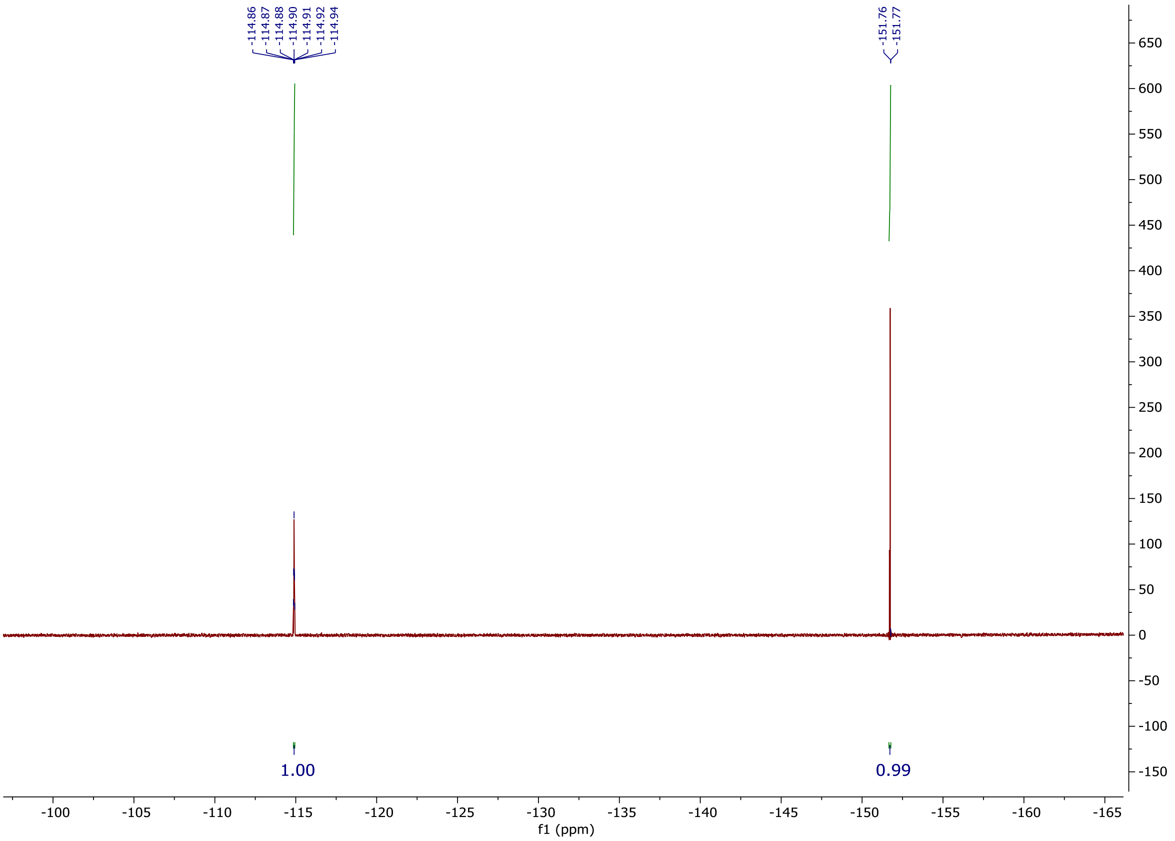

Figure S1. ^{19}F NMR spectrum of 4:1 equivalents of **1b** and fluorobenzene respectively illustrating complete conversion of $[AuI_2(Me_2dazdt)]I_3$ to $[AuI_2(Me_2dazdt)]BF_4$.

$[AuBr_2(Me_2dazdt)]BF_4$ (2b)

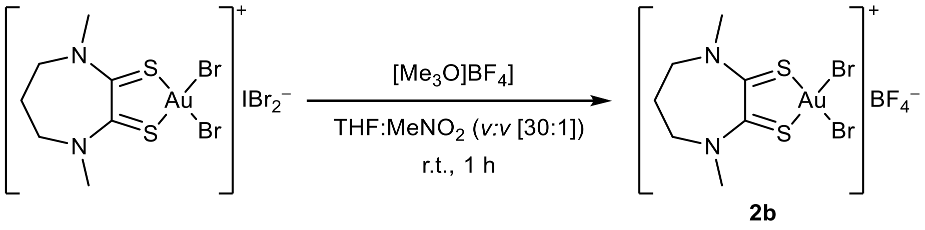

Scheme S4. Iodine dibromide anion metathesis yielding $[AuBr_2(Me_2dazdt)]BF_4$.

$[AuBr_2(Me_2dazdt)]IBr_2$ (**2a**) (55 mg, 0.074 mmol) was dissolved in THF (60 mL). Trimethyloxonium tetrafluoroborate (11 mg, 0.074 mmol) was dissolved in $MeNO_2$ (2 mL) and the solution transferred to the THF solution in one portion. The reaction mixture was stirred for 1 h, concentrated *in vacuo* and the crude material recrystallized in MeCN by vapor phase diffusion with $CHCl_3$, yielding the title compound as reflective black crystals suitable for single crystal XRD (47 mg, 95%) IR (ATR): 1742, 1562, 1437, 1399, 1359, 1262, 1230, 1018, 951, 824, 745, 603, 518, 413 cm^{-1}. ^{1}H NMR (400 MHz, Acetonitrile-d_3) δ 4.01 (t, J = 6.9 Hz, 4H), 3.76 (s, 6H), 2.67 (quin, J = 6.9 Hz, 2H) ppm. ^{19}F NMR: δ 151.7 (0.20F), 151.7 (0.80F) ppm.

$^{13}C\{^1H\}$ NMR data are not reported due to the poor solubility of **2b** in common deuterated solvents including acetonitrile-*d*3. Calculated for $C_7H_{12}AuBF_4I_2N_2S_2$ (Mw = 631.89): C 13.3, H 1.9, N 4.4 %. Found: C 12.8, H 1.7, N 4.0 %.

The purity of the BF_4^- salt was confirmed by ^{19}F NMR analysis of an MeCN-*d*3 solution of **2b** (4.3 mg, 0.0068 mmol) and fluorobenzene as an internal standard (2.6 μL, 0.027 mmol, 4 equivalents) (Figure S2).

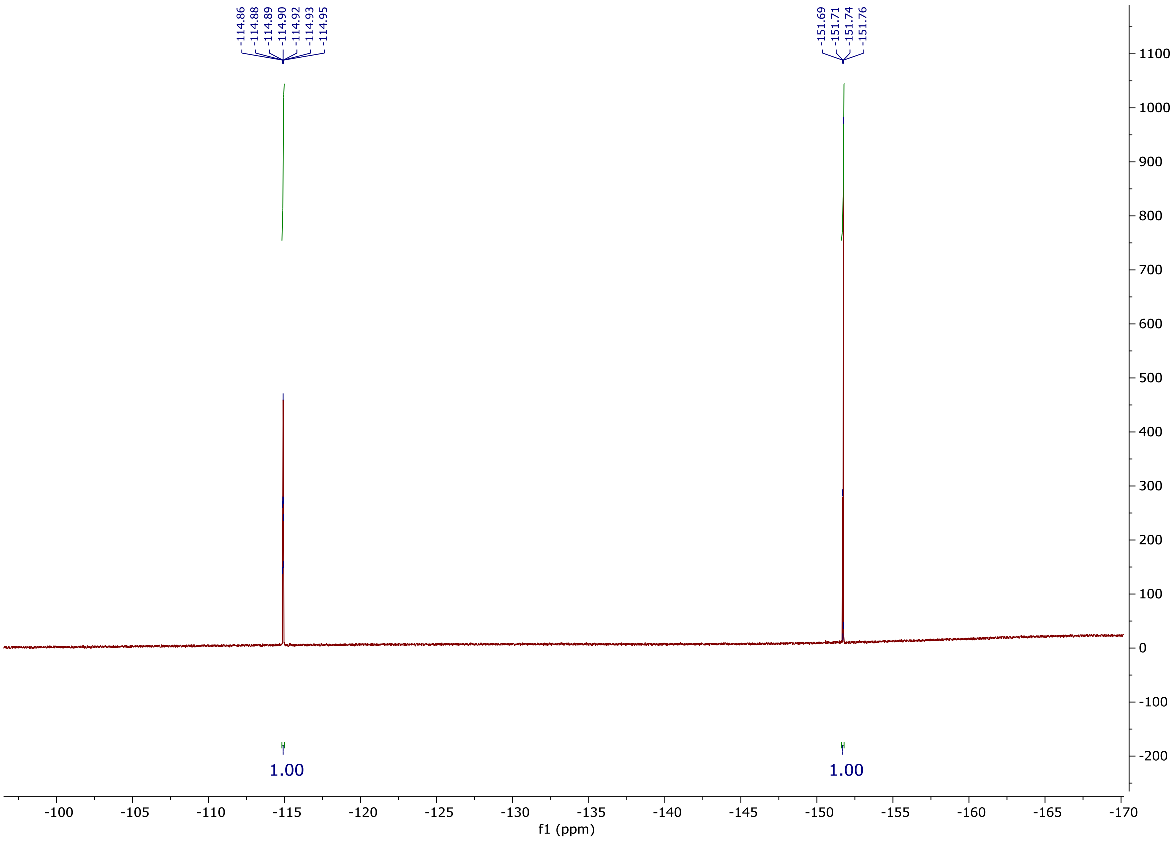

Figure S2. ^{19}F NMR spectrum of 4:1 equivalents of **2b** and fluorobenzene respectively illustrating complete conversion of $[AuBr_2(Me_2dazdt)]IBr_2$ to $[AuBr_2(Me_2dazdt)]BF_4$.

[Au_2Br_2(Me_2dazdt)]

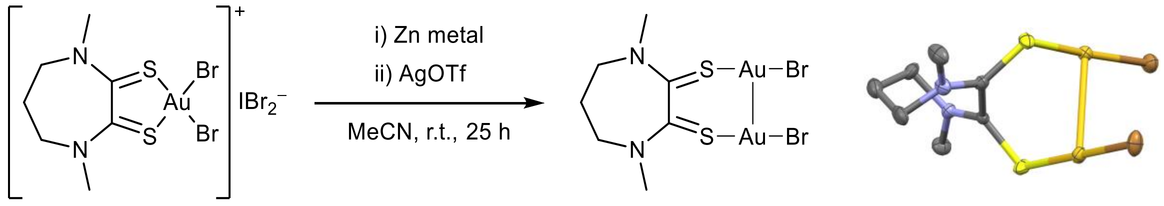

Scheme S5. Unsuccessful iodine dibromide anion metathesis yielding [Au_2Br_2(Me_2dazdt)].

A suspension of zinc powder (1.3 mg, 0.020 mmol) in a solution of [$AuBr_2$(Me_2dazdt)]IBr_2 (**2**) (17 mg, 0.020 mmol) in MeCN (20 mL) was stirred at room temperature. After 24 h, the reaction mixture was filtered under gravity and AgOTf (5 mg, 0.02 mmol) was added to the stirred filtrate, resulting in the immediate formation of an off-white precipitate. After 1 h, the suspension was filtered under gravity, the filtrate concentrated *in vacuo* and the resulting dark-red powder washed with MeOH (2 x 5 mL). The crude material was resuspended in MeCN (~3 mL) and recrystallized by vapor phase diffusion with $CHCl_3$, yielding [Au_2Br_2(Me_2dazdt)] as reflective red crystals suitable for single crystal XRD (2.6 mg, 35%).

1.3. Synthesis of propargylic amides

N-(Prop-2-yn-1-yl)benzamide

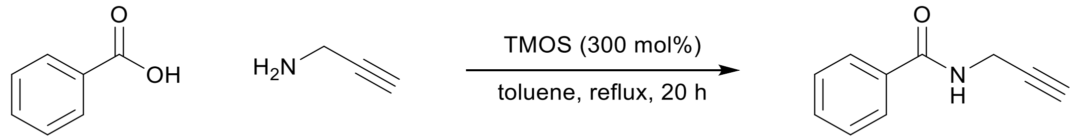

Scheme S6. Direct amidation of benzoic acid and propargylamine yielding *N-(Prop-2-yn-1-yl)benzamide*.

According to the procedure reported by Braddock *et al*,[S5] a solution of benzoic acid (5.00 mmol, 611 mg), propargylamine (5.00 mmol, 275 mg) and tetramethylorthosilicate (TMOS, 2.22 mL, 15.0 mmol) in toluene (8 mL) was stirred at reflux. After 20 h, the reaction solution was cooled to room temperature, concentrated and the resulting residue dissolved in THF (25 mL). The THF solution was washed with aqueous K_2CO_3 (75 mL, 0.3 M, 22.5 mmol). Solid NaCl was added continuously until two clear layers formed, the aqueous layer was extracted with THF (2 x 15 mL) and the combined organic layers washed with sat. NH_4Cl (25 mL). The organic layer was dried over Na_2SO_4, filtered and concentrated to obtain the desired product (638 mg, 80%) as a white crystalline solid. IR (ATR): 3282, 1637 cm^{-1}. ^1H NMR (400 MHz, Chloroform-*d*): δ 2.30 (t, J = 2.7 Hz, 1H), 4.28 (dd, J = 5.3 Hz, 2.6 Hz, 2H), 6.33 (s, 1H), 7.45

(m, 2H), 7.53 (tt, J = 7.3 Hz, 6.1 Hz, 2.5 Hz, 1.2 Hz, 1H), 7.80 (m, 2H) ppm. $^{13}C\{^1H\}$ (101 MHz, Chloroform-*d*): δ 167.1, 133.7, 131.8, 128.6, 127.0, 79.4, 71.9, 29.8 ppm.

N-(3-Phenylprop-2-yn-1-yl)benzamide

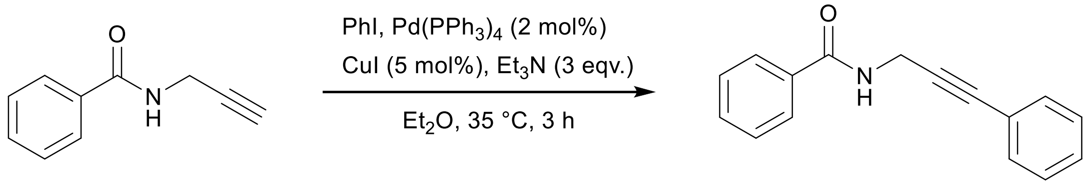

Scheme S7. Sonogashira cross-coupling yielding *N-(3-Phenylprop-2-yn-1-yl)benzamide.*

According to the procedure reported by Engle *et al.*,[S6] a solution of $Pd(PPh_3)_4$ (2 mol %, 23 mg, 0.02 mmol), CuI (5 mol %, 9.5 mg, 0.05 mmol), *N*-(prop-2-yn-1-yl)benzamide (1.00 mmol, 159 mg) in anhydrous diethyl ether (3 mL) was stirred at room temperature under an inert atmosphere of N_2. After 10 min, iodobenzene (2.00 mmol, 224 μL) was added, followed by the dropwise addition of diethylamine (3.00 mmol, 310 μL) and the reaction mixture stirred at 35 °C. After 3 h, the reaction mixture was poured into a separating funnel containing brine (20 mL) and extracted with Et_2O (3 x 25 mL). The combined organic layers were dried over Na_2SO_4, filtered and concentrated *in vacuo*. The crude residue was purified by silica gel column chromatography (R_f = 0.23 [*n*-hexane/EtOAc (4:1)]) yielding the desired amide (177 mg, 75%) as a white solid. ^1H NMR (400 MHz, Chloroform-*d*) δ 7.86 – 7.79 (m, 2H), 7.58 – 7.50 (m, 1H), 7.50 – 7.42 (m, 4H), 7.33 (m, 3H), 6.36 (s, 1H), 4.51 (d, J = 5.1 Hz, 2H)v ppm.

1.4. Catalytic testing of gold recovery products 1a-2b

1.4.1. Cyclization of *N*-propargylic amides

5-Methyl-2-phenyloxazole

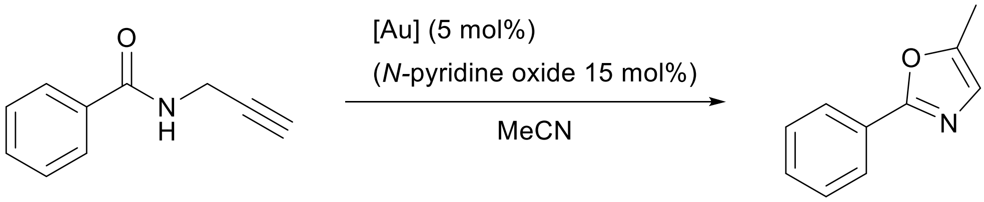

Scheme S8. Cyclisation of *N*-Propargylbenzamide yielding *5-Methyl-2-phenyloxazole.*

N-Propargylbenzamide (32 mg, 0.20 mmol), *N*-pyridine oxide (2.9 mg, 0.030 mmol) and the respective gold complex (0.01 mmol, 0.05 eq) were dissolved in acetonitrile (1.5 mL). The solution was stirred for the amount of time and at the temperature indicated in the main

manuscript (Table 1). After the indicated time, the reaction solution was concentrated and the yield quantified by ^{1}H NMR analysis. Yields were determined by the relative integrals of the amide methylene protons (4.23- 4.25 ppm) and the product vinyl proton (6.85 ppm). A pure sample of the cyclized product was isolated following purification by silica gel column chromatography (R_f = 0.25 [*n*-hexane/EtOAc (7:1)]) yielding the desired oxazole (^{1}H NMR Yield: 96%. Isolated Yield: 28 mg, 89%) as a white solid. ^{1}H NMR (400 MHz, Chloroform-*d*): δ 8.06 – 7.98 (m, 2H), 7.51 – 7.39 (m, 3H), 6.86 (d, *J* = 1.4 Hz, 1H), 2.42 (d, *J* = 1.2 Hz, 3H) ppm. ^{13}C{^{1}H} NMR (101 MHz, Chloroform-*d*): δ 160.7, 148.9, 129.9, 128.7, 127.8, 126.0, 124.2, 11.1 ppm.

(Z)-5-Benzylidene-2-phenyl-4,5-dihydrooxazole **and** ***(E)-5-(iodo(phenyl)methylene)-2-phenyl-4,5-dihydrooxazole***

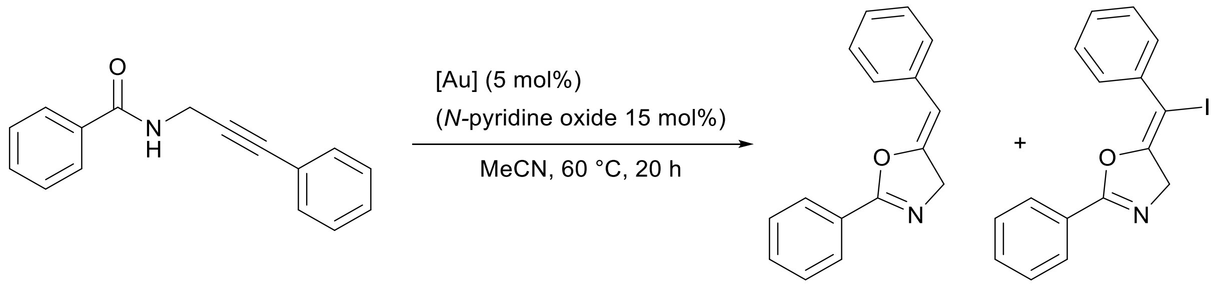

Scheme S9. Cyclisation of *N*-(3-phenylprop-2-yn-1-yl)benzamide.

A stirred solution of amide (56 mg, 0.24 mmol), *N*-pyridine oxide (3.4 mg, 0.036 mmol) and [Au] (5 mol%, 0.012 mmol) in MeCN (1.8 mL) was heated to 60 ºC for 20 h. The reaction mixture was concentrated *in vacuo* and the resulting residue purified by silica gel column chromatography [*n*-hexane/EtOAc (7:1)]. Results for each catalyst are presented in the main text (Table 2).

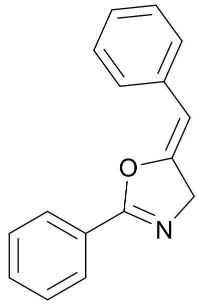

R_f = 0.18. HRMS (ESI) *m/z* [M+H]$^{+}$ calcd. for $C_{16}H_{14}NO$: 236.1075, found: 236.1077. ^{1}H NMR (400 MHz, Chloroform-*d*): δ 8.10-8.07 (m 1H), 7.66 – 7.61 (m, 2H), 7.58 – 7.51 (m, 3H), 7.43 – 7.39 (m, 3H), 7.28 – 7.21 (m, 1H), 5.68 (t, *J* = 2.6 Hz, 1H), 4.84 (d, *J* = 2.6 Hz, 2H) ppm. ^{13}C{^{1}H} NMR (101 MHz, Chloroform-*d*): δ 164.1, 151.8, 135.0, 132.2, 130.7, 128.8, 128.6, 128.3, 127.9, 126.3, 100.8, 59.5 ppm.

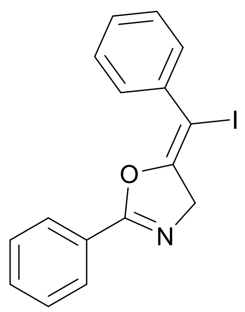

R_f = 0.20. HRMS (ESI) *m/z* $[M+H]^+$ calcd. for $C_{16}H_{13}INO$: 362.0042, found: 362.0047. ^1H NMR (400 MHz, Chloroform-*d*) δ 7.93 (d, *J* = 7.6 Hz, 2H), 7.68 (d, *J* = 7.7 Hz, 2H), 7.58 – 7.19 (m, 6H), 4.88 (s, 2H) ppm. $^{13}C\{^1H\}$ NMR (101 MHz, Chloroform-*d*): δ 164.2, 152.8, 138.1, 132.3, 129.6, 128.8, 128.3, 128.1, 127.9, 126.7, 70.8, 65.2 ppm.

Lithiation and hydrolysis of *(E)-5-(iodo(phenyl)methylene)-2-phenyl-4,5-dihydrooxazole*

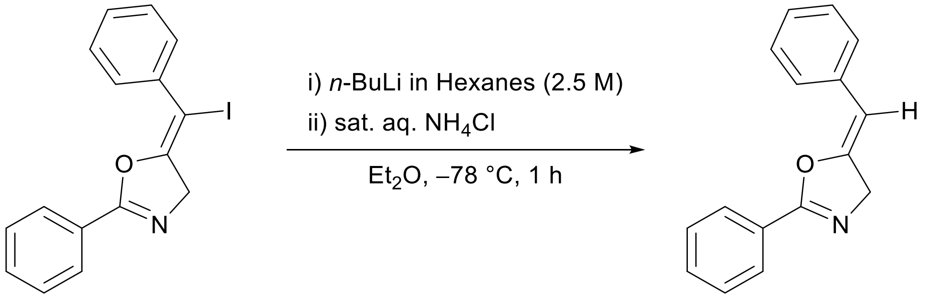

Scheme S10. Confirmation of 5-exo cyclisation by lithiation and hydrolysis of of *(E)-5-(iodo(phenyl)methylene)-2-phenyl-4,5-dihydrooxazole*.

An Et_2O (1 mL) solution of the vinyl iodide oxazole (30 mg, 0.083 mmol) was cooled to –78 °C. A solution of *n*-BuLi in Hexanes (2.5 M, 0.087 mmol, 34.9 μL) was added dropwise over 10 minutes. After 1 h, IPA (1 mL) was added dropwise, the mixture was warmed to room temperature and a saturated solution of ammonium chloride (1 mL) was added dropwise to the Et_2O solution. The organic layer was isolated, washed with brine and dried over $MgSO_4$. The residue was purified by silica-gel chromatography (R_f = 0.18, [petroleum ether (40-60)/ethyl acetate (7:1)]), yielding the title compound as a white solid (7 mg, 35%). Spectroscopic data obtained were identical to those reported above.

1.4.2. Condensation of *o*-iodoaniline and acetylacetone

(Z)-4-((2-Iodophenyl)amino)pent-3-en-2-one

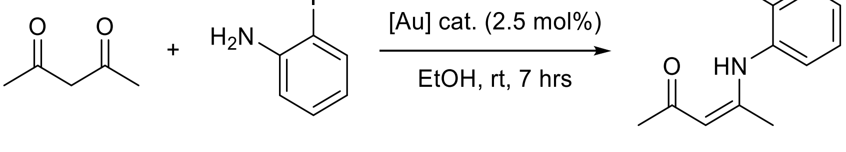

Scheme S11. Condensation of *o*-iodoaniline and acetylacetone.

2-Iodoaniline (44 mg, 0.2 mmol) and [Au] (0.005 mmol, 0.025 eq) were dissolved in ethanol (3 mL) and acetylacetone (20 µL, 0.2 mmol) was added to the solution. After 7 h, the reaction mixture was concentrated, the residue dissolved in $CDCl_3$ and submitted for ^1H NMR analysis. Yields were determined by the relative integrals of the *o*-iodoaniline starting material (dd 7.65 ppm) and the product (dd 7.91 ppm). Average yields with standard deviations are provided for each catalyst in the main manuscript (Table 3).

Condensation of *o*-iodoaniline and acetylacetone – Catalyst recycling

2-Iodoaniline (44 mg, 0.2 mmol) and [Au] (0.005 mmol, 0.025 eq) were dissolved in ethanol (3 mL) and acetylacetone (20 µL, 0.2 mmol) was added to the solution. After 7 h, the reaction mixture was concentrated and the crude oil triturated with *n*-hexane (2 x 5 mL). The combined *n*-hexane fractions were concentrated, yielding a crude mixture of *o*-iodoanisole and *(Z)-4-((2-iodophenyl)amino)pent-3-en-2-one*. The black residue containing the gold catalyst material was then reused 8 times with no observed loss in catalytic activity.

1.4.3. Addition of nucleophilic arenes to α,β-unsaturated carbonyl compounds

4-(5-Methylfuran-2-yl)butan-2-one

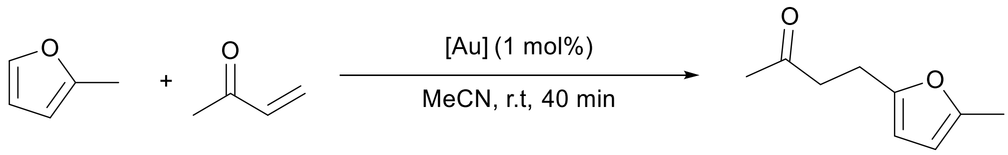

Scheme S12. Addition of 2-methylfuran to methyl vinyl ketone.

A solution of 2-methylfuran (0.089 mL, 82 mg, 1.0 mmol), methyl vinyl ketone (0.083 mL, 70 mg, 1.0 mmol) and [Au] catalyst (0.010 mmol, 1 mol%) in MeCN (4 mL) was stirred at room temperature. After 40 min, the reaction mixture was concentrated and the crude material triturated with *n*-hexane (2 x 5 mL). The combined *n*-hexane fractions were concentrated to yield the pure desired product as a colorless oil. ^1H NMR (400 MHz, Chloroform-*d*) δ 5.90 – 5.80 (m, 2H), 2.90 – 2.81 (m, 2H), 2.78-2.73 (m, 2H), 2.24 (s, 3H), 2.16 (s, 3H) ppm. The black residue containing the gold catalyst material was then reused 5 times with no observed loss in catalytic activity.

4-(5-Methylfuran-2-yl)butan-2-one

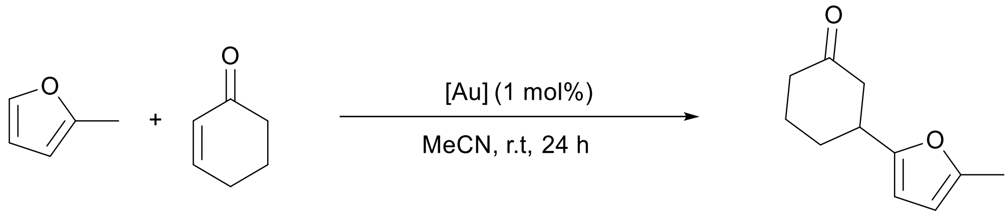

Scheme S13. Addition of 2-methylfuran to cyclohex-2-en-1-one.

A solution of 2-methylfuran (0.089 mL, 82 mg, 1.0 mmol), cyclohex-2-en-1-one (0.097 ml, 96 mg, 1.0 mmol) and [Au] catalyst (0.010 mmol, 1 mol%) in MeCN (4 mL) was stirred at room temperature. After 24 h, the reaction mixture was concentrated and the crude material triturated with *n*-hexane (2 x 5 mL). The combined *n*-hexane fractions were concentrated to yield the pure desired product as a colorless oil. ^{1}H NMR (400 MHz, Chloroform-*d*) δ 5.90 – 5.84 (m, 2H), 3.17-3.09 (m, 1H), 2.69-2.64 (m, 1H), 2.55-2.48 (m, 1H), 2.47 – 2.28 (m, 2H), 2.26 (s, 3H), 2.23 – 2.08 (m, 1H), 2.07-1.99 (m, 1H), 1.92 – 1.70 (m, 2H) ppm. The black residue containing the gold catalyst material was then reused 5 times with no observed loss in catalytic activity.

4,4'-(Azulene-1,3-diyl)bis(butan-2-one)

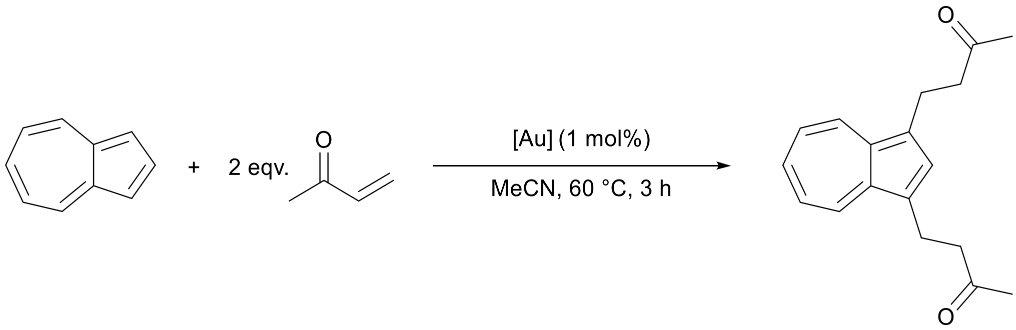

Scheme S14. Addition of azulene to methyl vinyl ketone.

A stirred solution of azulene (40 mg, 0.3 mmol), methyl vinyl ketone (0.052 ml, 44 mg, 0.62 mmol) and [Au)] (0.0031 mmol, 1 mol%) in MeCN (1.7 ml) was heated at 60 ºC. After 3 h, the reaction mixture was concentrated, diluted with $CDCl_3$ and submitted for ^{1}H NMR analysis. In all three runs for each gold catalyst, only trace quantities of unreacted azulene were detectable by ^{1}H NMR analysis of the crude product. ^{1}H NMR (400 MHz, Chloroform-*d*) δ 8.20 (d, *J* = 9.3 Hz, 2H), 7.61 (s, 1H), 7.50 (t, *J* = 9.8, 1H), 7.01 (t, *J* = 9.8 Hz, 2H), 3.34-3.30 (m, 4H), 2.90-2.86 (m, 4H), 2.15 (s, 6H) ppm.

4,4'-(4,6-Dimethoxy-1,3-phenylene)bis(butan-2-one)

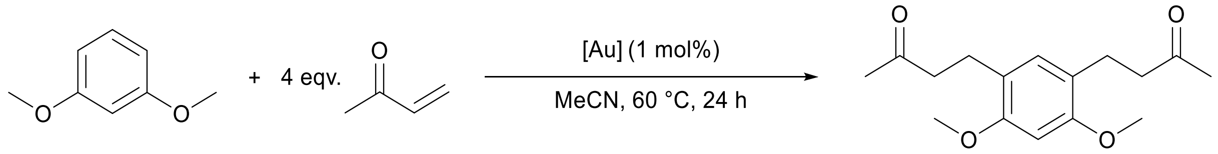

Scheme S15. Addition of 1,3-dimethoxybenzene to methyl vinyl ketone.

A stirred solution of 1,3-dimethoxybenzene (0.131 mL, 138 mg, 1.00 mmol), methyl vinyl ketone (0.332 mL, 278 mg, 4.00 mmol) and [Au] (0.010 mmol, 1 mol%) in MeCN (4 mL) was stirred at 60 ºC. After 24 h, the reaction mixture was concentrated, the residue diluted in $CDCl_3$ and the yield determined by ^{1}H NMR analysis, through comparison of the integration of the dialkylated Ar-H singlet at 6.86 ppm, the monoalkylated doublet at 7.02-7.00 ppm and the starting material triplet at 7.21 ppm. Results are shown in Table S1 below:

Table S1. Results for the addition of 1,3-dimethoxybenzene to methyl vinyl ketone using **1a**, **2a**, **1b** and **2b**.

Run	**1a**		**2a**	
	Monoalkylated yield (%/^{1}H NMR)	Dialkylated yield (%/^{1}H NMR)	Monoalkylated yield (%/^{1}H NMR)	Dialkylated yield (%/^{1}H NMR)
1	5	93	12	79
2	6	90	14	76
3	4	93	12	78

Run	**1b**		**2b**	
	Monoalkylated yield (%/^{1}H NMR)	Dialkylated yield (%/^{1}H NMR)	Monoalkylated yield (%/^{1}H NMR)	Dialkylated yield (%/^{1}H NMR)
1	35	33	27	20
2	37	34	22	18
3	33	27	28	27

1.4.4. Oxidative coupling of aryl silanes and arenes

General cross-coupling procedure

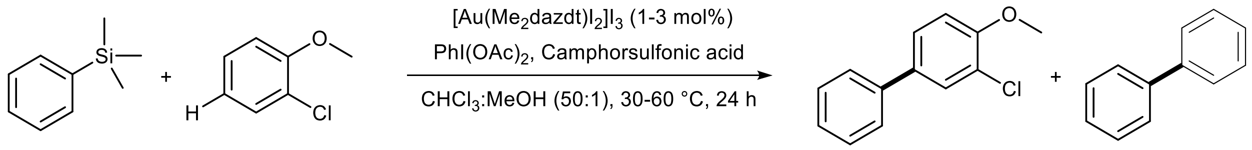

Scheme S16. Gold(III) catalysed cross-coupling of arenes and aryl silanes.

Phenyltrimethylsilane (0.50 mmol, 86 µL), *o*-chloroanisole (0.50 mmol, 64 µL) and 1,4-dinitrobenzene (Internal standard, 0.125 mmol, 21.0 mg) were added to a solution of $[AuI_2(Me_2dazdt)]I_3$ (0.010 mmol, 10 mg) in $CHCl_3$ (5 mL) and MeOH (0.1 mL). Iodobenzene diacetate (209 mg, 0.65 mmol) and camphorsulfonic acid (174 mg, 0.75 mmol) were added and the reaction stirred. After 24 h, an aliquot (100 µL) of the reaction mixture was diluted in MeCN (10 mL) and the diluted sample submitted for HPLC analysis. The reaction variables and variable limits modelled in the DoE optimisation are summarized in Table S2.

Table S2. Design of experiments definitive screening design – Variables and variable limits.

Factor	Minimum	Maximum
Catalyst loading (mol%)	1	3
Silver loading (mol%)	0	6
Temperature (°C)	30*	60
Concentration (M)	0.1	0.25
Arene equivalence	1	1.5

* A lower temperature limit of 30 °C was set to avoid overnight changes in ambient temperature

Models for both cross-coupling and homo-coupling yields were produced using a definitive screening design (Table S3).

DoE procedure.

Stock solutions containing phenyltrimethylsilane and 1,4-dinitrobenzene (internal standard), and *o*-chloroanisole were used throughout the design of experiments screen. The stock solutions were combined and diluted appropriately in ReactArray reaction vials containing an appropriate quantity of $[AuI_2(Me_2dazdt)]I_3$ to produce the conditions indicated for each entry in the DoE data table (Table S3). Appropriate quantities of iodobenzene diacetate and camphorsulfonic acid were then added to initiate the reaction, and the reaction stirred at the indicated temperature. DoE model validation and prediction profiles are provided in Figures

S3 and S4 for the cross-coupling product, and Figures S5 and S6 for the homo-coupling product.

Table S3. DoE design and results table.

Catalyst Loading (mol%)	Concentration (M)	Silver Loading (mol%)	Temperature (°C)	Arene equivalence	Cross-coupling yield (HPLC/%)	Homo-coupling yield (HPLC/%)
**2**	0.1	0	30	1	10	17
**2**	0.25	6	60	1.5	64	9
**2**	0.175	3	45	1.25	54	16
**1**	0.1	6	30	1.5	60	17
**3**	0.1	6	60	1.25	45	18
**3**	0.25	0	60	1.5	57	12
**1**	0.25	6	45	1	36	22
**1**	0.1	0	60	1	49	16
**1**	0.1	0	60	1.5	66	13
**1**	0.175	6	60	1.5	69	14
**3**	0.1	0	45	1.5	69	12
**3**	0.1	3	30	1.5	39	13
**1**	0.25	3	60	1	44	18
**1**	0.1	6	30	1	29	13
**3**	0.25	0	60	1	47	16
**1**	0.25	0	30	1.25	49	20
**3**	0.175	0	30	1	37	12
**3**	0.1	6	60	1	41	20
**3**	0.25	6	30	1.5	80	7
**3**	0.25	6	30	1	49	11
**1**	0.25	0	30	1.5	74	9

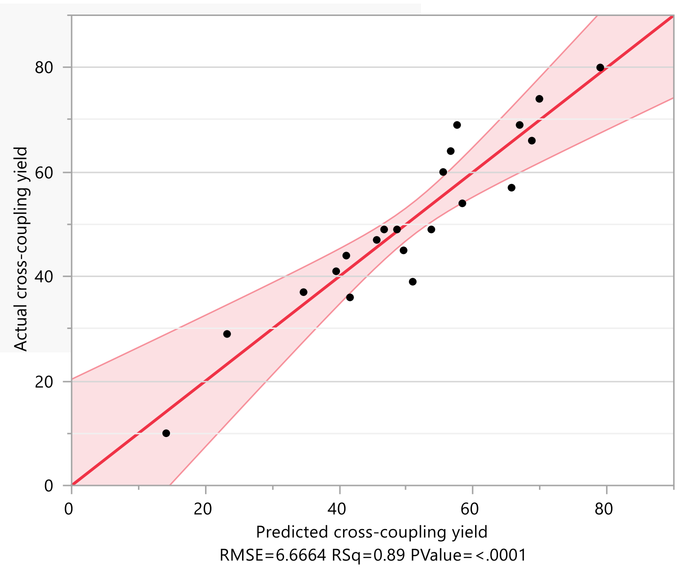

Figure S3. Cross-coupling definitive screening design showing actual vs predicted cross-coupling yield.

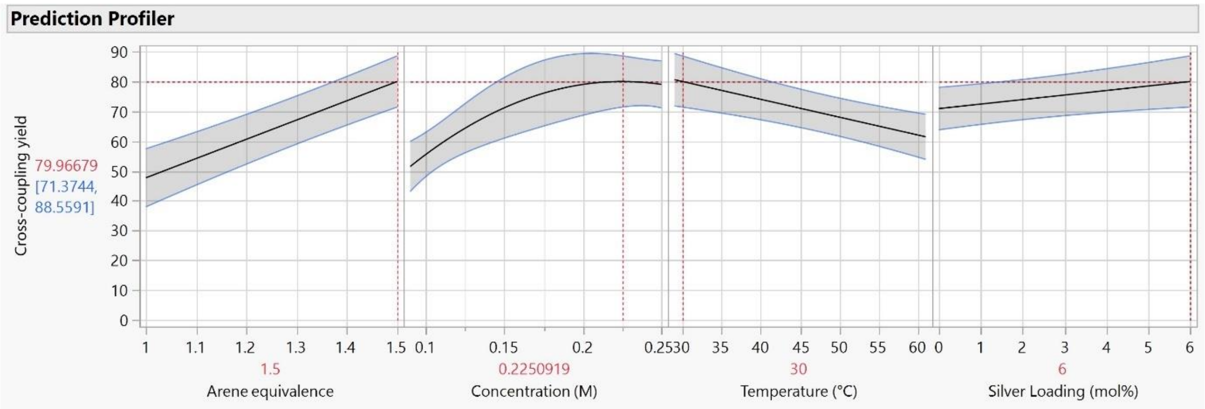

Figure S4. Cross-coupling definitive screening design showing prediction profiles illustrating optimum reaction conditions.

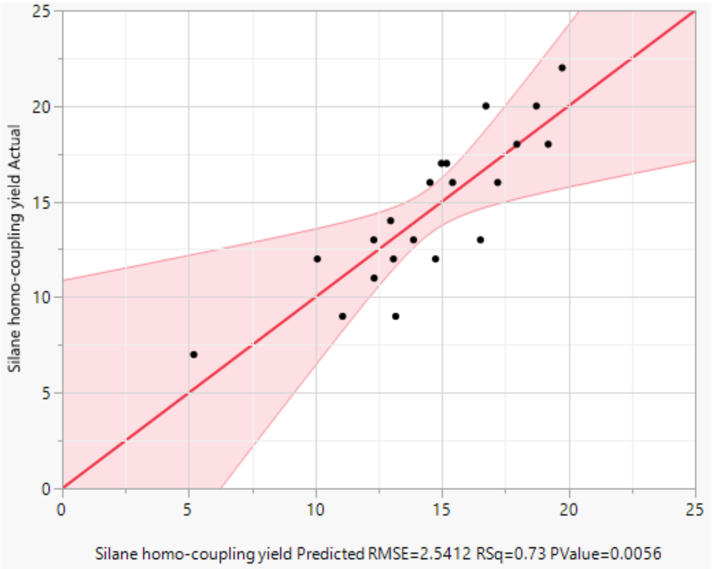

Figure S5. Silane homo-coupling definitive screening design showing actual vs predicted cross-coupling yield.

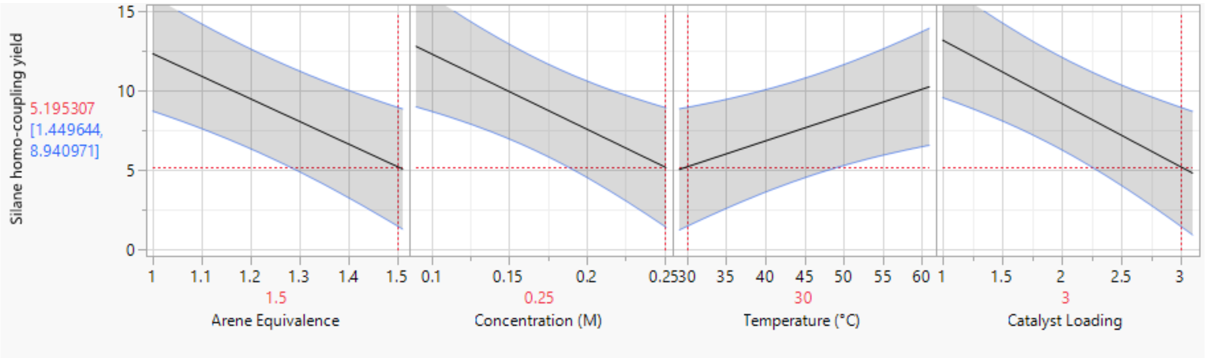

Figure S6. Silane homo-coupling definitive screening design showing prediction profiles illustrating optimum reaction conditions.

Optimized cross-coupling procedure and scope

Phenyltrimethylsilane (0.50 mmol, 0.075 g, 86 µL) and arene (0.75 mmol) were added to a vial containing [Au] (1.0 mol%, 0.005 mmol) in $CHCl_3$ (2.2 mL, 0.23 M) and MeOH (100 µL). Iodobenzene diacetate (209 mg, 0.649 mmol) and camphorsulfonic acid (174 mg, 0.749 mmol) were added, and the reaction was stirred at room temperature. After 24 h, the reaction mixture was concentrated *in vacuo* and the crude material purified by column chromatography.

3-Chloro-4-methoxy-1,1'-biphenyl

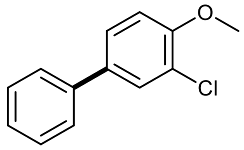

White solid (**1a**: 77 mg, 70%; **2a**: 82 mg, 75%); R_f = 0.26 [*n*-hexane /toluene (9:1)]; ^{1}H NMR (400 MHz, Chloroform-*d*): δ 7.63 (d, *J* = 2.3 Hz, 1H), 7.58 – 7.51 (m, 2H), 7.51 – 7.39 (m, 3H), 7.39 – 7.30 (m, 1H), 7.01 (d, *J* = 8.5 Hz, 1H), 3.96 (s, 3H) ppm. ^{13}C{^{1}H} NMR (101 MHz, Chloroform-*d*) δ 154.5, 139.7, 134.8, 129.0, 127.4, 126.84, 126.4, 122.9, 112.4, 56.4 ppm.

3-Bromo-4-methoxy-1,1'-biphenyl

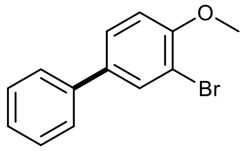

White solid (**1a**: 87 mg, 74%; **2a**: 94 mg, 80%); R_f = 0.26 [*n*-hexane/toluene (9:1)]; ^{1}H NMR (400 MHz, Chloroform-*d*): δ 7.83 (d, *J* = 2.3 Hz, 1H), 7.59 – 7.49 (m, 3H), 7.49 – 7.40 (m, 2H), 7.40 – 7.31 (m, 1H), 6.98 (d, *J* = 8.5 Hz, 1H), 3.95 (s, 3H) ppm. ^{13}C{^{1}H} NMR (101 MHz, Chloroform-*d*) δ 155.3, 139.5, 135.2, 132.0, 128.9, 127.3, 127.1, 126.8, 112.2, 112.1, 56.4 ppm.

3,4-Dimethyl-1,1'-biphenyl

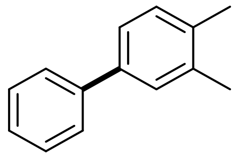

White solid (**1a**: 56 mg, 61%; **2a**: 64 mg, 70%); R_f = 0.31 [*n*-hexane/toluene (100:1)]; ^{1}H NMR (400 MHz, Chloroform-*d*) δ 7.64 (m, 2H), 7.52 – 7.33 (m, 5H), 7.27 (d, *J* = 7.8 Hz, 1H), 2.39

(s, 3H), 237 (s, 3H) ppm. $^{13}C\{^1H\}$ NMR (101 MHz, Chloroform-*d*) δ 141.4, 139.0, 137.0, 135.8, 130.2, 128.8, 128.6, 128.6, 127.1, 127.0, 124.6, 20.1, 19.6 ppm.

4-Methyl-1,1'-biphenyl

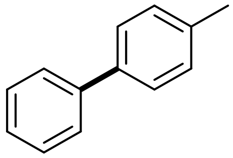

White solid (**1a**: 50 mg, 59%; **2a**: 55 mg, 65%); R_f = 0.31 [*n*-hexane/toluene (100:1)]; ^1H NMR (400 MHz, Chloroform-*d*): δ 7.65 – 7.58 (m, 2H), 7.57 – 7.50 (m, 2H), 7.46 (dd, *J* = 8.4, 6.9 Hz, 2H), 7.40 – 7.32 (m, 1H), 7.29 (d, *J* = 7.9 Hz, 2H), 2.43 (s, 3H) ppm. $^{13}C\{^1H\}$ NMR (101 MHz, Chloroform-*d*): δ 141.3, 138.5, 137.2, 129.6, 128.9, 127.1, 127.1, 21.2 ppm.

1-Methyl-4-phenylnaphthalene

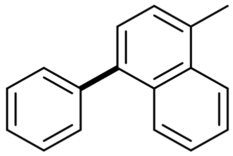

Colorless oil (**1a**: 57 mg, 52%; **2a**: 60 mg, 55%); R_f = 0.61 ([petroleum ether]); ^1H NMR (400 MHz, Chloroform-*d*) δ 8.26 – 8.18 (m, 1H), 8.12 (t, *J* = 6.6 Hz, 1H), 7.74 – 7.44 (m, 9H), 2.93 – 2.87 (m, 3H) ppm. $^{13}C\{^1H\}$ NMR (101 MHz, Chloroform-*d*): δ 141.2, 138.8, 133.9, 132.9, 131.8, 130.3, 128.3, 127.2, 126.8, 126.7, 126.3, 125.8, 124.5, 19.7 ppm.

2-Bromo-5-phenylthiophene

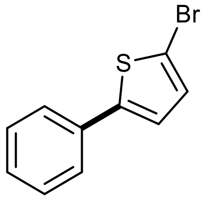

White solid (**1a**: 102 mg, 85%; **2a**: 108 mg, 90%); R_f = 0.27 [petroleum ether]); ^1H NMR (400 MHz, Chloroform-*d*): δ 7.58 – 7.50 (m, 2H), 7.45 – 7.36 (m, 2H), 7.36 – 7.28 (m, 1H), 7.11 – 7.03 (m, 2H) ppm. $^{13}C\{^1H\}$ NMR (101 MHz, Chloroform-*d*): δ 146.0, 133.8, 131.0, 129.1, 128.0, 125.7, 123.4, 111.5 ppm.

1.5. X-Ray crystallographic information

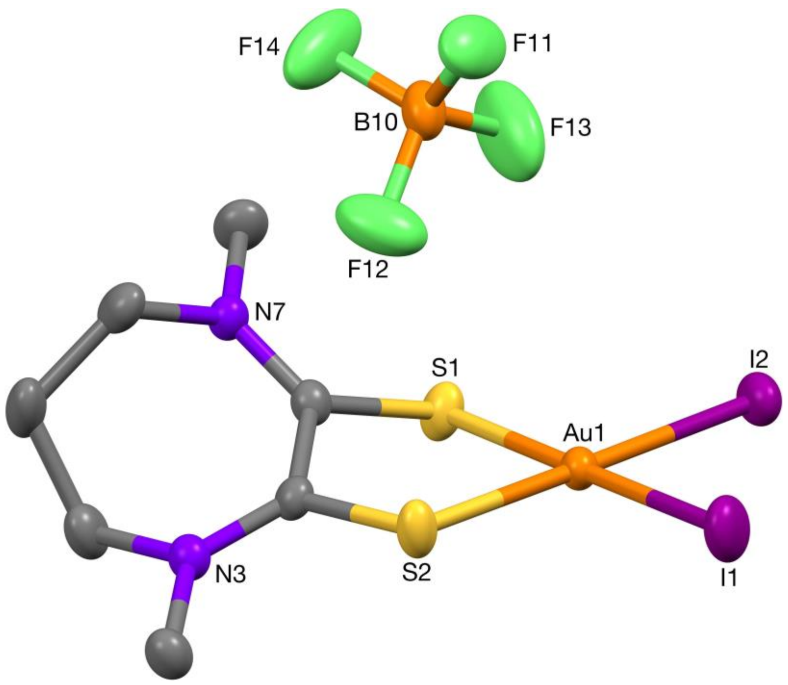

Figure S7. The X-ray crystal structure of **1b** (thermal ellipsoids drawn at 50% probability).

Crystal data for **1b**: $[C_7H_{12}AuI_2N_2S_2](BF_4)$, M = 725.88, monoclinic, $P2_1/n$ (no. 14), a = 14.2058(2), b = 8.14668(11), c = 15.1256(2) Å, β = 105.7976(17)°, V = 1684.37(5) Å^3, Z = 4, D_c = 2.862 g cm^{-3}, μ(Mo-Kα) = 12.673 mm^{-1}, T = 173 K, black blocks, Agilent Xcalibur 3 E diffractometer; 3979 independent measured reflections (R_{int} = 0.0416), F^2 refinement,[S7,S8] R_1(obs) = 0.0204, wR_2(all) = 0.0396, 3625 independent observed absorption-corrected reflections [$|F_o| > 4\sigma(|F_o|)$, completeness to θ_{full}(25.2°) = 99.9%], 196 parameters. CCDC 2156494.

The B10-based tetrafluoroborate anion in the structure of **1b** was found to be disordered. Two orientations were identified of *ca*. 87 and 13% occupancy, their geometries were optimized, the thermal parameters of adjacent atoms were restrained to be similar, and only the atoms of the major occupancy orientation were refined anisotropically (those of the minor occupancy orientation were refined isotropically).

Table S4. Table of **1b** bond lengths.

Number	Atom1	Atom2	Cyclicity	Length
1	Au1	I1	acyclic	2.6064(3)
2	Au1	I2	acyclic	2.6093(4)
3	Au1	S1	cyclic	2.3243(9)
4	Au1	S2	cyclic	2.3250(9)
5	S1	C1	cyclic	1.717(3)
6	C1	C2	cyclic	1.491(5)
7	C1	N7	cyclic	1.297(5)
8	C2	S2	cyclic	1.714(3)
9	C2	N3	cyclic	1.306(4)
10	N3	C4	cyclic	1.483(5)
11	N3	C8	acyclic	1.473(4)
12	C4	H4A	acyclic	0.99
13	C4	H4B	acyclic	0.99
14	C4	C5	cyclic	1.519(7)
15	C5	H5A	acyclic	0.99
16	C5	H5B	acyclic	0.99
17	C5	C6	cyclic	1.519(5)
18	C6	H6A	acyclic	0.989
19	C6	H6B	acyclic	0.99
20	C6	N7	cyclic	1.489(5)
21	N7	C9	acyclic	1.475(4)
22	C8	H8A	acyclic	0.981
23	C8	H8B	acyclic	0.98
24	C8	H8C	acyclic	0.98
25	C9	H9A	acyclic	0.98
26	C9	H9B	acyclic	0.98
27	C9	H9C	acyclic	0.98
28	B10	F11	acyclic	1.379(8)
29	B10	F12	acyclic	1.396(6)
30	B10	F13	acyclic	1.357(8)
31	B10	F14	acyclic	1.370(7)

Table S5. Table of **1b** bond angles

Number	Atom1	Atom2	Atom3	Angle	Number	Atom1	Atom2	Atom3	Angle
1	I1	Au1	I2	93.60(1)	29	H5B	C5	C6	109.3
2	I1	Au1	S1	177.77(3)	30	C5	C6	H6A	109.4
3	I1	Au1	S2	87.03(3)	31	C5	C6	H6B	109.4
4	I2	Au1	S1	88.09(3)	32	C5	C6	N7	111.2(3)
5	I2	Au1	S2	179.33(3)	33	H6A	C6	H6B	108.1
6	S1	Au1	S2	91.28(3)	34	H6A	C6	N7	109.4
7	Au1	S1	C1	97.7(1)	35	H6B	C6	N7	109.3
8	S1	C1	C2	117.7(3)	36	C1	N7	C6	120.6(3)
9	S1	C1	N7	122.6(3)	37	C1	N7	C9	120.6(3)
10	C2	C1	N7	119.7(3)	38	C6	N7	C9	118.7(3)
11	C1	C2	S2	117.9(3)	39	N3	C8	H8A	109.4
12	C1	C2	N3	119.6(3)	40	N3	C8	H8B	109.5
13	S2	C2	N3	122.5(3)	41	N3	C8	H8C	109.5
14	Au1	S2	C2	99.9(1)	42	H8A	C8	H8B	109.4
15	C2	N3	C4	121.0(3)	43	H8A	C8	H8C	109.4
16	C2	N3	C8	121.6(3)	44	H8B	C8	H8C	109.5
17	C4	N3	C8	117.4(3)	45	N7	C9	H9A	109.5
18	N3	C4	H4A	109.2	46	N7	C9	H9B	109.5
19	N3	C4	H4B	109.2	47	N7	C9	H9C	109.5
20	N3	C4	C5	111.9(3)	48	H9A	C9	H9B	109.5
21	H4A	C4	H4B	107.9	49	H9A	C9	H9C	109.5
22	H4A	C4	C5	109.3	50	H9B	C9	H9C	109.5
23	H4B	C4	C5	109.3	51	F11	B10	F12	108.5(5)
24	C4	C5	H5A	109.3	52	F11	B10	F13	112.3(5)
25	C4	C5	H5B	109.3	53	F11	B10	F14	109.6(5)
26	C4	C5	C6	111.6(3)	54	F12	B10	F13	107.7(5)
27	H5A	C5	H5B	107.9	55	F12	B10	F14	106.2(5)
28	H5A	C5	C6	109.3	56	F13	B10	F14	112.3(5)

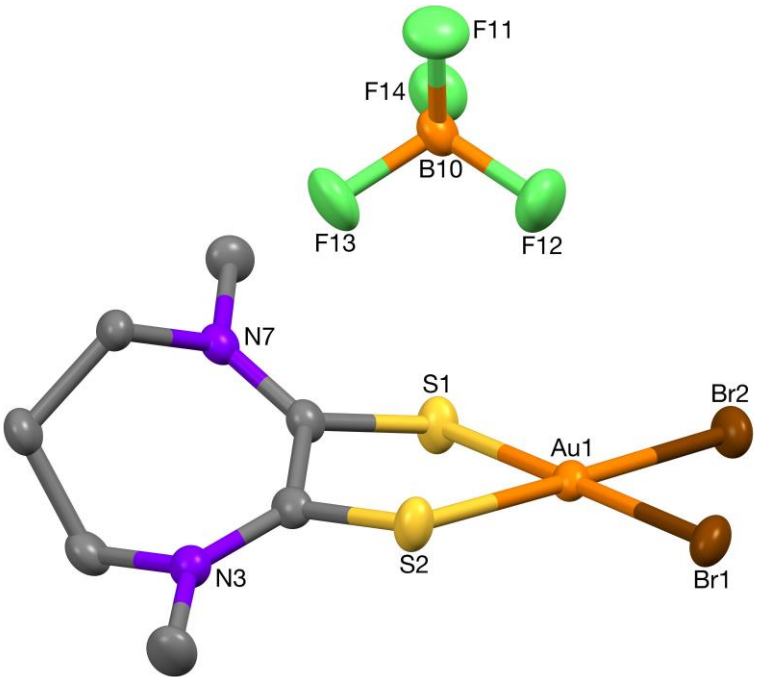

Figure S8. The X-ray crystal structure of **2b** (thermal ellipsoids drawn at 50% probability).

Crystal data for **2b**: $[C_7H_{12}AuBr_2N_2S_2](BF_4)$, M = 631.90, triclinic, P-1 (no. 2), a = 7.5558(3), b = 8.0088(3), c = 13.3746(4) Å, α = 99.789(3), β = 94.425(3), γ = 109.021(3)°, V = 746.34(5) Å^3, Z = 2, D_c = 2.812 g cm^{-3}, μ(Mo-Kα) = 15.517 mm^{-1}, T = 173 K, dark red blocks, Agilent Xcalibur 3 E diffractometer; 3255 independent measured reflections (R_{int} = 0.0301), F^2 refinement,[S7,S8] R_1(obs) = 0.0230, wR_2(all) = 0.0522, 3016 independent observed absorption-corrected reflections [$|F_o| > 4\sigma(|F_o|)$, completeness to θ_{full}(25.2°) = 100%], 175 parameters. CCDC 2156495.

Table S6. Table of **2b** bond lengths.

Number	Atom1	Atom2	Cyclicity	Length
1	Au1	Br1	acyclic	2.4605(5)
2	Au1	Br2	acyclic	2.4282(6)
3	Au1	S1	cyclic	2.302(1)
4	Au1	S2	cyclic	2.293(1)
5	S1	C1	cyclic	1.707(5)
6	C1	C2	cyclic	1.499(8)
7	C1	N7	cyclic	1.294(6)
8	C2	S2	cyclic	1.715(4)
9	C2	N3	cyclic	1.303(6)
10	N3	C4	cyclic	1.487(6)
11	N3	C8	acyclic	1.467(7)
12	C4	H4A	acyclic	0.99
13	C4	H4B	acyclic	0.99
14	C4	C5	cyclic	1.524(8)
15	C5	H5A	acyclic	0.99
16	C5	H5B	acyclic	0.99
17	C5	C6	cyclic	1.510(7)
18	C6	H6A	acyclic	0.99
19	C6	H6B	acyclic	0.99
20	C6	N7	cyclic	1.487(7)
21	N7	C9	acyclic	1.470(7)
22	C8	H8A	acyclic	0.98
23	C8	H8B	acyclic	0.98
24	C8	H8C	acyclic	0.98
25	C9	H9A	acyclic	0.98
26	C9	H9B	acyclic	0.98
27	C9	H9C	acyclic	0.98
28	B10	F11	acyclic	1.384(8)
29	B10	F12	acyclic	1.377(7)
30	B10	F13	acyclic	1.379(6)
31	B10	F14	acyclic	1.393(8)

Table S7. Table of **2b** bond angles.

Number	Atom1	Atom2	Atom3	Angle	Number	Atom1	Atom2	Atom3	Angle
1	Br1	Au1	Br2	94.33(2)	29	H5B	C5	C6	109.6
2	Br1	Au1	S1	179.30(4)	30	C5	C6	H6A	109.1
3	Br1	Au1	S2	87.44(4)	31	C5	C6	H6B	109.1
4	Br2	Au1	S1	86.37(4)	32	C5	C6	N7	112.6(4)
5	Br2	Au1	S2	178.22(4)	33	H6A	C6	H6B	107.8
6	S1	Au1	S2	91.86(5)	34	H6A	C6	N7	109.1
7	Au1	S1	C1	99.4(2)	35	H6B	C6	N7	109.1
8	S1	C1	C2	118.2(4)	36	C1	N7	C6	120.6(4)
9	S1	C1	N7	122.3(4)	37	C1	N7	C9	120.6(4)
10	C2	C1	N7	119.5(5)	38	C6	N7	C9	118.5(4)
11	C1	C2	S2	117.5(4)	39	N3	C8	H8A	109.5
12	C1	C2	N3	119.7(4)	40	N3	C8	H8B	109.4
13	S2	C2	N3	122.8(4)	41	N3	C8	H8C	109.4
14	Au1	S2	C2	100.3(2)	42	H8A	C8	H8B	109.5
15	C2	N3	C4	121.5(4)	43	H8A	C8	H8C	109.5
16	C2	N3	C8	121.8(5)	44	H8B	C8	H8C	109.5
17	C4	N3	C8	116.8(4)	45	N7	C9	H9A	109.4
18	N3	C4	H4A	109.4	46	N7	C9	H9B	109.4
19	N3	C4	H4B	109.4	47	N7	C9	H9C	109.5
20	N3	C4	C5	111.1(4)	48	H9A	C9	H9B	109.5
21	H4A	C4	H4B	108	49	H9A	C9	H9C	109.5
22	H4A	C4	C5	109.4	50	H9B	C9	H9C	109.5
23	H4B	C4	C5	109.5	51	F11	B10	F12	110.1(5)
24	C4	C5	H5A	109.6	52	F11	B10	F13	108.7(5)
25	C4	C5	H5B	109.6	53	F11	B10	F14	109.2(5)
26	C4	C5	C6	110.3(4)	54	F12	B10	F13	111.3(5)
27	H5A	C5	H5B	108.1	55	F12	B10	F14	109.2(5)
28	H5A	C5	C6	109.6	56	F13	B10	F14	108.3(5)

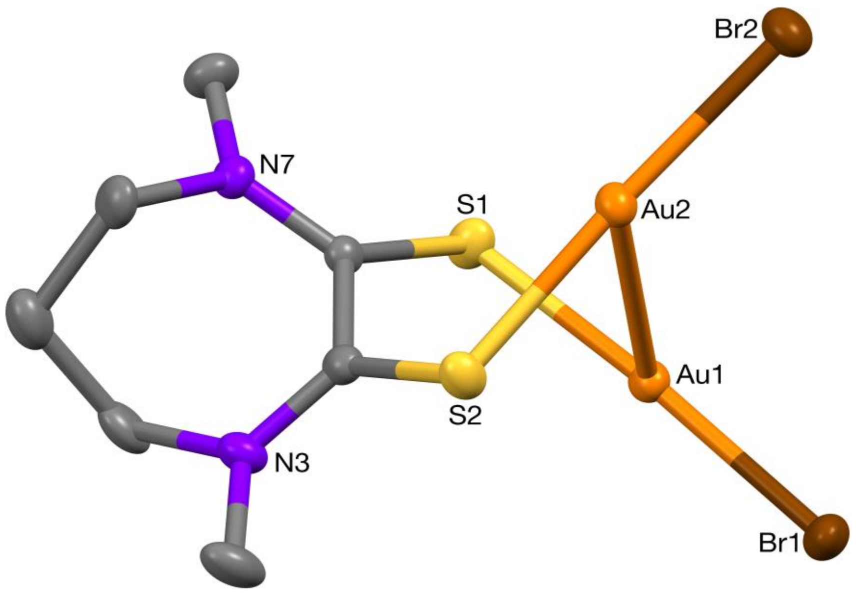

Figure S9. The X-ray crystal structure of **[Me$_2$dazdt(AuBr)$_2$]** (thermal ellipsoids drawn at 50% probability).

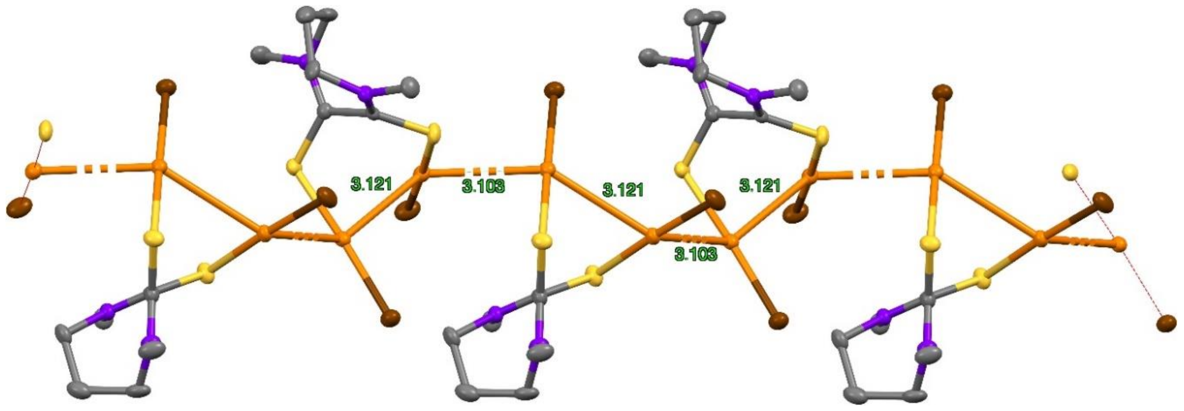

Figure S10. The X-ray crystal structure of **[Me$_2$dazdt(AuBr)$_2$]** illustrating chain of molecules formed along b axis. (thermal ellipsoids drawn at 50% probability).

Crystal data for **[Me$_2$dazdt(AuBr)$_2$]**: [$C_7H_{12}Au_2Br_2N_2S_2$], M = 742.06, monoclinic, $P2_1/n$ (no. 14), a = 9.8393(3), b = 10.7280(3), c = 13.4277(3) Å, β = 104.018(3)°, V = 1375.16(6) Å^3, Z = 4, D_c = 3.584 g cm^{-3}, μ(Mo-Kα) = 27.398 mm^{-1}, T = 173 K, red blocks, Agilent Xcalibur 3 E diffractometer; 3214 independent measured reflections (R_{int} = 0.0469), F^2 refinement,[S7,S8] R_1(obs) = 0.0212, wR_2(all) = 0.0434, 2841 independent observed absorption-corrected reflections [$|F_o| > 4\sigma(|F_o|)$, completeness to θ_{full}(25.2°) = 99.9%], 139 parameters. CCDC 2156496.

Short intermolecular Au…Au contacts (3.103 Å) were observed for [Me$_2$dazdt(AuBr)$_2$] with distances comparable to the intramolecular contacts (3.121 Å), resulting in the formation of a chain of molecules along the direction of the b axis (Figure S10).

Table S8. Table of **[Me_2dazdt$(AuBr)_2$]** bond lengths.

Number	Atom1	Atom2	Polymeric	Cyclicity	Length
1	Au1	Au2	no	cyclic	3.1209(3)
2	Au1	Br1	no	acyclic	2.3856(6)
3	Au1	S1	no	cyclic	2.273(1)
4	Au1	Au2	yes	acyclic	3.1032(3)
5	Au2	Br2	no	acyclic	2.3920(6)
6	Au2	S2	no	cyclic	2.275(1)
7	Au2	Au1	yes	acyclic	3.1032(3)
8	S1	C1	no	cyclic	1.686(5)
9	C1	C2	no	cyclic	1.500(6)
10	C1	N7	no	cyclic	1.310(5)
11	C2	S2	no	cyclic	1.689(5)
12	C2	N3	no	cyclic	1.315(7)
13	N3	C4	no	cyclic	1.470(6)
14	N3	C8	no	acyclic	1.461(7)
15	C4	H4A	no	acyclic	0.99
16	C4	H4B	no	acyclic	0.99
17	C4	C5	no	cyclic	1.515(7)
18	C5	H5A	no	acyclic	0.99
19	C5	H5B	no	acyclic	0.99
20	C5	C6	no	cyclic	1.516(9)
21	C6	H6A	no	acyclic	0.99
22	C6	H6B	no	acyclic	0.99
23	C6	N7	no	cyclic	1.482(7)
24	N7	C9	no	acyclic	1.465(6)
25	C8	H8A	no	acyclic	0.98
26	C8	H8B	no	acyclic	0.979
27	C8	H8C	no	acyclic	0.98
28	C9	H9A	no	acyclic	0.98
29	C9	H9B	no	acyclic	0.98
30	C9	H9C	no	acyclic	0.98

Table S9. Table of **[Me_2dazdt$(AuBr)_2$]** bond angles.

Number	Atom1	Atom2	Atom3	Angle	Number	Atom1	Atom2	Atom3	Angle
1	Au2	Au1	Br1	103.93(2)	29	H4B	C4	C5	109.5
2	Au2	Au1	S1	77.90(3)	30	C4	C5	H5A	109.5
3	Au2	Au1	Au2	131.37(1)	31	C4	C5	H5B	109.5
4	Br1	Au1	S1	176.35(4)	32	C4	C5	C6	110.8(4)
5	Br1	Au1	Au2	97.83(2)	33	H5A	C5	H5B	108.1
6	S1	Au1	Au2	83.08(3)	34	H5A	C5	C6	109.5
7	Au1	Au2	Br2	106.41(2)	35	H5B	C5	C6	109.5
8	Au1	Au2	S2	74.42(3)	36	C5	C6	H6A	109.6
9	Au1	Au2	Au1	145.20(1)	37	C5	C6	H6B	109.6
10	Br2	Au2	S2	176.34(4)	38	C5	C6	N7	110.2(4)
11	Br2	Au2	Au1	96.68(2)	39	H6A	C6	H6B	108.1
12	S2	Au2	Au1	84.20(3)	40	H6A	C6	N7	109.7
13	Au1	S1	C1	107.6(2)	41	H6B	C6	N7	109.6
14	S1	C1	C2	121.6(3)	42	C1	N7	C6	119.9(4)
15	S1	C1	N7	122.1(4)	43	C1	N7	C9	122.3(4)
16	C2	C1	N7	116.0(4)	44	C6	N7	C9	117.2(4)
17	C1	C2	S2	122.7(4)	45	N3	C8	H8A	109.4
18	C1	C2	N3	115.6(4)	46	N3	C8	H8B	109.5
19	S2	C2	N3	121.7(4)	47	N3	C8	H8C	109.4
20	Au2	S2	C2	106.7(2)	48	H8A	C8	H8B	109.5
21	C2	N3	C4	120.2(4)	49	H8A	C8	H8C	109.5
22	C2	N3	C8	120.1(4)	50	H8B	C8	H8C	109.5
23	C4	N3	C8	118.7(4)	51	N7	C9	H9A	109.4
24	N3	C4	H4A	109.5	52	N7	C9	H9B	109.5
25	N3	C4	H4B	109.5	53	N7	C9	H9C	109.4
26	N3	C4	C5	110.7(4)	54	H9A	C9	H9B	109.5
27	H4A	C4	H4B	108.1	55	H9A	C9	H9C	109.5
28	H4A	C4	C5	109.5	56	H9B	C9	H9C	109.5

1.6. Preliminary indicative cost analysis

Preliminary indicative cost analysis

The gold recycling process employed in this work[S4] can be applied to different kinds of WEEE with costs that will vary depending on the configuration of the industrial plant. However, this report demonstrates the use of the gold complexes produced from this process in catalysis, illustrating the potential of joining up the recovery and catalysis processes using the example of SIM cards as the WEEE feedstock. In order to provide a preliminary indicative cost comparison with the cheapest commercial catalyst used for the same catalytic transformations, a calculation has been performed of the cost to prepare the catalyst $[AuI_2(Me_2dazdt)]I_3$ (**1a**) from used SIM cards based on small-scale reagent pricing by the major suppliers. A more meaningful cost analysis will need and be performed referring to a higher technology readiness level (TRL) plant than the one here.

The preliminary indicative cost below reveals that, depending on whether the cost of SIM cards is included, the same amount of **1a** is 29-55% less than that of commercial $AuCl_3$ (the cheapest of the literature catalysts used in the research). It is often estimated that the scale up of a process reduces costs ten-fold so the costs above for the preparation of **1a** would be reduced dramatically on scale up. In comparison, the cost of commercial $AuCl_3$ is already minimized through the savings of large-scale production. This estimate shows that, even unoptimized, small-scale production of catalyst **1a** leads to a significantly lower cost than commercial catalysts derived from environmentally-damaging mining.

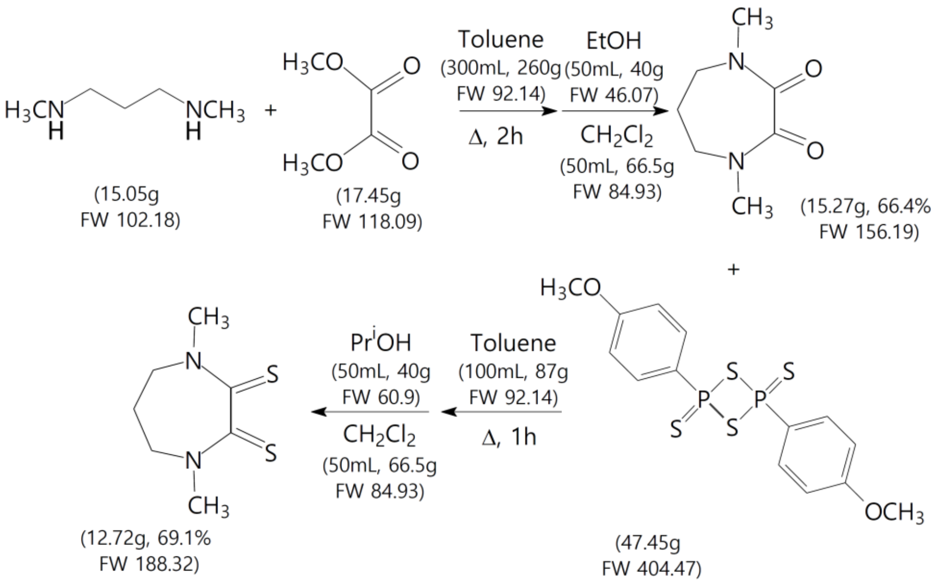

Scheme S17. Synthesis of Me_2dazdt.[S2,S4]

Table S10. Amounts of reagents and solvents with associated costs for the synthesis of Me_2dazdt·and the iodine used to make $Me_2dazdt·2I_2$ *in situ* (prices from Merck).

Chemicals	Amount	Chemicals cost (per unit amount)	Overall cost of Chemicals
N,*N'*-dimethyl-1,3-propanediamine	15.05 g	€ 15.98 per g	€ 240.50
Dimethyloxalate	17.45 g	€ 0.12 per g	€ 2.09
Toluene	400 mL	€ 19.89 per L	€ 7.96
Ethanol	50 mL	€ 21.37 per L	€ 1.07
Dichloromethane	10 mL	€ 17.60 per L	€ 1.76
Isopropanol	50 mL	€ 5.30 per L	€ 0.27
Lawesson's reagent	47.45 g	€ 1.50 per g	€ 71.18
Iodine (to make $Me_2dazdt·2I_2$)	68.57 g	€ 0.30 per g	€ 20.57
**Total product**	**81.29 g**	**€ 4.25 ($ 4.25) per g**	**€ 345.40**

Energy consumption has been considered but not included, as this is difficult to calculate. However, an estimation based on the literature[S9] suggests that values around 2 kWh would be needed (mainly due to 3h refluxing in toluene). The average cost of one kWh over the last 7 years[S10] is € 0.08, rendering the energy cost a negligible **€ 0.16 ($ 0.16)**.

Using the cost of preparing Me_2dazdt and its use in situ with iodine (as $Me_2dazdt·2I_2$), the cost of synthesizing the catalyst $[AuI_2(Me_2dazdt)]I_3$ (**1a**) can be calculated:

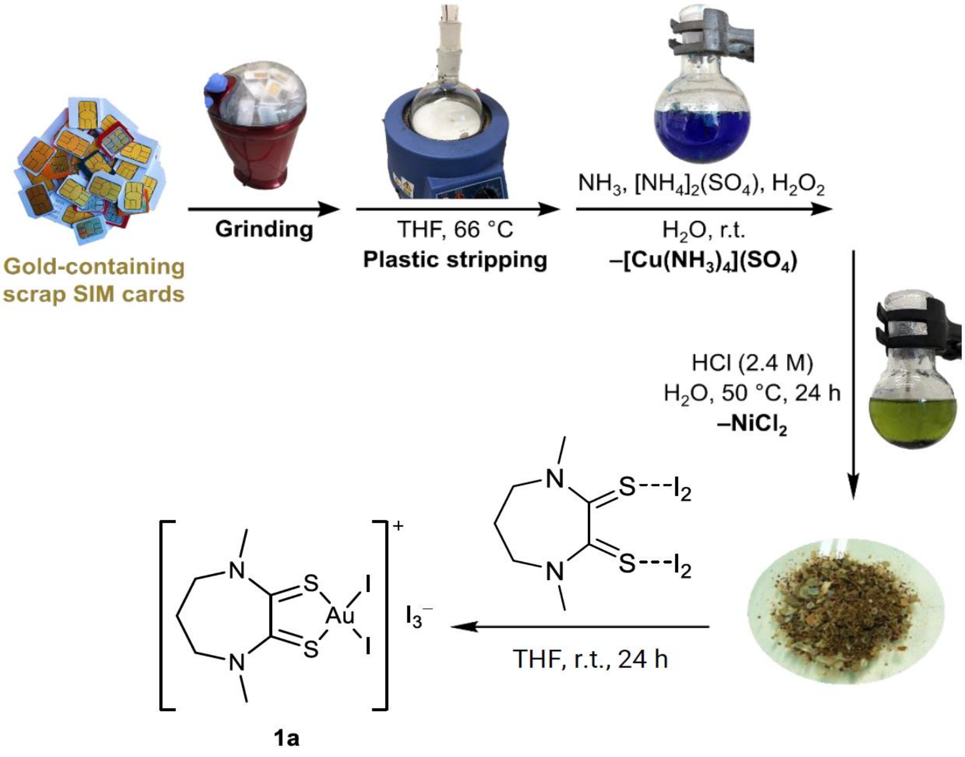

Scheme S18. Synthesis of $[AuI_2(Me_2dazdt)]I_3$ (**1a**) see Section 1.2 for experimental details.

Table S11. Amounts of reagents and solvents with associated costs for the synthesis of $[AuI_2(Me_2dazdt)]I_3$ (**1a**) using prices from Merck apart from for $Me_2dazdt{\cdot}2I_2$ (prepared as above) and comparison with the same amount of commercial $AuCl_3$ catalyst.

Chemicals	Amount in mL/mg	Amount (mmol)	Chemicals cost (per unit)	Overall cost of Chemicals
Tetrahydrofuran	150 mL		€ 5.02 per L	€ 0.75
32% NH_4OH	10 mL		€ 3.60 per L	€ 0.04
Ammonium sulfate	2200 mg		€ 0.02 per g	€ 0.05
30% H_2O_2	3 mL		€ 4.46 per L	€ 0.01
37% HCl	4.2 mL		€ 3.44 per L	€ 0.02
Et_2O	25 mL		€ 5.70 per L	€ 0.14
$Me_2dazdt{\cdot}I_2$	361 mg		**€ 4.25 per g**	€ 1.53
Total of **1a** (M_w = 1019.81) obtained	130 mg	0.128	€ 19.54 per g	**€ 2.54 ($2.54)**
$AuCl_3$ catalyst (M_w = 303.33)	39 mg	0.128	€ 145.83 per g	**€ 5.69 ($5.69)**

The estimations above suggest that the cost of **1a** is less than half that of commercial $AuCl_3$, which is the cheapest of the literature catalysts used in the research. Additional value generated by valorization of $[Cu(NH_3)_4](SO_4)$ and $NiCl_2$ produced from the SIM cards is not included.

Thirty SIM cards were used to obtain 130 mg of **1a**. 1700 used SIM cards are available on ebay for €85 (€0.05 each so 30 would cost €1.50). On scale up, it is anticipated that the cost of used SIM cards would be far lower than €0.05 per card and so this cost would become negligible. Even when including the cost of SIM cars purchased for the purpose of this research, the cost of **1a** is 29% less than that of commercial $AuCl_3$.

It is often estimated that the scale up of a process would reduce the cost ten-fold so the costs above for the preparation of **1a** would be reduced dramatically on scale up. In comparison, the cost of commercial $AuCl_3$ is already minimized through the savings of large-scale production.

1.7. References

S1 Isaksson, R.; Liljefors, T.; Sandstrom, J., *J. Chem. Res.*, Miniprint 1981, 664

S2 Bigoli, F.; Deplano, P.; Mercuri, M. L.; Pellinghelli, M. A.; Pintus, G.; Serpe, A.; Trogu, E. F. A Powerful New Oxidation Agent towards Metallic Gold Powder: *N,N′*-Dimethylperhydrodiazepine-2,3-Dithione (D) Bis(Diiodine). Synthesis and X-Ray Structure of $[AuDI_2]I_3$. *Chem. Commun.* **1998**, *484*, 2351–2352 (DOI: 10.1039/a806158k).

S3 Cau, L.; Deplano, P.; Marchiò, L.; Mercuri, M. L.; Pilia, L.; Serpe, A.; Trogu, E. F. New Powerful Reagents Based on Dihalogen/*N,N′*-Dimethylperhydrodiazepine-2,3-Dithione Adducts for Gold Dissolution: The IBr Case. *Dalton Trans.* **2003**, *10*, 1969–1974 (DOI: 10.1039/B210281A).

S4 Serpe, A.; Deplano, P.; Mercuri, M. L.; Pilia, L.; Vanzi, M. Process for Recovering Noble Metals from Electric and Electronic Wastes. EP1964936B1, 2008.

S5 Braddock, D. C.; Lickiss, P. D.; Rowley, B. C.; Pugh, D.; Purnomo, T.; Santhakumar, G.; Fussell, S. J. Tetramethyl Orthosilicate (TMOS) as a Reagent for Direct Amidation of Carboxylic Acids. *Org. Lett*. **2018**, *20*, 950–953 (DOI: 10.1021/acs.orglett.7b03841).

S6 Derosa, J.; Cantu, A. L.; Boulous, M. N.; O'Duill, M. L.; Turnbull, J. L.; Liu, Z.; De La Torre, D. M.; Engle, K. M. Palladium(II)-Catalyzed Directed Anti-Hydrochlorination of Unactivated Alkynes with HCl. *J. Am. Chem. Soc*. **2017**, *139*, 5183–5193 (DOI: 10.1021/jacs.7b00892).

S7 SHELXTL v5.1, Bruker AXS, Madison, WI, 1998.

S8 SHELX-2013, G.M. Sheldrick, *Acta Cryst.* **2015**, *C71*, 3-8 (DOI: 10.1107/S2053273314026370).

S9 Integrating Green and Sustainable Chemistry Principles into Education, Elsevier 2019, Eds. A. Dicks, L. Bastin (ISBN: 9780128174180).

S10 https://www.statista.com/statistics/589765/average-electricity-prices-uk/

2. NMR Spectra

2.1. ^{1}H NMR Spectra

[AuI$_2$(Me$_2$dazdt)]BF$_4$ (1b)

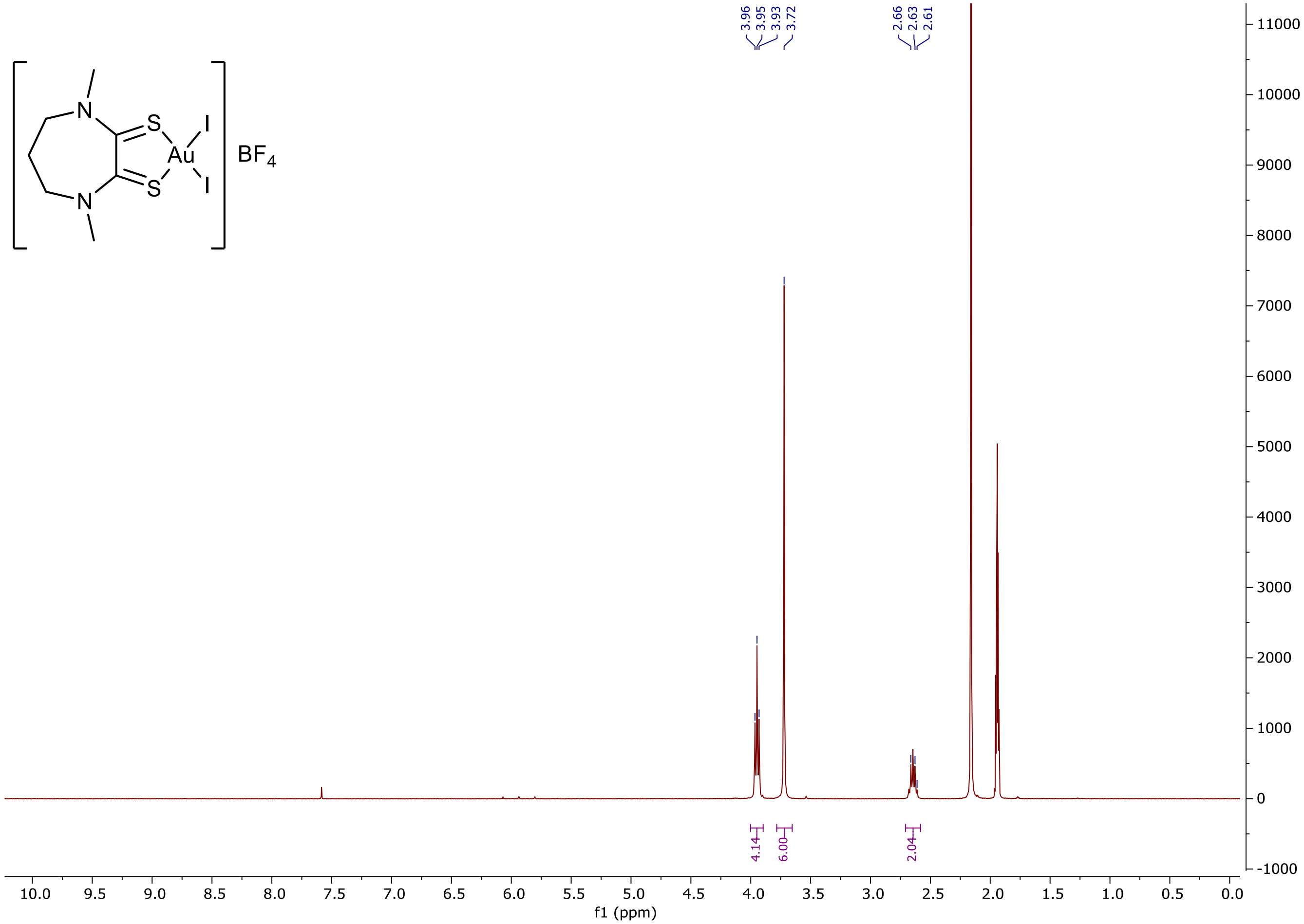

Figure S11. ^{1}H NMR (400 MHz, Acetonitrile-d_3) spectrum of [AuI$_2$(Me$_2$dazdt)]BF$_4$ (**1b**).

[AuBr$_2$(Me$_2$dazdt)]BF$_4$ (2b)

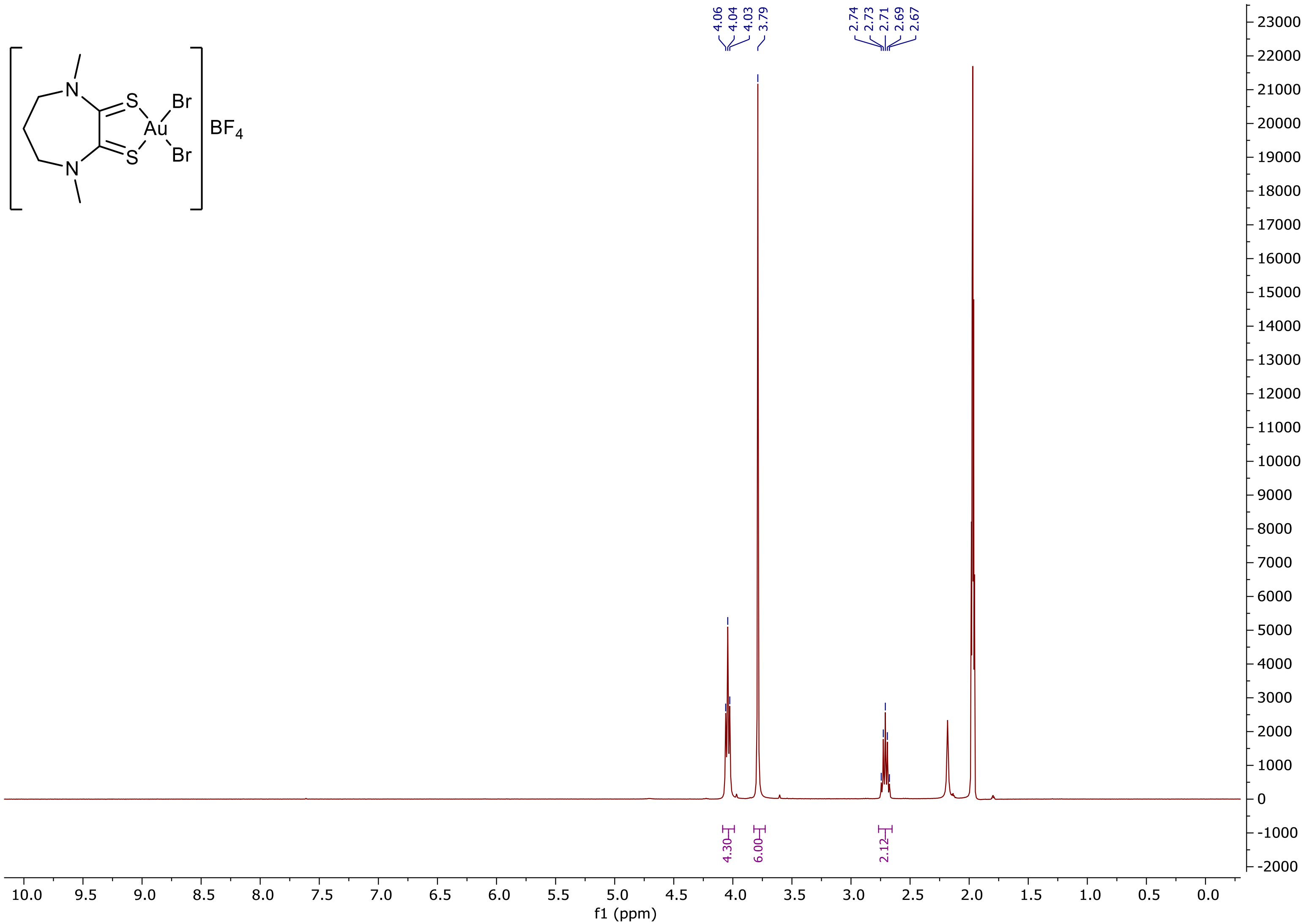

Figure S12. ^{1}H NMR (400 MHz, Acetonitrile-d_3) spectrum of [AuBr$_2$(Me$_2$dazdt)]BF$_4$ (**2b**).

***N*-(Prop-2-yn-1-yl)benzamide**

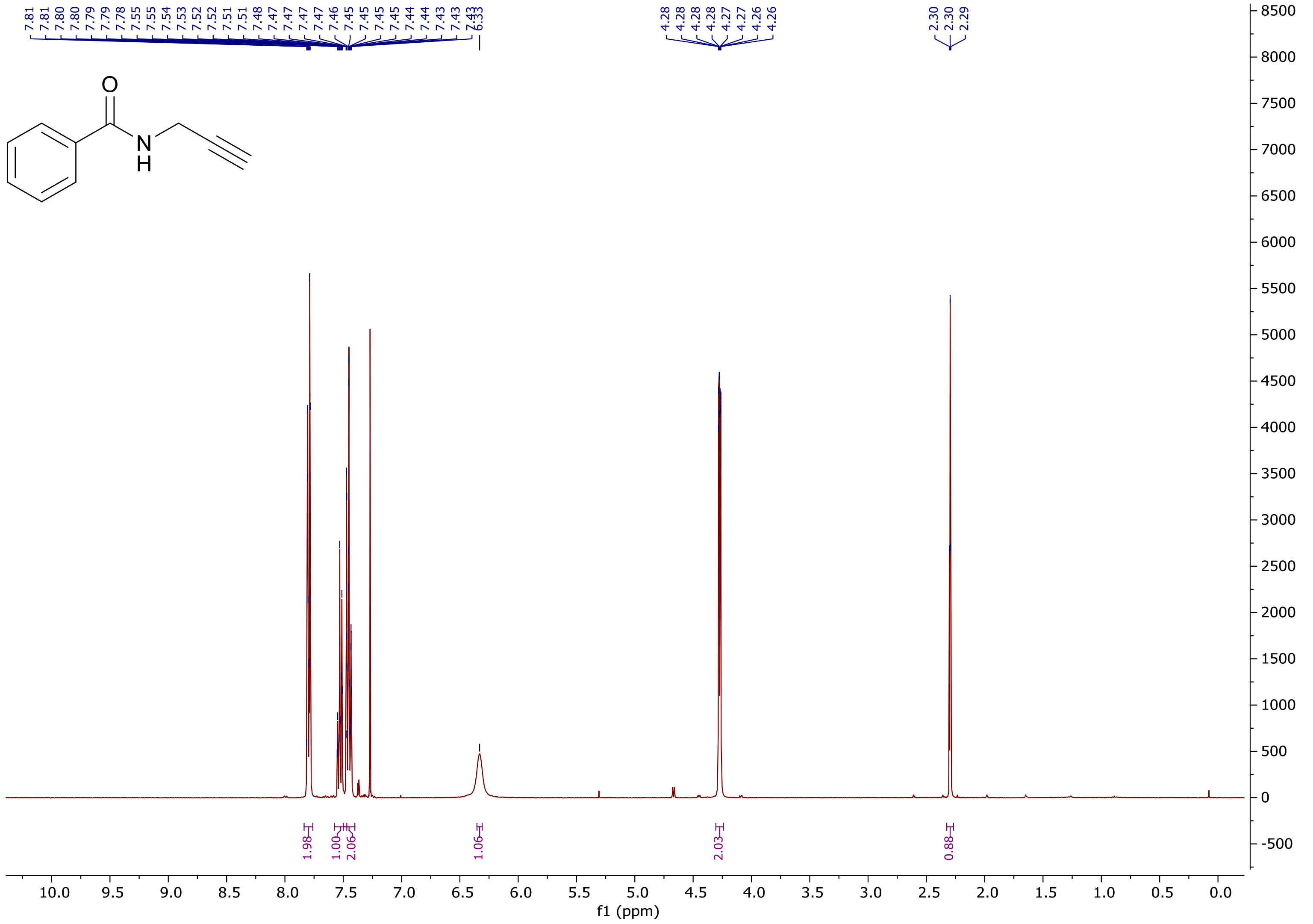

Figure S13. ^{1}H NMR (400 MHz, Chloroform-*d*) spectrum of *N-(prop-2-yn-1-yl)benzamide*.

N-(3-Phenylprop-2-yn-1-yl)benzamide

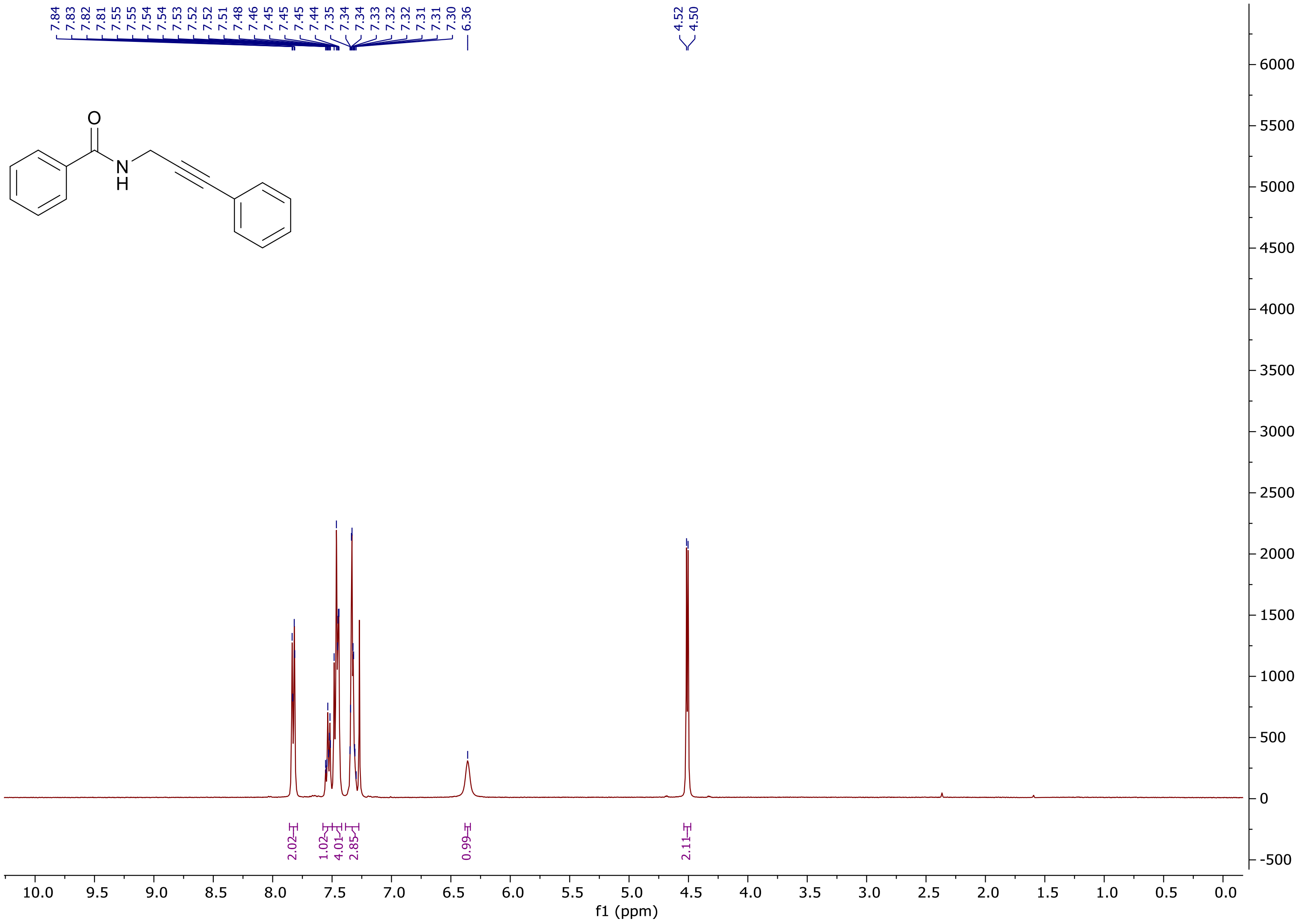

Figure S14. ^{1}H NMR (400 MHz, Chloroform-*d*) spectrum of *N-(3-phenylprop-2-yn-1-yl)benzamide*.

5-Methyl-2-phenyloxazole

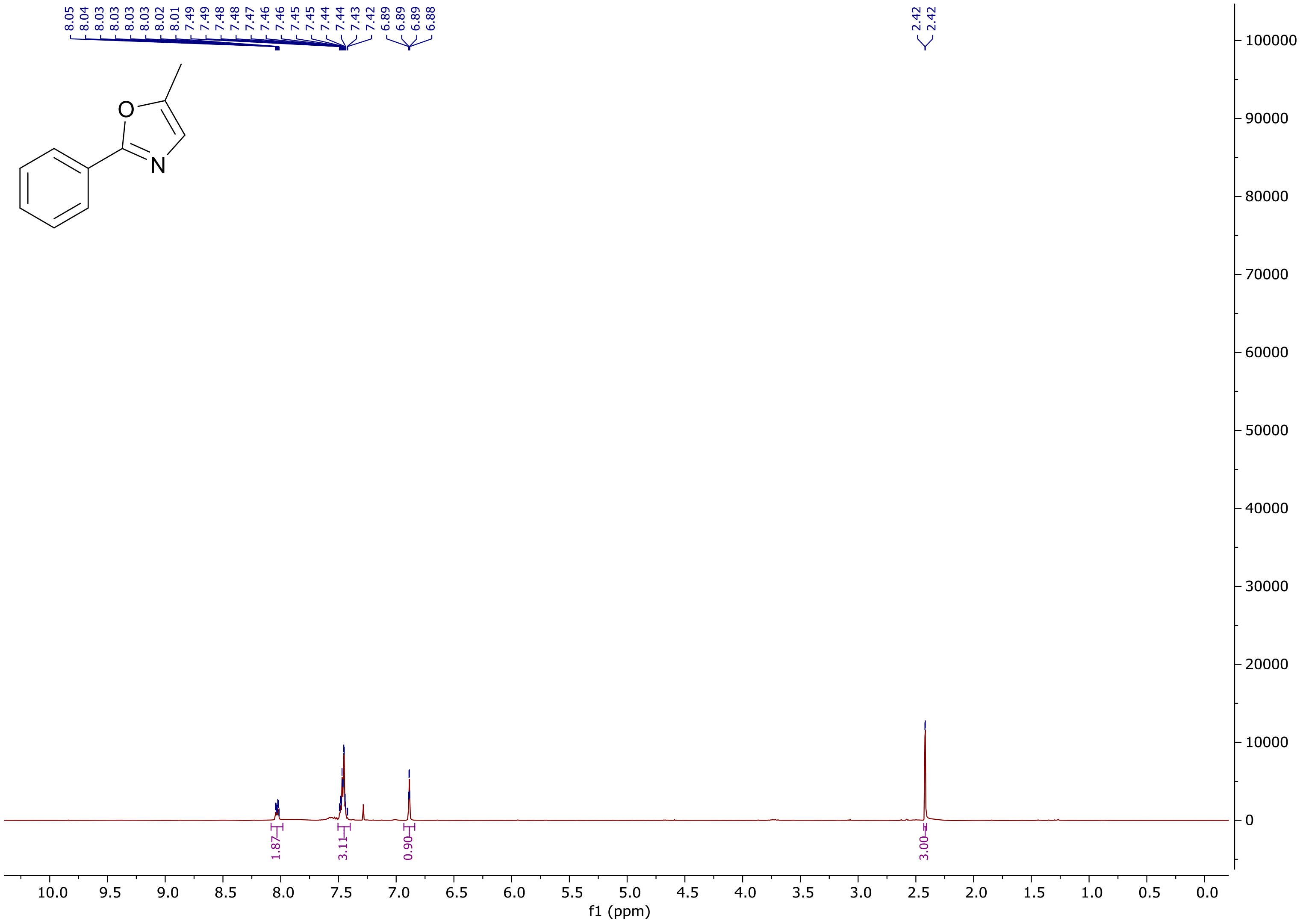

Figure S15. ^{1}H NMR (400 MHz, Chloroform-*d*) spectrum of *5-methyl-2-phenyloxazole*.

(Z)-5-Benzylidene-2-phenyl-4,5-dihydrooxazole

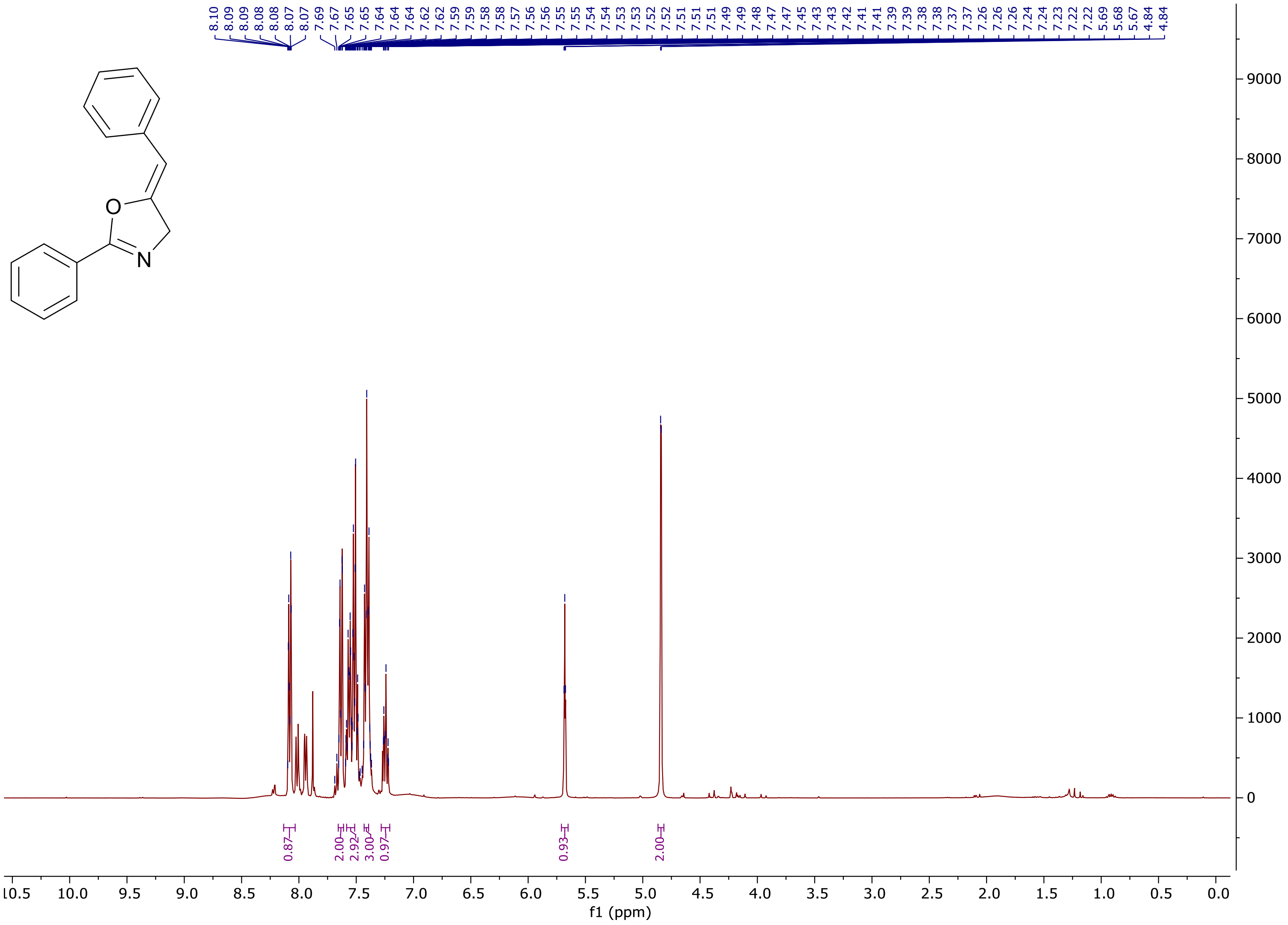

Figure S16. ^{1}H NMR (400 MHz, Chloroform-*d*) spectrum of *(Z)-5-benzylidene-2-phenyl-4,5-dihydrooxazole*

(E)-5-(Iodo(phenyl)methylene)-2-phenyl-4,5-dihydrooxazole

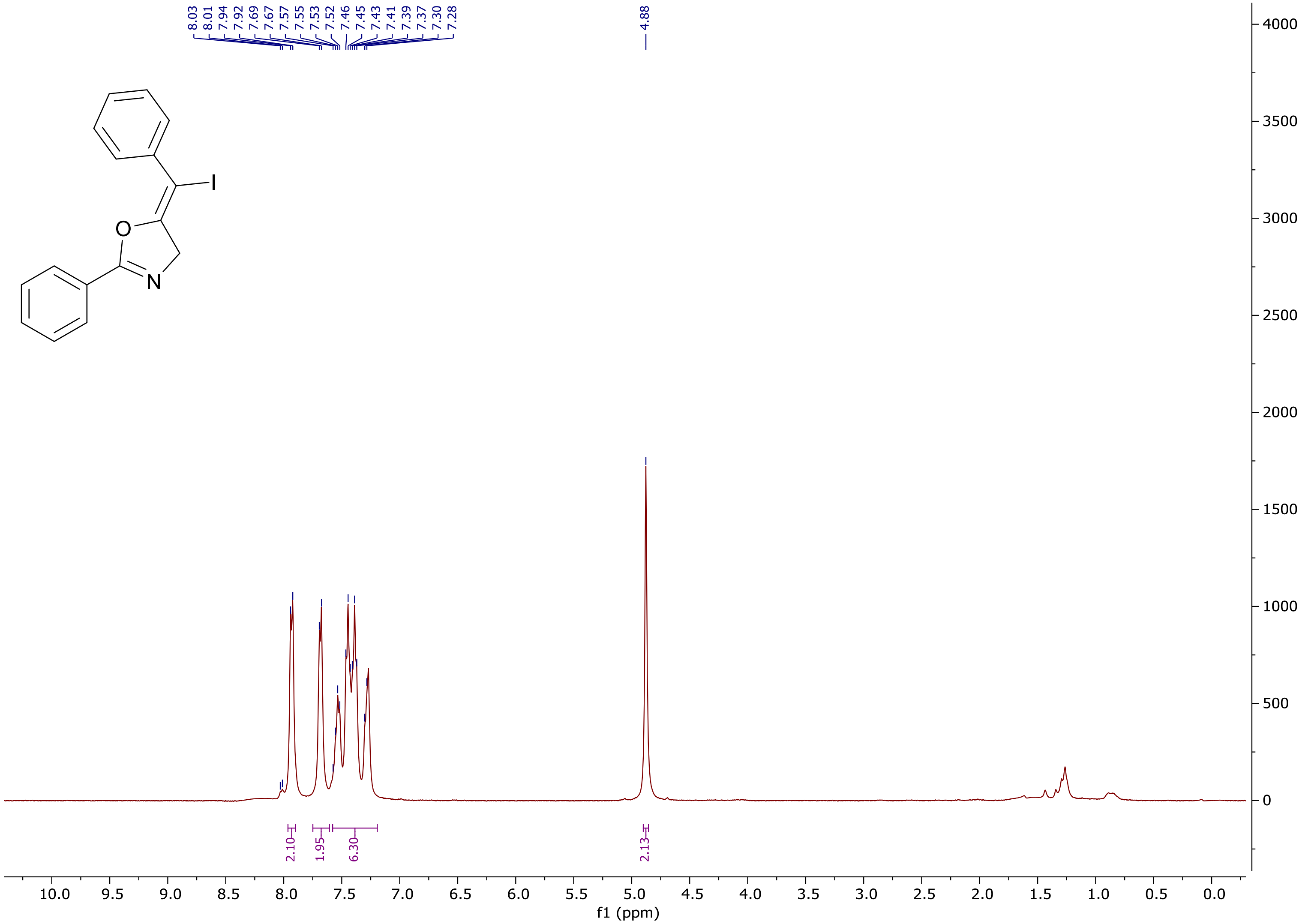

Figure S17. ^{1}H NMR (400 MHz, Chloroform-*d*) spectrum of *(E)-5-(iodo(phenyl)methylene)-2-phenyl-4,5-dihydrooxazole*.

Condensation of *o*-iodoaniline and acetylacetone

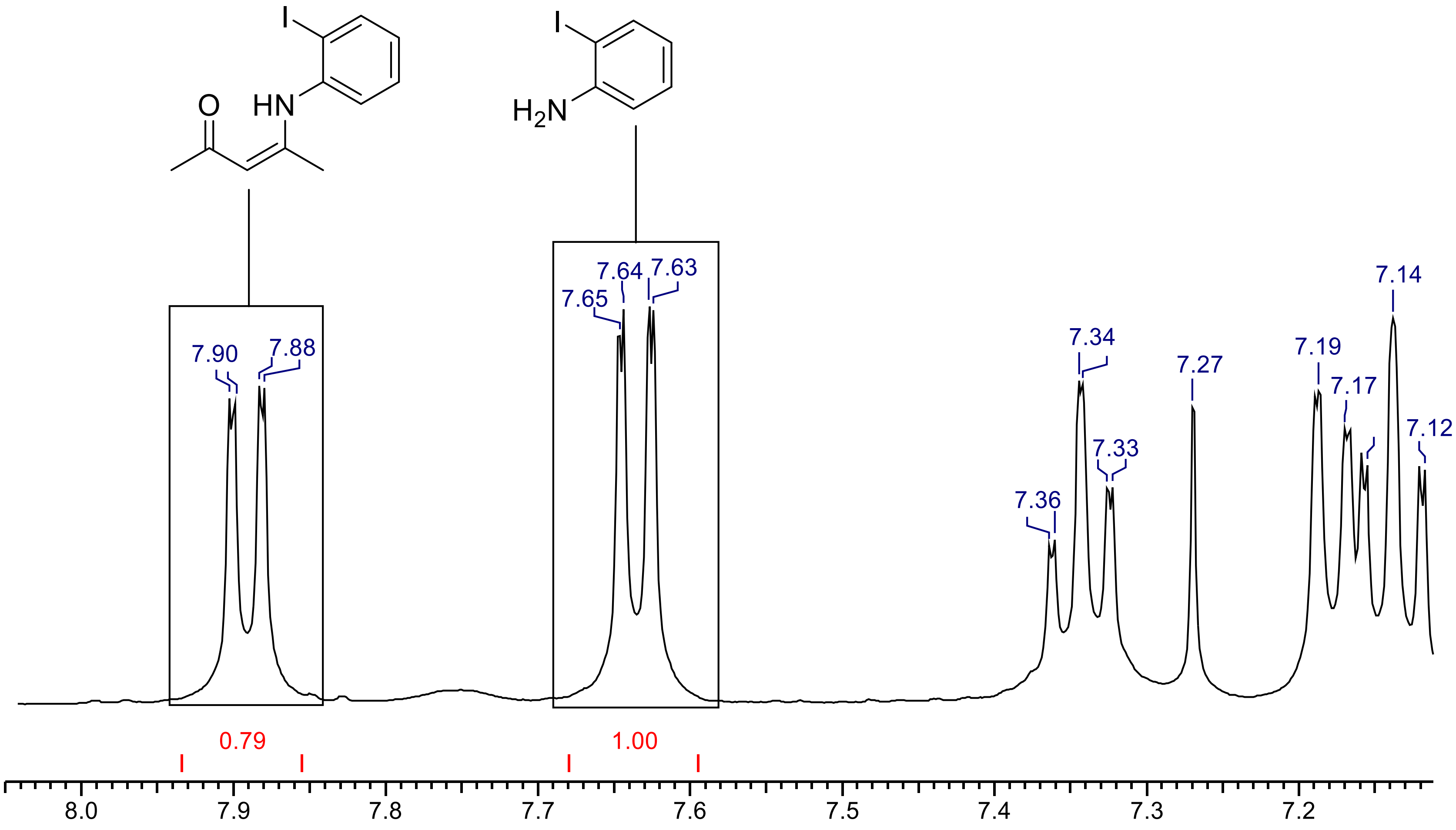

Figure S18. Example ^{1}H NMR (400 MHz, Chloroform-*d*) spectrum of a crude *o*-iodoaniline and acetylacetone condensation reaction mixture illustrating reaction monitoring.

Addition of 1,3-dimethoxybenzene to methyl vinyl ketone.

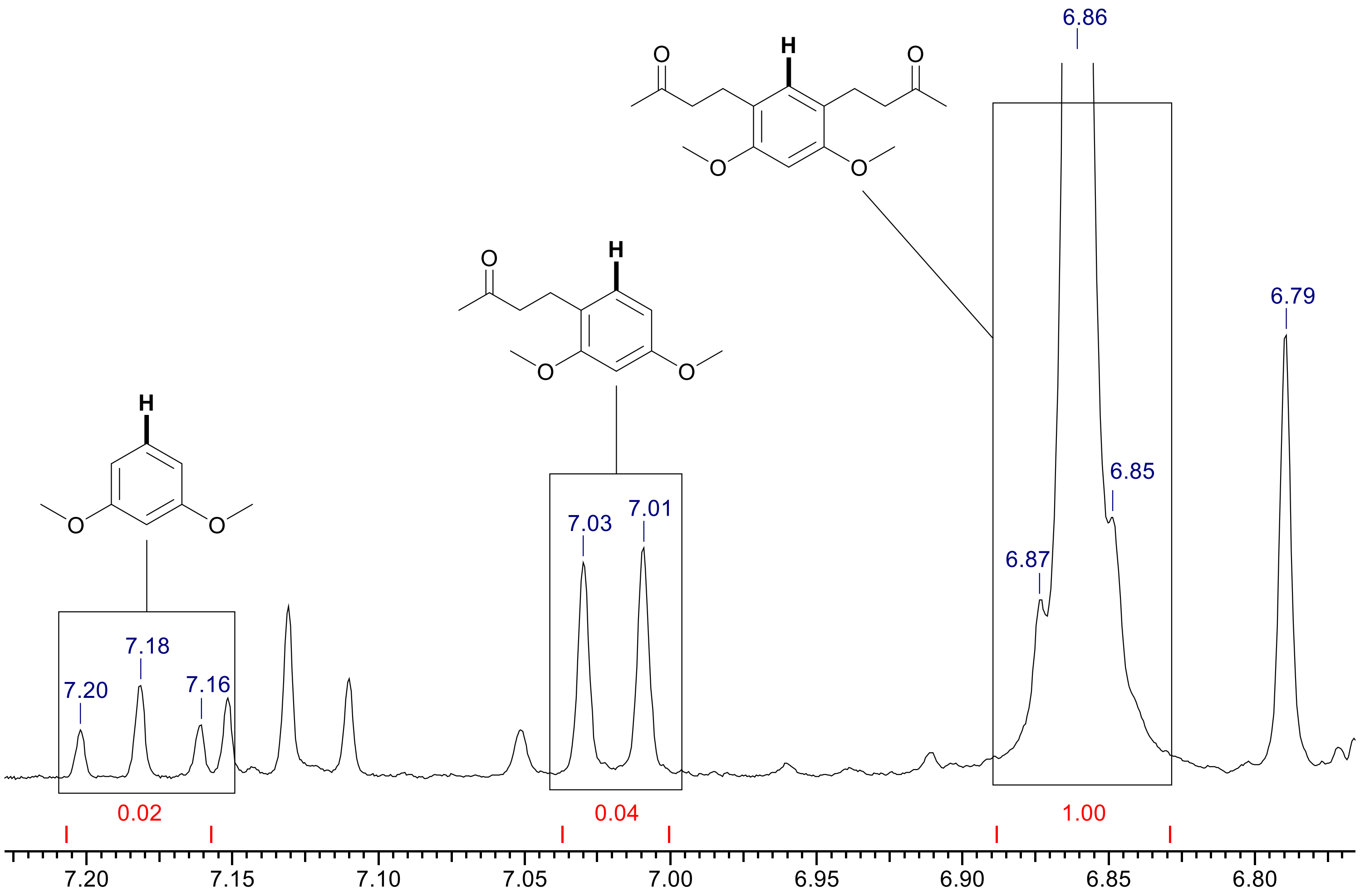

Figure S19. Example ^{1}H NMR (400 MHz, Chloroform-*d*) spectrum of the crude reaction mixture from the addition of 1,3-dimethoxybenzene to methyl vinyl ketone illustrating reaction monitoring.

4,4'-(Azulene-1,3-diyl)bis(butan-2-one)2

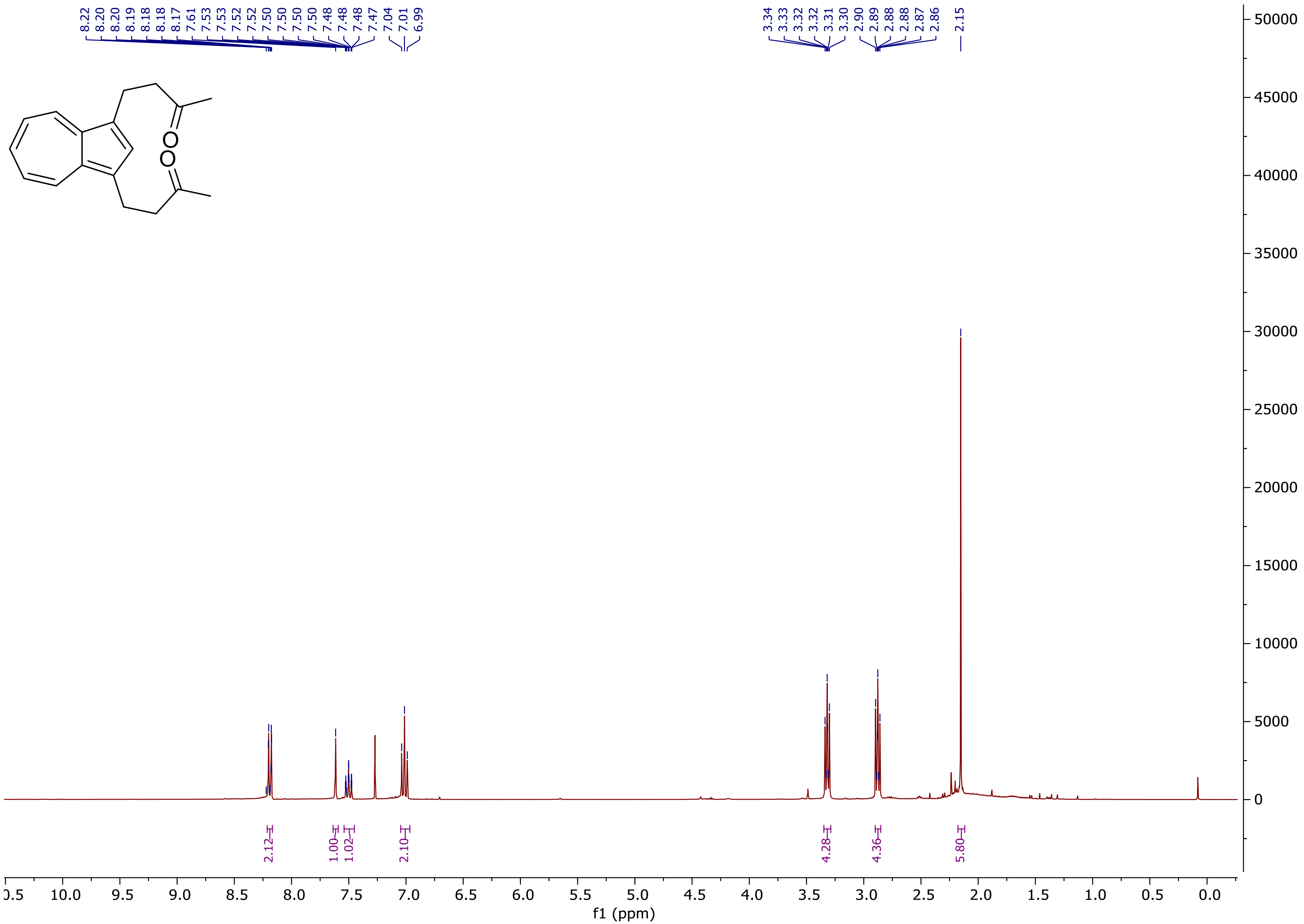

Figure S20. ^{1}H NMR (400 MHz, Chloroform-*d*) spectrum of *4,4'-(azulene-1,3-diyl)bis(butan-2-one)2*.

4-(5-Methylfuran-2-yl)butan-2-one

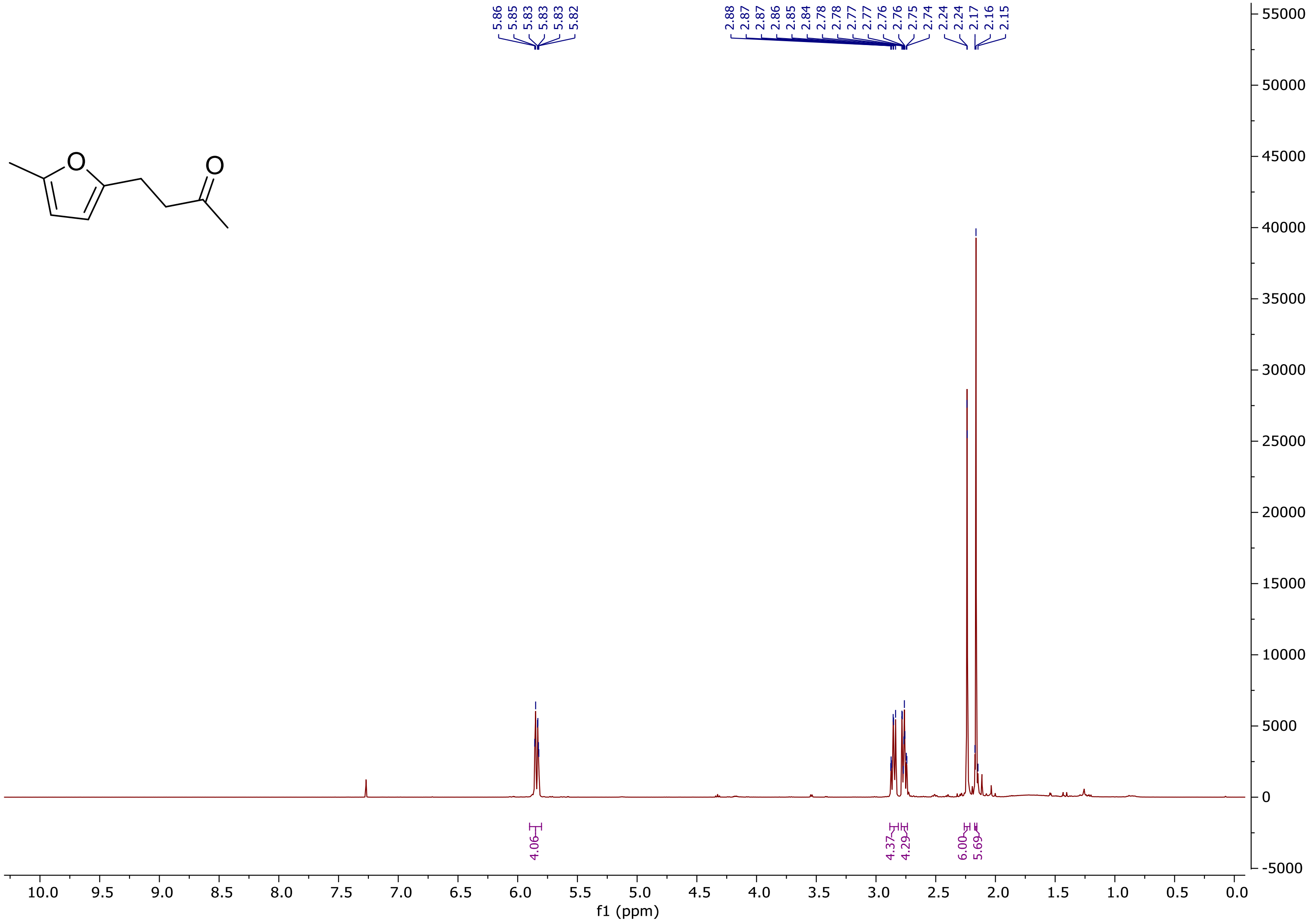

Figure S21. ^{1}H NMR (400 MHz, Chloroform-*d*) spectrum of *4-(5-methylfuran-2-yl)butan-2-one.*

3-(5-Methylfuran-2-yl)cyclohexan-1-one

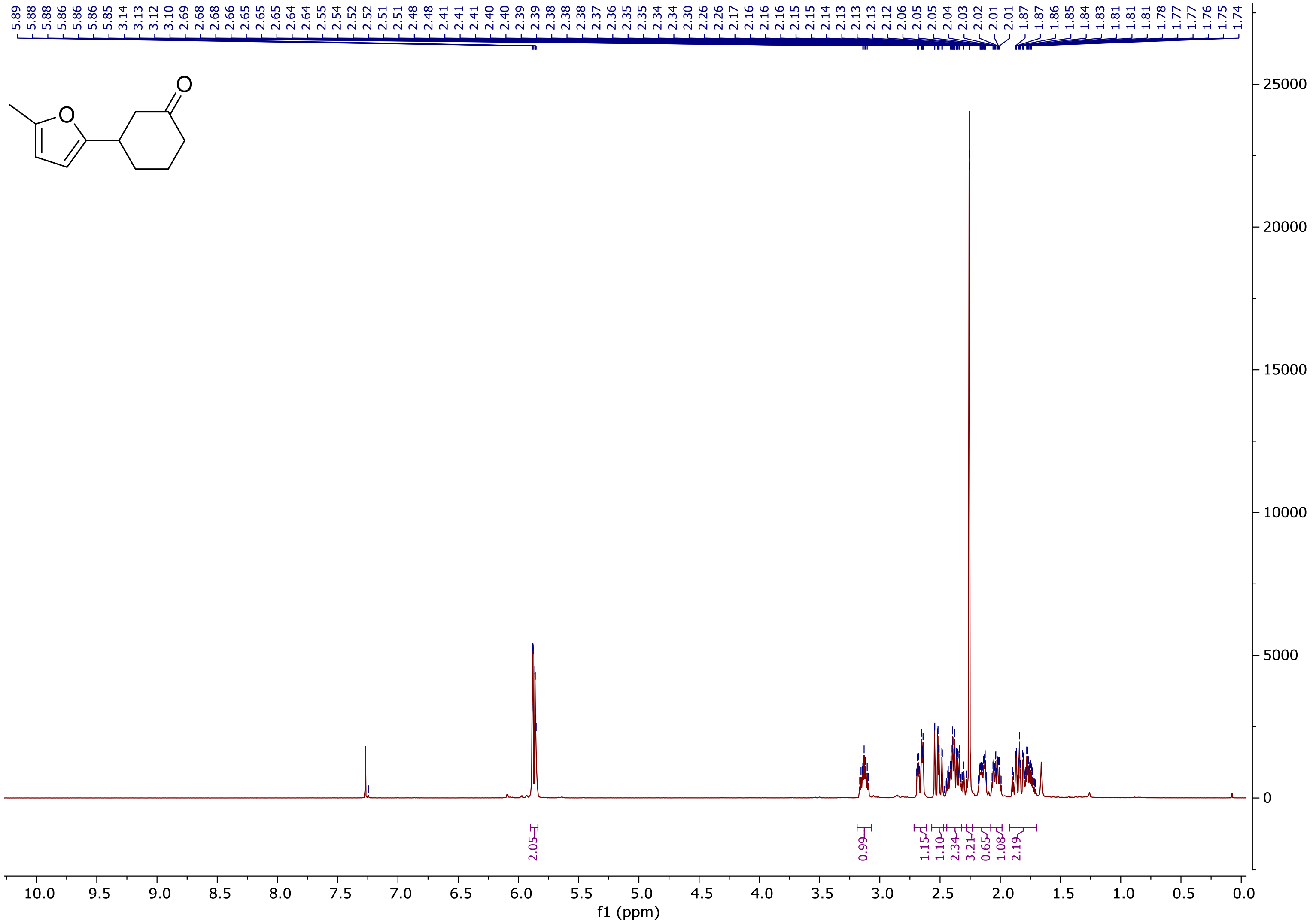

Figure S22. ^{1}H NMR (400 MHz, Chloroform-*d*) spectrum of *3-(5-methylfuran-2-yl)cyclohexan-1-one*.

3-Chloro-4-methoxy-1,1'-biphenyl

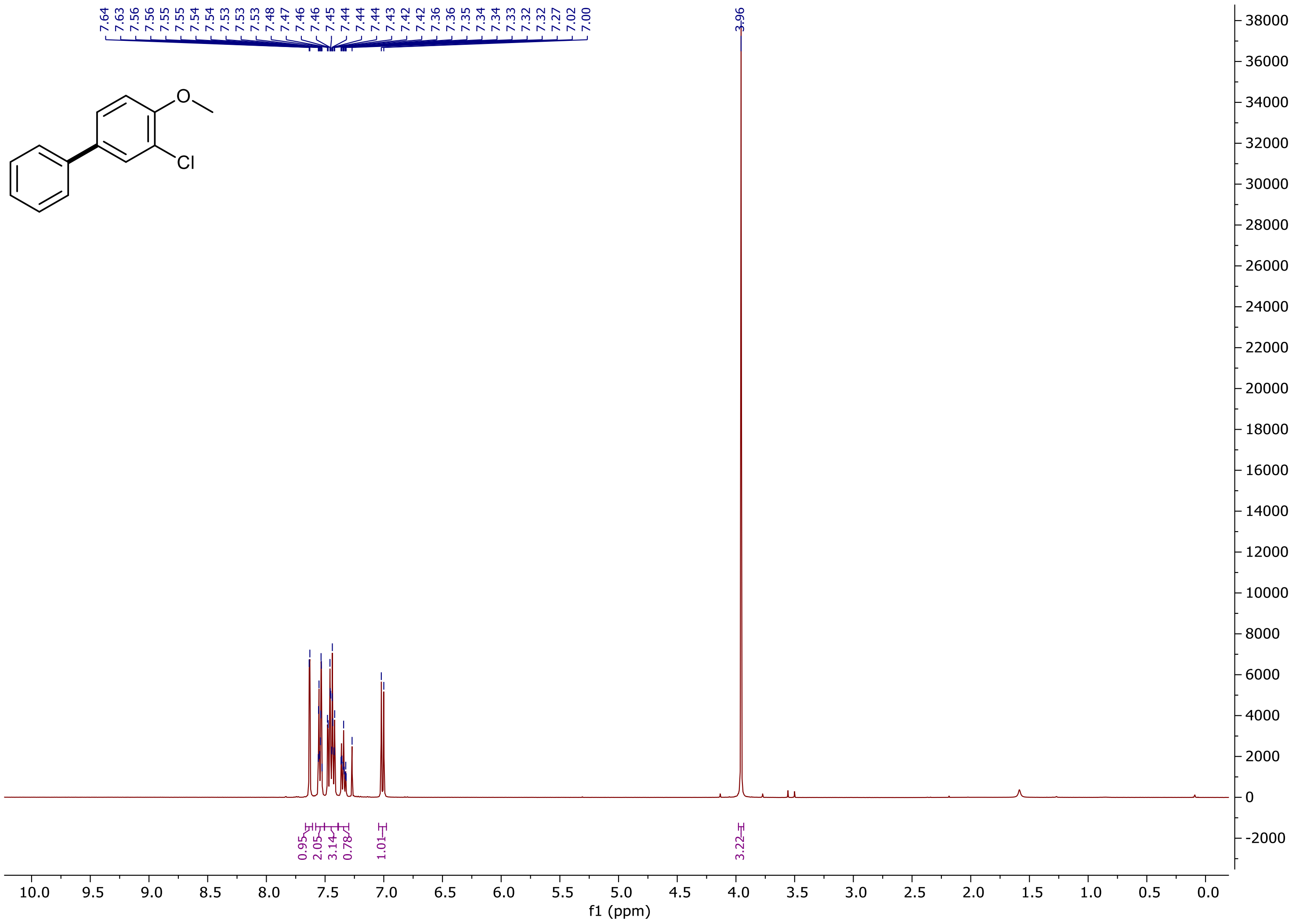

Figure S23. ^{1}H NMR (400 MHz, Chloroform-*d*) spectrum of *3-chloro-4-methoxy-1,1'-biphenyl.*

3-Bromo-4-methoxy-1,1'-biphenyl

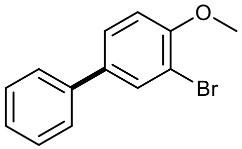

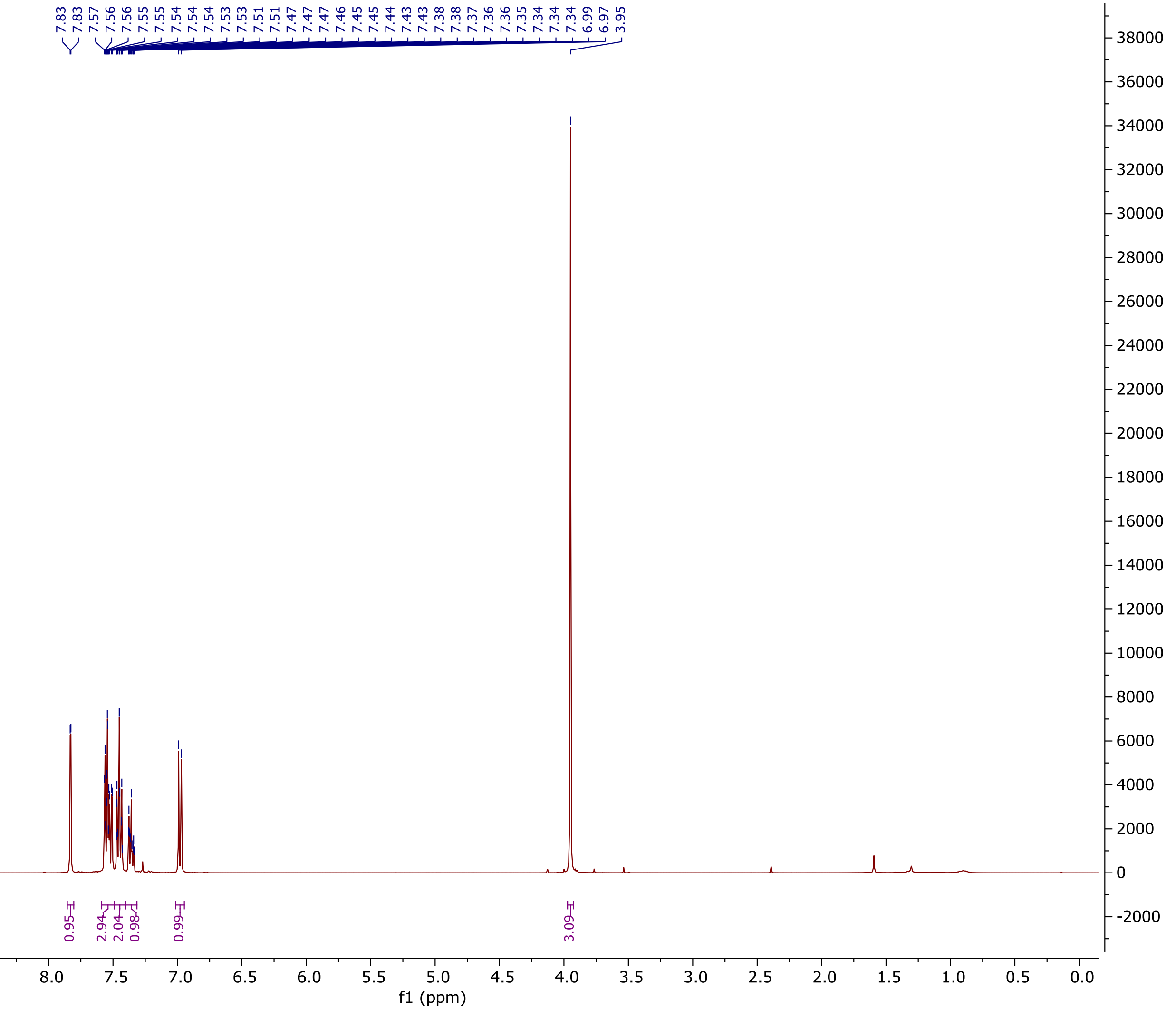

Figure S24. ^{1}H NMR (400 MHz, Chloroform-*d*) spectrum of *3-bromo-4-methoxy-1,1'-biphenyl.*

3,4-Dimethyl-1,1'-biphenyl

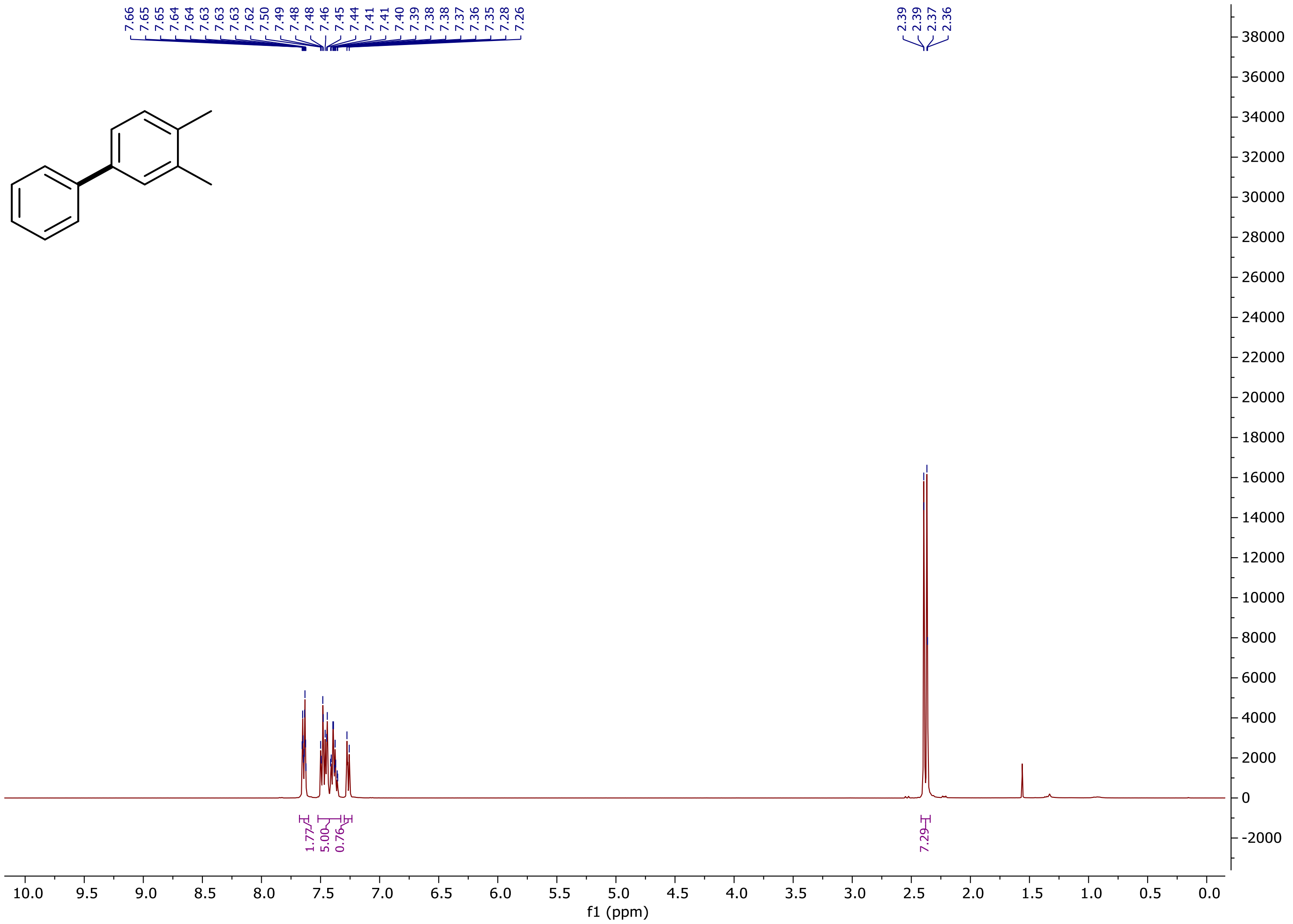

Figure S25. ^{1}H NMR (400 MHz, Chloroform-*d*) spectrum of *3,4-dimethyl-1,1'-biphenyl*.

4-Methyl-1,1'-biphenyl

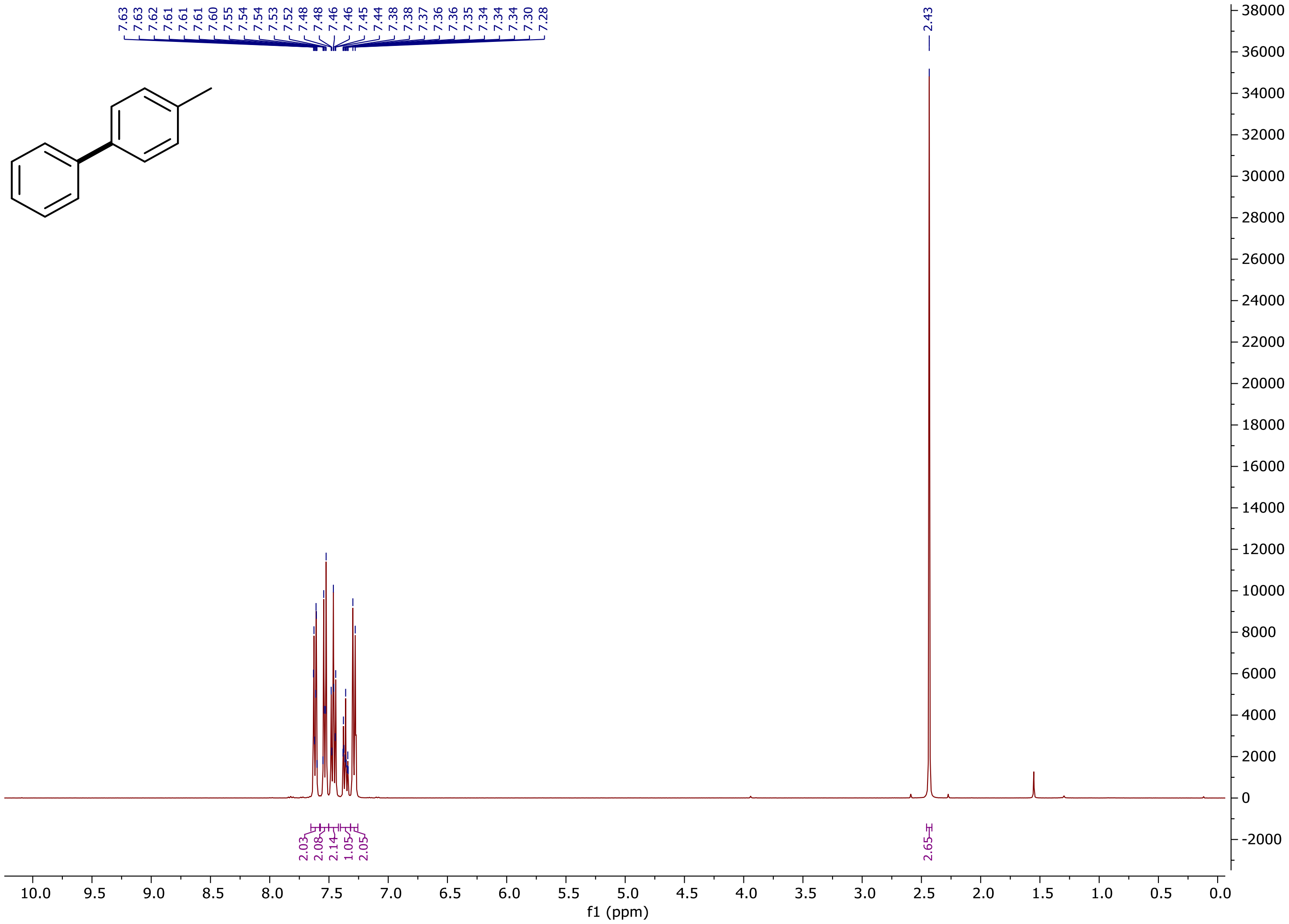

Figure S26. ^{1}H NMR (400 MHz, Chloroform-*d*) spectrum of *4-methyl-1,1'-biphenyl.*

1-Methyl-4-phenylnaphthalene

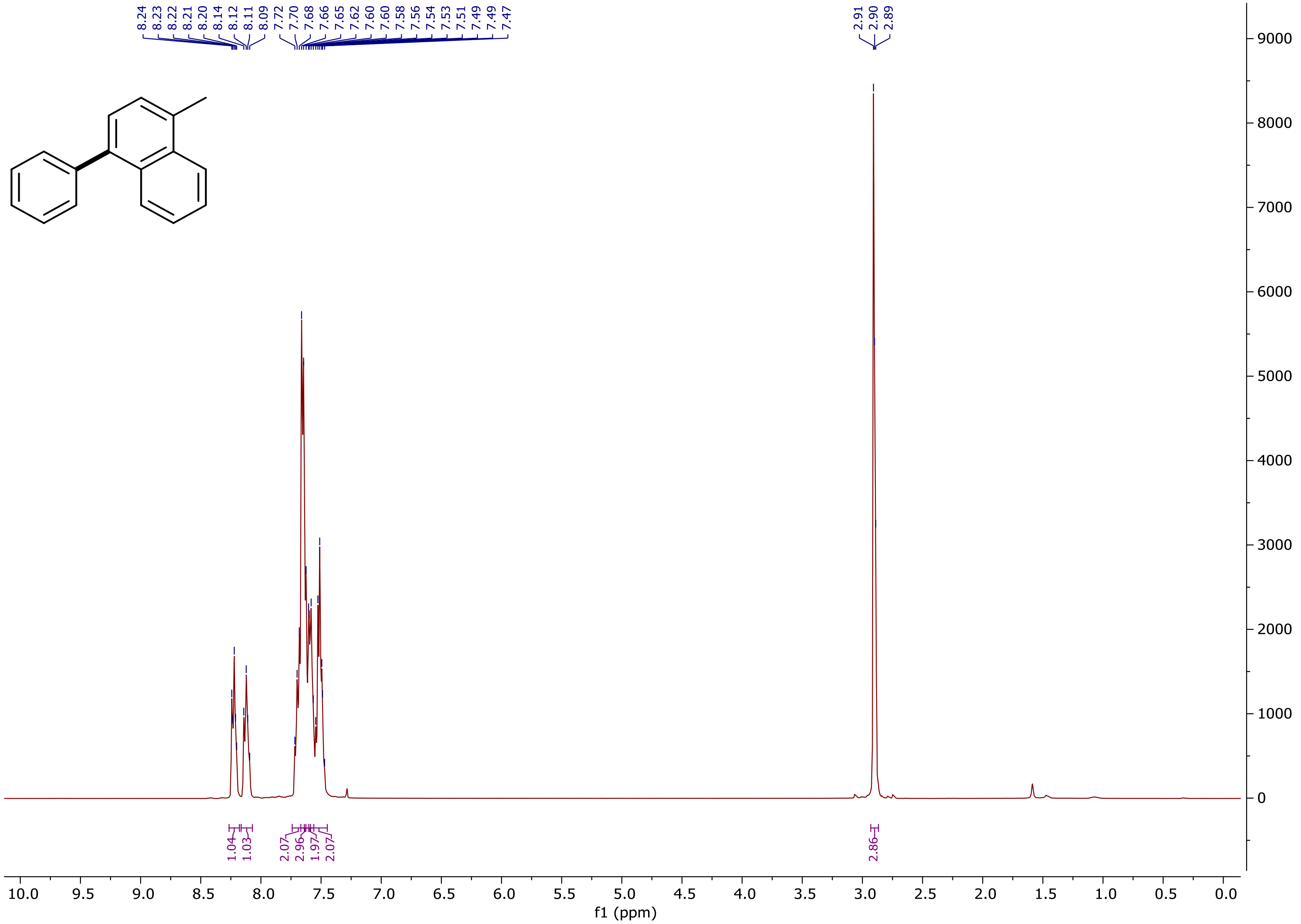

Figure S27. ^{1}H NMR (400 MHz, Chloroform-*d*) spectrum of *1-methyl-4-phenylnaphthalene*

2-Bromo-5-phenylthiophene

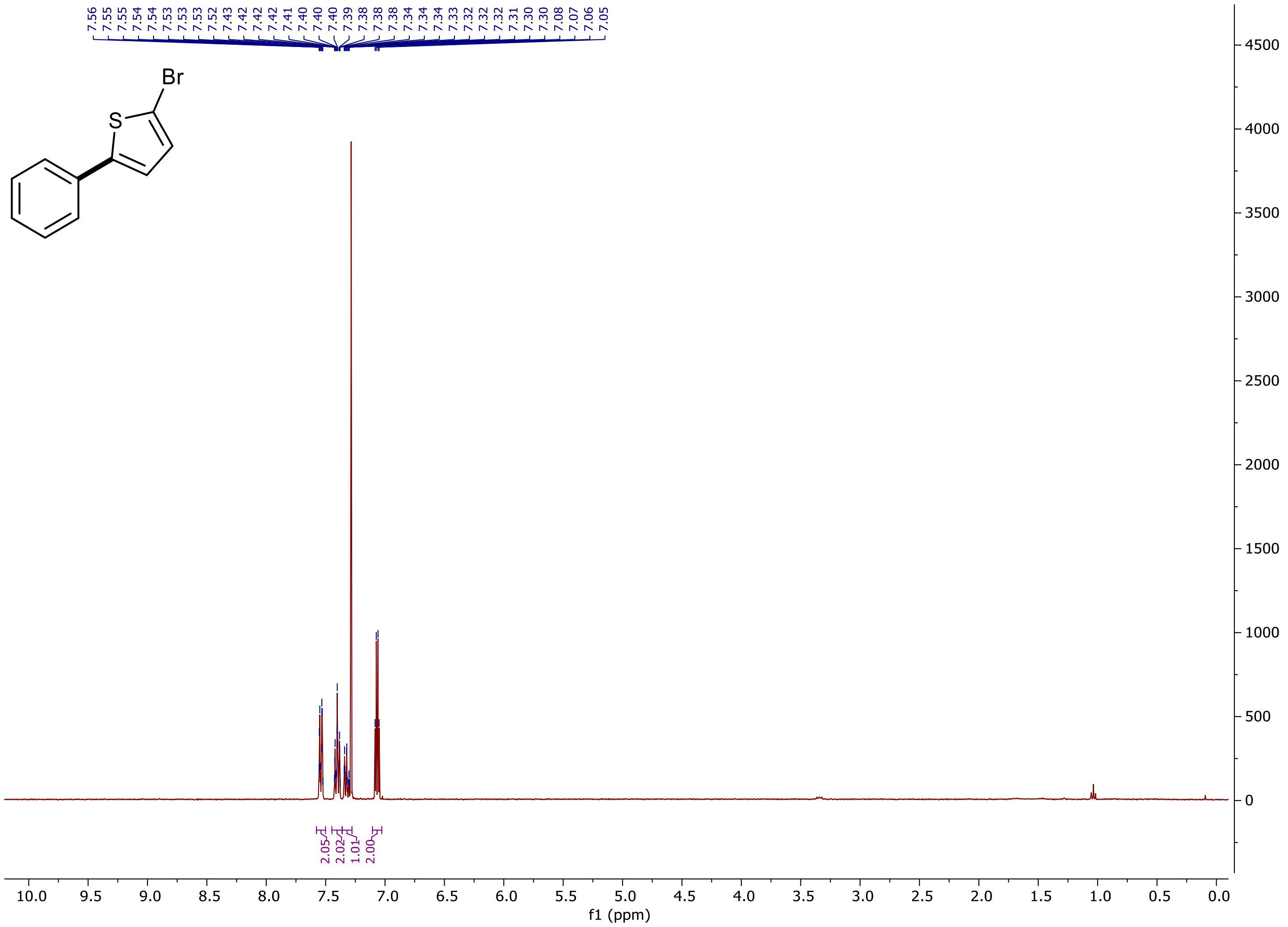

Figure S28. ^{1}H NMR (400 MHz, Chloroform-*d*) spectrum of *2-bromo-5-phenylthiophene.*

2.2. $^{13}C\{^1H\}$ NMR Spectra

N-(Prop-2-yn-1-yl)benzamide

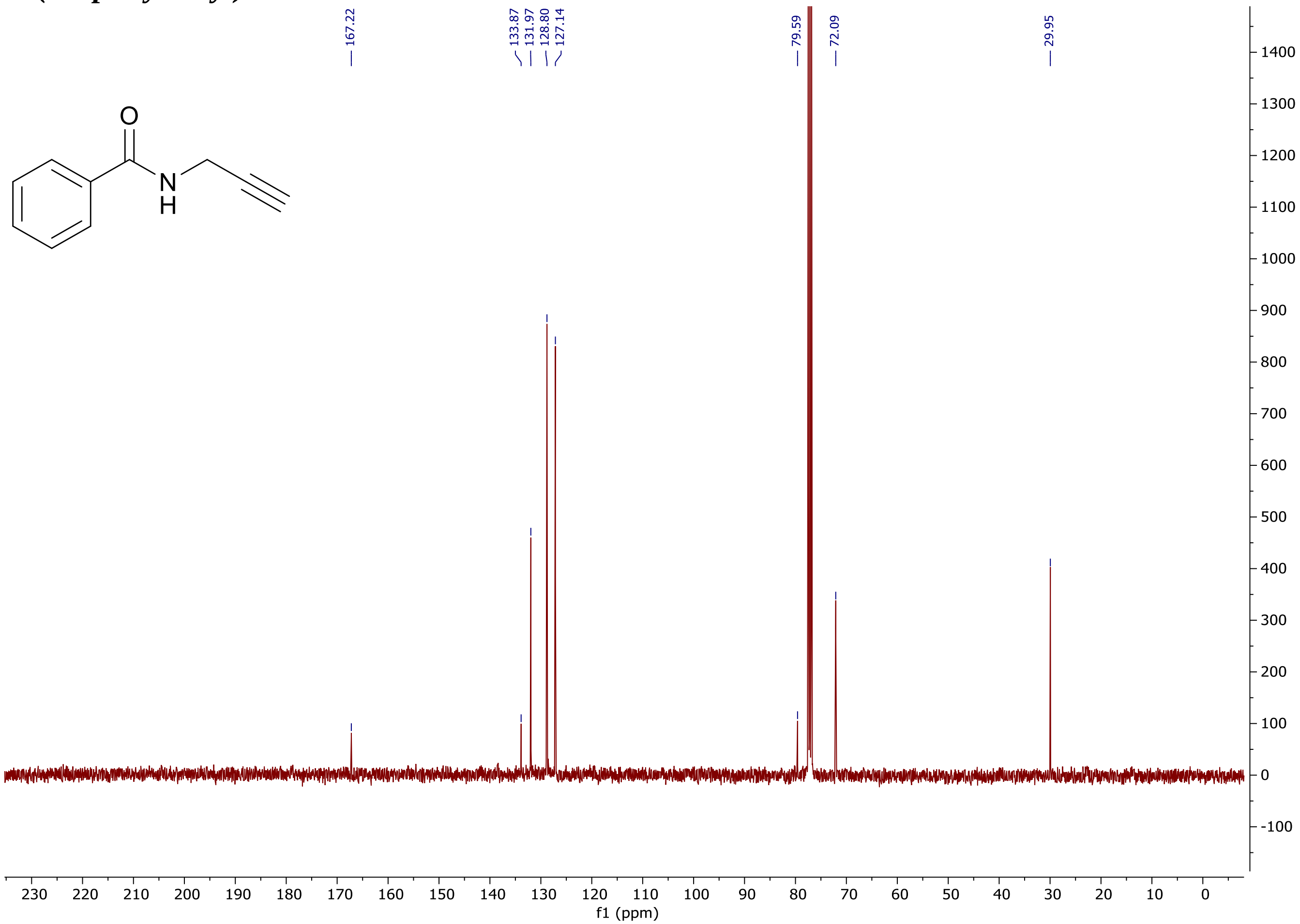

Figure S29. $^{13}C\{^1H\}$ NMR (101 MHz, Chloroform-*d*) spectrum of *N-(prop-2-yn-1-yl)benzamide.*

(Z)-5-Benzylidene-2-phenyl-4,5-dihydrooxazole

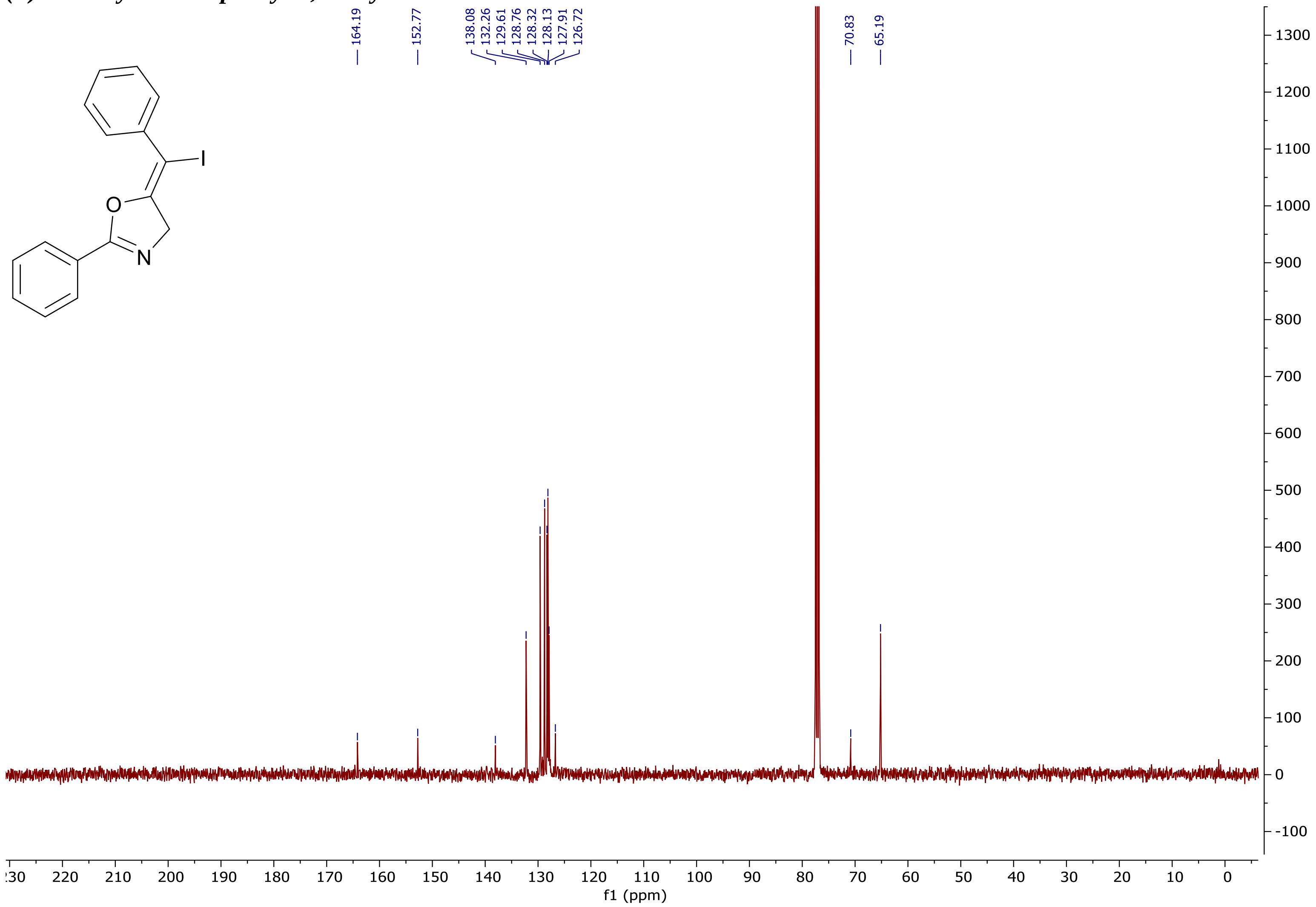

Figure S30. $^{13}C\{^{1}H\}$ NMR (101 MHz, Chloroform-*d*) spectrum of *(Z)-5-benzylidene-2-phenyl-4,5-dihydrooxazole*.

3-Chloro-4-methoxy-1,1'-biphenyl

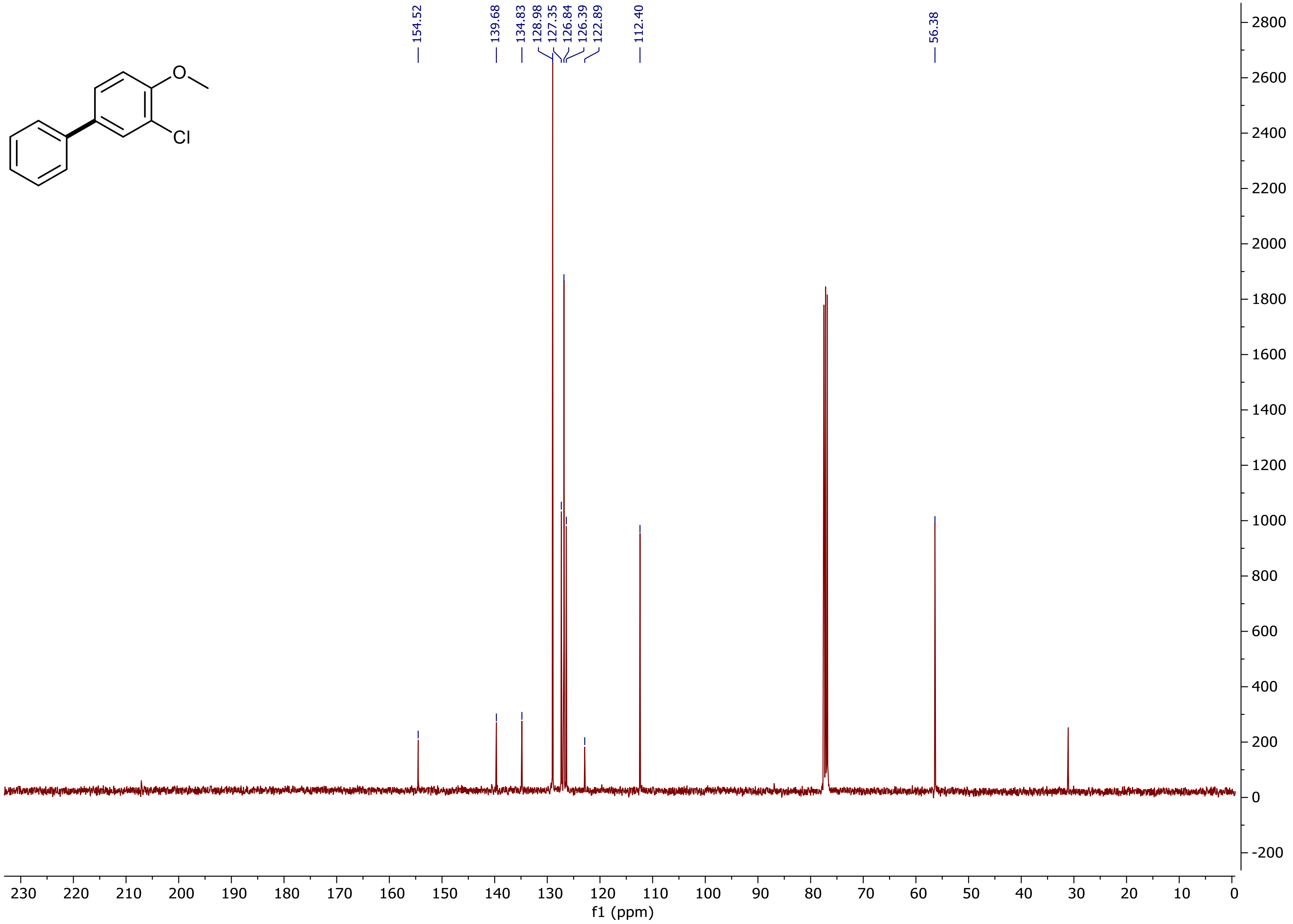

Figure S31. $^{13}C\{^{1}H\}$ NMR (101 MHz, Chloroform-*d*) spectrum of *3-chloro-4-methoxy-1,1'-biphenyl.*

3-Bromo-4-methoxy-1,1'-biphenyl

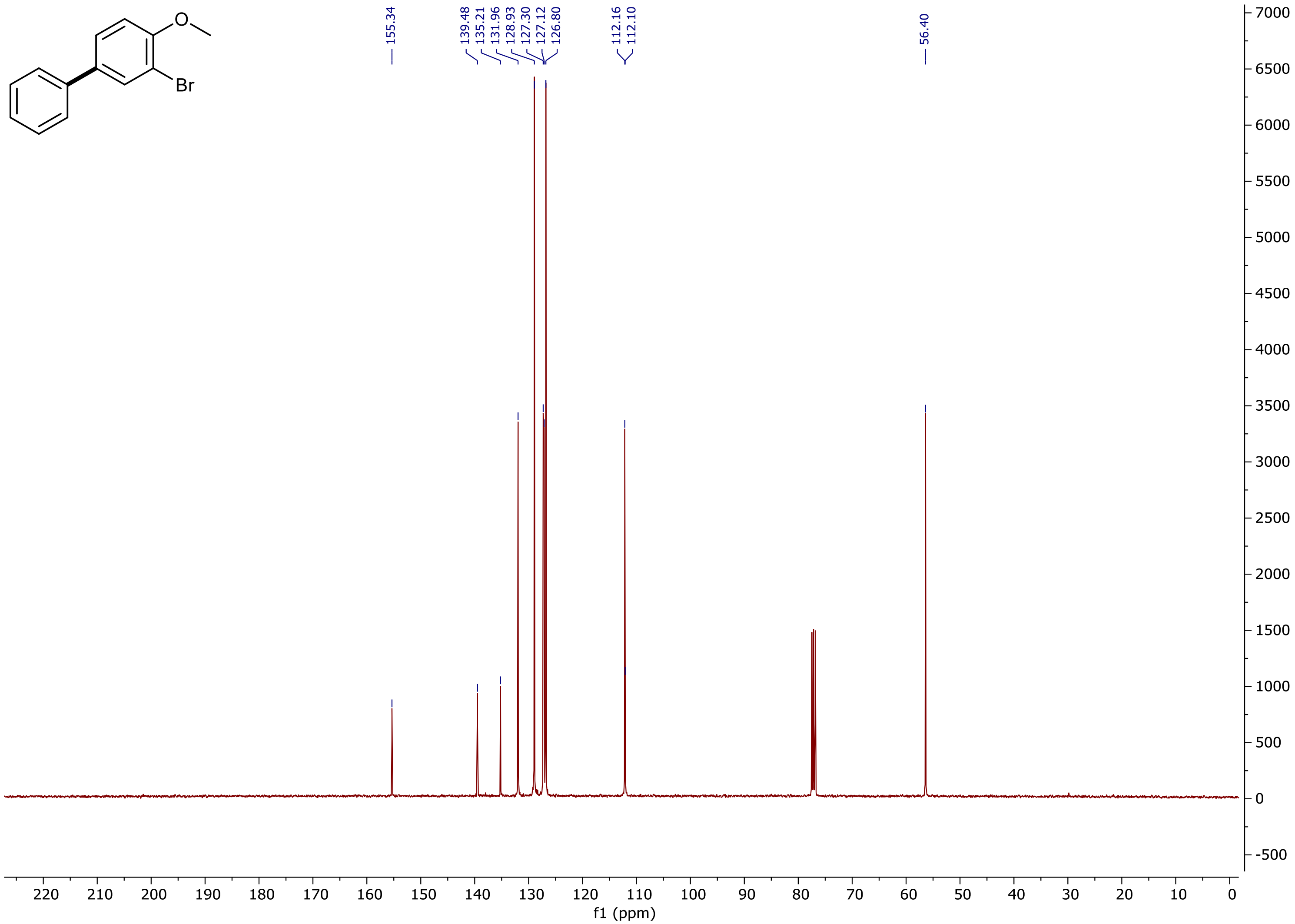

Figure S32. $^{13}C\{^{1}H\}$ NMR (101 MHz, Chloroform-*d*) spectrum of *3-bromo-4-methoxy-1,1'-biphenyl*.

3,4-Dimethyl-1,1'-biphenyl

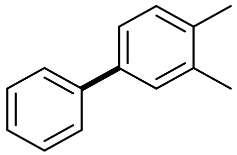

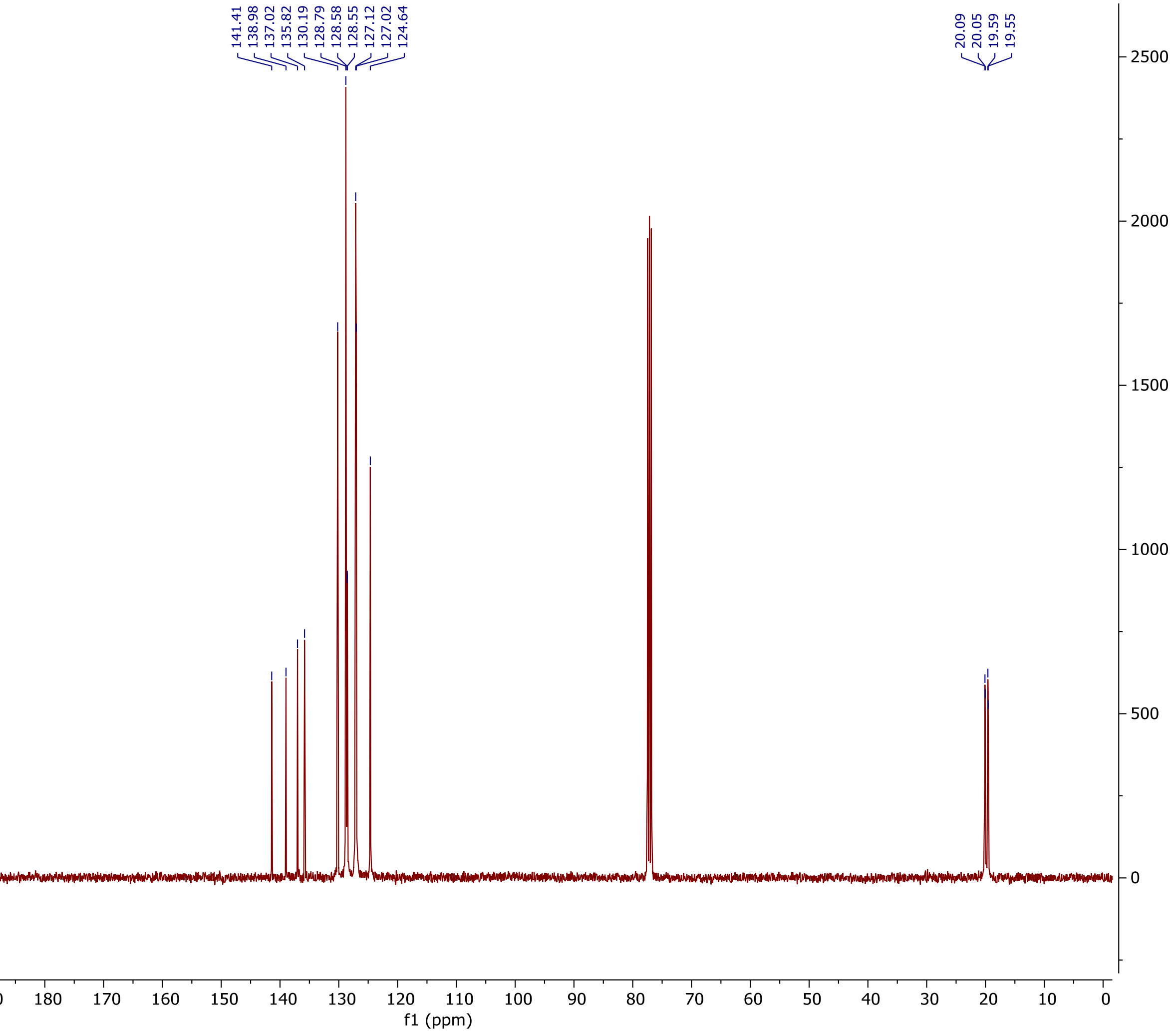

Figure S33. $^{13}C\{^{1}H\}$ NMR (101 MHz, Chloroform-*d*) spectrum of *3,4-dimethyl-1,1'-biphenyl.*

4-Methyl-1,1'-biphenyl

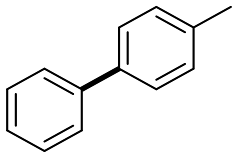

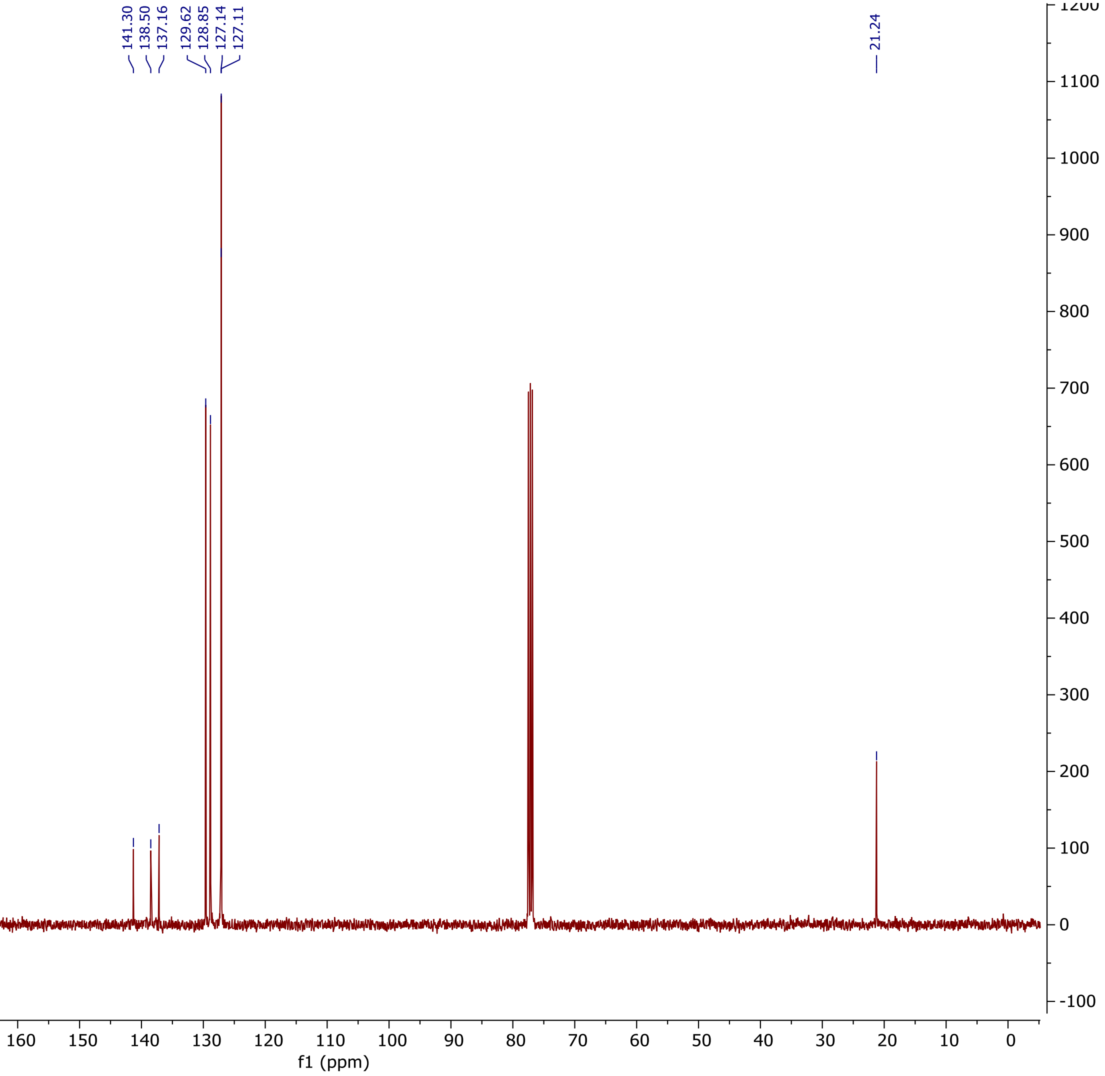

Figure S34. $^{13}C\{^{1}H\}$ NMR (101 MHz, Chloroform-*d*) spectrum of *4-methyl-1,1'-biphenyl.*

1-Methyl-4-phenylnaphthalene

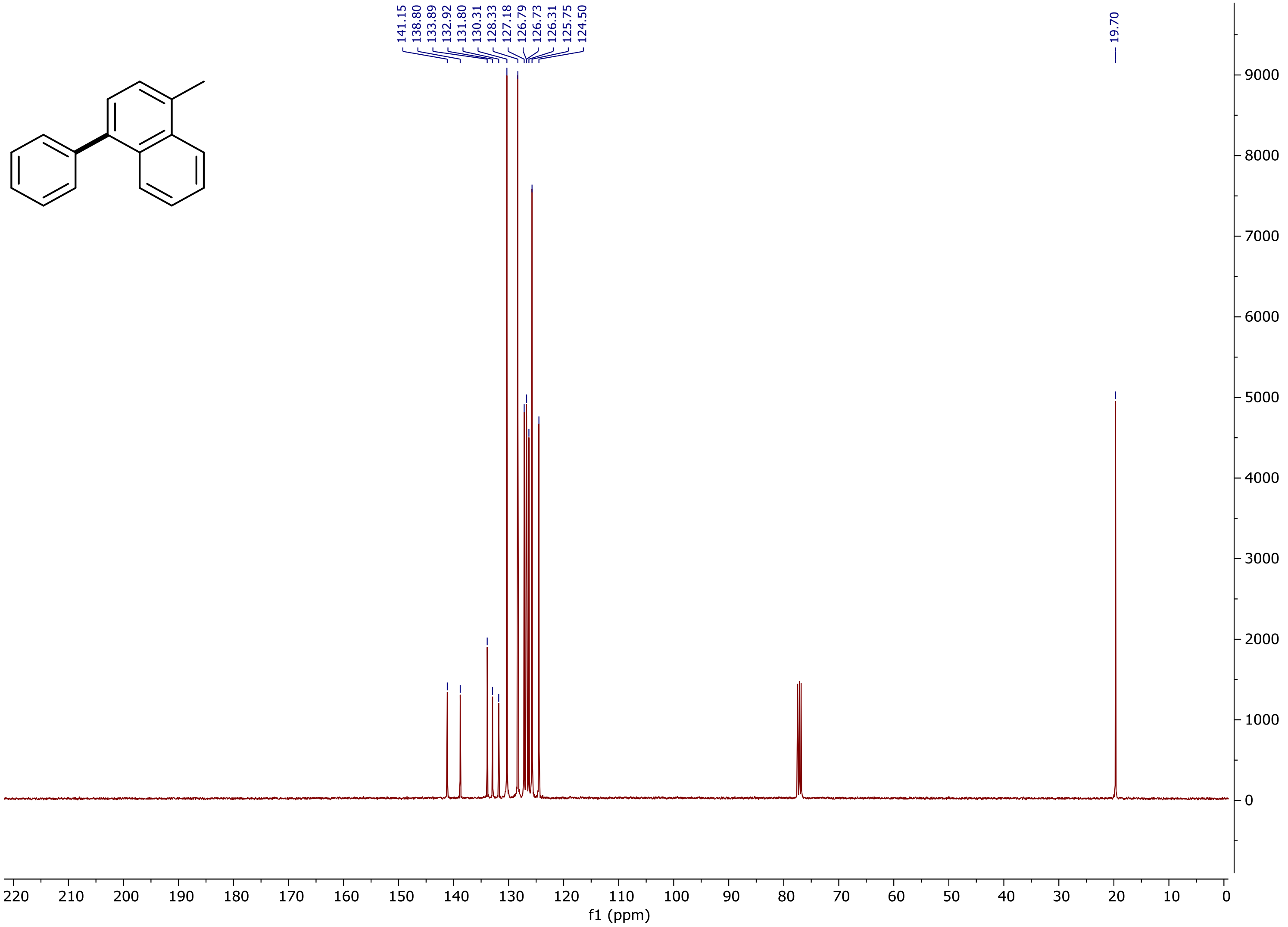

Figure S35. $^{13}C\{^{1}H\}$ NMR (101 MHz, Chloroform-*d*) spectrum of *1-methyl-4-phenylnaphthalene*.

2-Bromo-5-phenylthiophene

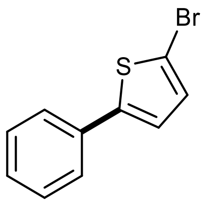

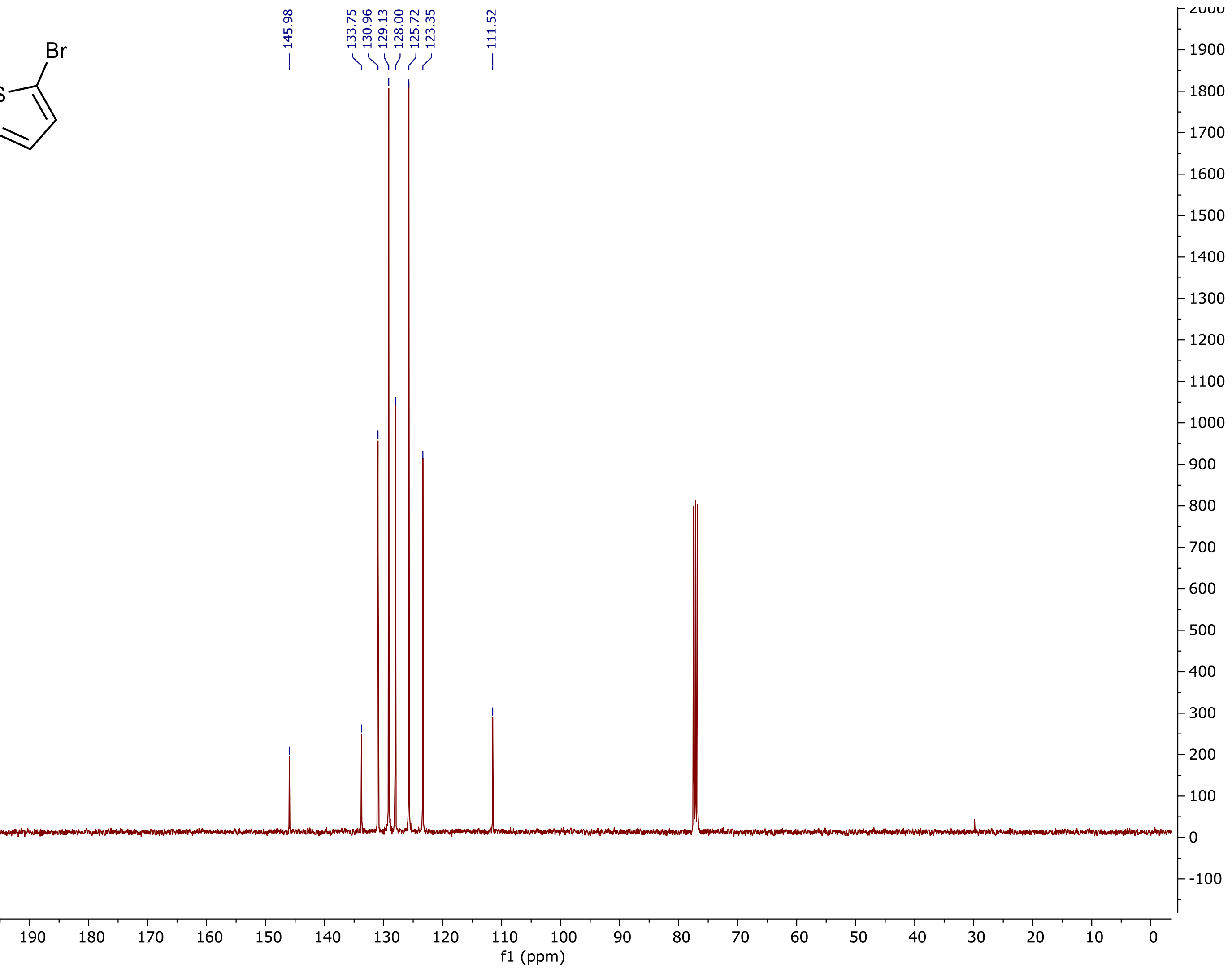

Figure S36. $^{13}C\{^1H\}$ NMR (101 MHz, Chloroform-*d*) spectrum of *2-bromo-5-phenylthiophene*.
